# Robust Estimation of Polyserial Correlation Coefficients: A Density Power Divergence Approach

**DOI:** 10.1017/psy.2026.10091

**Published:** 2026-02-10

**Authors:** Max Welz

**Affiliations:** Department of Psychology, University of Zurich, Switzerland

**Keywords:** careless responding, mixed data, model misspecification, polyserial correlation, robust estimation

## Abstract

The association between a continuous and an ordinal variable is commonly modeled through the polyserial correlation model. However, this model, which is based on a partially-latent normality assumption, may be misspecified in practice, due to, for example (but not limited to), outliers or careless responses. The typically used maximum likelihood (ML) estimator is highly susceptible to such misspecification: One single observation not generated by partially-latent normality can suffice to produce arbitrarily poor estimates. As a remedy, we propose a novel estimator of the polyserial correlation model designed to be robust against the adverse effects of observations discrepant to that model. The estimator leverages *density power divergence estimation* to achieve robustness by implicitly downweighting such observations; the ensuing weights constitute a useful tool for pinpointing potential sources of model misspecification. The proposed estimator generalizes ML and is consistent as well as asymptotically Gaussian. As price for robustness, some efficiency must be sacrificed, but substantial robustness can be gained while maintaining more than 98% of ML efficiency. We demonstrate our estimator’s robustness and practical usefulness in simulation experiments and an empirical application in personality psychology where our estimator helps identify outliers. Finally, the proposed methodology is implemented in free open-source software.

## 1 Introduction

Empirical research in the psychological, social, and health sciences often features data that contain both continuous and ordered categorical (ordinal) random variables. Examples of continuous variables are household income, blood pressure, or time spent doing an activity of interest, while examples of ordinal variables are responses to rating scales (measuring, e.g., personality traits, wellbeing, or overall health), relationship status (with categories, such as *single*, *in a relationship*, and *married*), or grouped measurements of continuous variables like one’s income group. The association between a continuous and an ordinal variable is typically modeled by means of *polyserial correlation* (Pearson, 1909). Polyserial correlation is a key building block in the analysis of mixed data, particularly structural equation models (SEMs). For instance, the popular R package lavaan (Rosseel, 2012) for SEM analyses by default uses polyserial correlation for SEMs involving both continuous and ordinal variables.

The polyserial correlation model postulates the existence of a latent continuous variable that underlies and governs the observed ordinal variable through an unobserved discretization process. The correlation between the observed continuous variable and the latent variable is called *polyserial correlation*, whereas the correlation between the observed continuous and the observed ordinal variable is called *point* polyserial correlation, where the latter can be computed from the former. For identification, the polyserial correlation model assumes that the observed continuous variable and the latent continuous variable are jointly normally distributed, thereby making it a partially-latent normality model. Estimation is typically conducted by means of maximum likelihood (ML; Cox, 1974; Olsson et al., 1982). However, the validity of the partially-latent normality assumption is often questionable in practice (e.g., Barbiero, 2025; Bedrick, 1995; Demirtas & Hedeker, 2016). Disquietingly, though, central statistical properties of polyserial correlation crucially depend on this very assumption to hold true: Violations of partially-latent normality have been shown to have potentially devastating effects on identification (e.g., Moss & Grønneberg, 2023) and ML estimation (e.g., Bedrick, 1995) of polyserial correlation. Nonnormality of (partially) latent variables can also introduce serious biases in SEM analyses conducted with software that assumes such normality (e.g., Foldnes & Grønneberg, 2020, 2022), such as lavaan (Rosseel, 2012), LISREL (Jöreskog & Sörbom, 1996), and Mplus (Muthén & Muthén, 2026).

Motivated by the susceptibility of polyserial correlation to partially-latent nonnormality, the contributions of this article are twofold. First, we study estimation of the polyserial correlation model when a (possibly empty) subset of the observed data are of low-quality and therefore may have *not* been generated by a normal distribution, such as (but not limited to) outliers in the continuous variable and/or careless responses in the ordinal variable. Consequently, such observations are uninformative for estimating the polyserial correlation model. This situation is called *partial model misspecification* because the assumption of partially-normality might be violated for parts of the data, but is satisfied for the remaining data. We demonstrate that already one single uninformative observation can suffice for ML estimation of polyserial correlation to yield arbitrary results and, furthermore, that the accuracy of ML estimation is highly susceptible to even minor misspecification of partially-latent normality. Partial misspecification stems from classic literature on robust statistics (e.g., Huber & Ronchetti, 2009) where it is known as *Huber contamination model*, owing to Huber (1964).

Second, in wake of the non-robustness of ML estimation to uninformative observations generated by partially-latent nonnormality, we propose an alternative estimator that is designed to be robust against such partial misspecification. The proposed methodology applies *density power divergence* (DPD) estimation (Basu et al., 1998) to the polyserial correlation model and achieves robustness by implicitly downweighting observations that cannot be sufficiently well fitted by that model. The ensuing weights are a useful tool for pinpointing potential sources of (partial) model misspecification. As an additional methodological contribution, we devise a simple rescaling of the weights to ensure that they are contained in the unit interval. Overall, to the best of our knowledge, the proposed methodology is the first contamination-robust approach to polyserial correlation.

In line with the partial misspecification framework, the robust estimator allows the model to be misspecified for an unknown fraction of uninformative observations, but makes *no assumption* on *how* and *where* partial misspecification occurs (which may be absent altogether). Consequently, partial misspecification can manifest through an unlimited and unrestricted variety of ways, such as (but not limited to) outliers or careless responses. Conversely, if the polyserial correlation model is correctly specified for all observations in a sample, then the robust estimator is, just like ML, consistent for the true parameter vector, and, therefore, generalizes ML estimation.

Studying their respective behavior under model misspecification, we show that both ML and the robust estimator still converge in probability, but to different limits. Crucially, the robust estimator converges to a parameter vector that is closer to the true parameter vector than ML, thereby gaining its robustness. Moreover, the robust estimator and ML remain asymptotically normally distributed, allowing for statistical inference both under correct and incorrect specification of the polyserial correlation model.

In robust statistics, there is a well-established fundamental tension between robustness and efficiency for estimation procedures for data involving continuous variables (e.g., Hampel et al., 1986; Huber & Ronchetti, 2009). Our estimator is no exception: The price to pay for its enhanced robustness manifests in the form of diminished efficiency compared to ML. However, we show that sacrificing as little as 2% of efficiency suffices to obtain a substantial gain in robustness, so the efficiency loss is only comparatively minor. An additional price of our robust estimator is increased computational intensity. Nevertheless, using our implementation, the robust estimator usually executes in less than 2 seconds on a regular laptop, so the overall computational burden should remain small in practical applications. This implementation of our proposed methodology is publicly and freely available as part of the R package robcat (for “ROBust CATegorical data analysis”; Welz et al., 2026a) on CRAN (the Comprehensive R Archive Network) at https://CRAN.R-project.org/package=robcat.

This article is organized as follows. Section 2 reviews related literature, while Section 3 summarizes the polyserial correlation model and its ML estimation. Section 4 describes the partial misspecification framework adopted in this article. Section 5 introduces our robust estimator, and Section 6 derives its theoretical and computational properties. Section 7 carries out simulation studies to compare the performance of the robust estimator to ML in a variety of settings. Section 8 provides an empirical application on data from personality psychology. Section 9 discusses and concludes.

## 2 Literature

Potential violations of the normality assumption that underlies the polyserial correlation model have been studied in previous literature, though the misspecification framework used therein is fundamentally different from the partial misspecification framework adopted in this article. Specifically, previous literature focuses on *distributional misspecification*, where the polyserial correlation model is misspecified (usually through nonnormality) for the *entire* observed sample. In contrast, in partial misspecification, the model is only misspecified for a (possibly empty) subset of the sample. We explain the differences between partial and distributional misspecification in more detail in Section 4.2.

Focusing on distributional misspecification. Bedrick (1995) shows that the accuracy of normality-based ML estimation of polyserial correlation crucially depends on whether or not the marginal distribution of the latent variable that underlies the observed ordinal variable is normal. If that distribution is not normal, but, for instance, an exponential or *t*-distribution, ML estimates of polyserial correlation may be attenuated (Brogden, 1949; Kraemer, 1981; Lord, 1963). For a dichotomous ordinal variable, Demirtas and Vardar-Acar (2017) and Demirtas and Hedeker (2016) devise an algorithm that relates polyserial correlation to point polyserial correlation when the underlying joint distribution (which they assume to be known) is not bivariate normal. For a given potentially nonnormal joint distribution and a dichotomous ordinal variable, Cheng and Liu (2016) derive a general expression for the maximum point polyserial coefficient (in population). Barbiero (2025) generalizes the results of Cheng and Liu (2016) to a polytomous ordinal variable.

Moss and Grønneberg (2023, Section 2) and Grønneberg et al. (2020, Section 2.5) use partial identification analyses to study distributional misspecification of the polyserial model from a theoretical perspective. Specifically, given known marginal distributions of the observed continuous and latent continuous variables but keeping their joint distribution unspecified, they derive partial identification sets for the partially-latent correlation. They show that the partial identification sets tend to be uninformatively wide. Consequently, polyserial correlation is very sensitive to partially-latent joint nonnormality because different nonnormal distributions can yield widely different correlations. To reduce the width of the partial identification sets, one must make restrictive assumptions on the partially-latent joint distribution, such as Gaussian-like characteristics or equipping it with a parametric structure.

In a broader context, violations of normality assumptions have been studied extensively in the psychometric literature, especially with respect to the distribution of latent variables (e.g., Asparouhov & Muthén, 2016; Lyhagen & Ornstein, 2023; Monroe, 2018; Moss & Grønneberg, 2023; Roscino & Pollice, 2006; Yuan et al., 2004, and the references therein). A particular focus of recent literature has been the *polychoric* correlation model of Pearson and Pearson (1922), which models through a latent bivariate normality distribution the association of two latent variables that govern two observed ordinal variables (see Olsson, 1979 for a modern exposition). Foldnes and Grønneberg (2020) and Jin and Yang-Wallentin (2017) show that ML estimation of polychoric correlation is highly susceptible to distributional misspecification, leading to possibly large biases in SEM analyses based on polychoric correlation (Foldnes & Grønneberg, 2022; Grønneberg & Foldnes, 2022).^1^ Welz et al. (2026b) are concerned with partial misspecification of the polychoric correlation model, where latent normality is violated due to a (possibly empty) subset of data points that were generated by an unspecified and unknown nonnormal process. We use the same (partial) misspecification framework in this article. Welz et al. (2026b) show that having in a sample already about 5% of such observations, commonly referred to as *contamination*, can suffice for a substantial estimation bias. Contamination might arise due to, for instance but not limited to, careless responding to polytomous items, which has been identified as a major threat to the validity of psychometric analyses (e.g., Alfons & Welz, 2024; Arias et al., 2020; Bowling et al., 2016; Huang et al., 2015; Meade & Craig, 2012; Ward & Meade, 2023, and references therein). As a remedy, Welz et al. (2026b) propose a fully efficient contamination-robust estimator of the polychoric correlation model that makes no assumptions on the prevalence and type of contamination (which is possibly absent altogether). Their estimator exploits the theory of *C*-estimation (Welz, 2024), being a general framework of robust estimation with categorical data.

In the context of item response theory (IRT), Itaya and Hayashi (2025) focus on the seminal model by Rasch (1960), whose estimation may be compromised by contamination due to, for instance, careless responding or random guessing. They therefore robustify marginal ML estimation thereof by using a minimum DPD estimator (Basu et al., 1998) and design a majorization–minimization algorithm for this purpose. In this article, we also utilize DPD estimation theory to robustify the (joint) ML estimation of the polyserial correlation model.

## 3 Polyserial correlation

This section defines (point) polyserial correlation and then reviews ML estimation thereof.

### 3.1 The polyserial correlation model

Suppose we observe a continuous real-valued random variable *X* with unknown population mean $\mu =\mu_{X}\in R$ and unknown population variance $\sigma^{2}=\sigma_{X}^{2}>0$ . In addition, suppose that we also observe a polytomous ordinal random variable *Y* that takes values in some finite set Y of known cardinality *r*. Without loss of generality, we assume throughout this article that Y={1,2,⋯,r} . Further assume that there exists a latent continuous random variable η governing the ordinal variable *Y* through the unobserved discretization process

(3.1) $$\begin{array}{c} Y=\backslash cases1if\;\eta <\tau_{1},2if\;\tau_{1}\leq \eta <\tau_{2},3if\;\tau_{2}\leq \eta <\tau_{3},\vdots \;rif\;\tau_{r-1}\leq \eta , \end{array}$$

where $-\infty <\tau_{1}<\tau_{2}<\cdots <\tau_{r-1}<+\infty$ are fixed but unknown *threshold* parameters. In practice, *Y* often denotes the responses to a Likert-type rating item with *r* response categories.

The primary object of interest is the fixed but unknown population correlation between the observed *X* and the latent η , denoted by

$$\begin{array}{c} \rho =Cor\left[X,\eta \right]. \end{array}$$

To identify the correlation coefficient ρ , one usually assumes that *X* and η are jointly normally distributed according to

(3.2) $$\begin{array}{c} \left(\begin{array}{cc} X & \eta \end{array}\right)∼N_{2}\left(\left(\begin{array}{cc} \mu & 0 \end{array}\right),\left(\begin{array}{ccc} \sigma^{2} & \rho \sigma \rho \sigma & 1 \end{array}\right)\right). \end{array}$$

The bivariate normality model (3.2) implies the marginal normality properties $X∼N(\mu ,\sigma^{2})$ and η∼N(0,1) . It also identifies the correlation coefficient ρ∈(−1,1) through the familiar identity $Cor\left[X,\;\eta \right]=Cov\left[X,\;\eta \right]/\sqrt{Var\left[X\right]Var\left[\eta \right]}=\rho$ . Note that since the variable η is unobserved, its population mean and variance are not jointly identifiable, which is why they are fixed to 0 and 1, respectively.

Combining the discretization model (3.1) with the bivariate normality model (3.2) yields the *polyserial correlation model* (Pearson, 1913), or, in short, polyserial model. In this model, the correlation parameter $\rho =Cor\left[X,\eta \right]$ is referred to as the *polyserial correlation coefficient*. If *Y* is dichotomous, the polyserial model reduces to the *biserial correlation model* of Pearson (1909). The theoretical properties of biserial correlation are studied by Tate (1955a, 1955b) as well as Jaspen (1946), and those of polyserial correlation by Olsson et al. (1982).

The polyserial model is subject to d=r+2 parameters, namely, the mean and variance parameters $\left(\mu ,\sigma^{2}\right)$ of the observed *X*, the polyserial correlation coefficient ρ from the normality model (3.2), as well as the r−1 thresholds from the discretization process (3.1) of the latent η . These parameters are jointly collected in a *d*-dimensional parameter vector

$$\begin{array}{c} \theta ={\left(\rho ,\mu ,\sigma^{2},\tau^{⊤}\right)}^{⊤}, \end{array}$$

where the vector $\tau ={\left(\tau_{1},\cdots ,\tau_{r-1}\right)}^{⊤}$ contains the r−1 thresholds.

Under the polyserial model evaluated at a parameter vector θ∈Θ , the density of the observed-latent pair (X,η) of continuous variables is given by

p^Xηx,v;θ=ϕ2(xv);(μ0),(σ2ρσρσ1),x,v∈R,

where $\phi_{2}(\cdot ;m,S)$ is the density of the bivariate normal distribution with population mean m and covariance matrix S . Further, denote by P^Xη⋅,⋅;θ the distribution function corresponding to the normal density p^Xη⋅,⋅;θ .

For the observed variables (X,Y) , the joint density at a realization x∈R and a response y∈Y={1,⋯,r} of the ordinal *Y* under the polyserial model at parameter θ reads

(3.3) p^XYx,y;θ=∫τy−1τyp^Xηx,v;θdv,

where we adopt the conventions $\tau_{0}=-\infty$ and $\tau_{r}=+\infty$ .^2^ The joint distribution function of the observed (X,Y) under the polyserial model can now be expressed as

(3.4) P^XYx,y;θ=PθX≤x,Y≤y=∫−∞x∑w≤yp^XYu,w;θdu=∫−∞x∫−∞τyp^Xηu,v;θdvdu,

where the third equality follows from (3.3) in conjunction with the linearity of the integral operator. As such, P^XYx,y;θ is the distribution function associated with density p^XYx,y;θ , so we refer to it as the *polyserial model distribution*.

Our expressions for the polyserial model distribution and density are different but equivalent to the more commonly used expressions in Olsson et al. (1982), which are provided in Section A of the Supplementary Material.

### 3.2 Point polyserial correlation

In addition to the correlation between the observed *X* and the latent η , one might also be interested in the correlation between *X* and the observed ordinal *Y*. The correlation between the observed *X* and *Y* is known as *point polyserial correlation*. In order to identify the desired point polyserial correlation $Cor\left[X,Y\right]$ , one needs to assign a numerical interpretation to the *r* answer categories of *Y*, that is, introduce a *scoring system*. Given a scoring system, the point polyserial correlation coefficient $\tilde{\rho }=Cor\left[X,Y\right]$ is identified by the polyserial model and can be estimated by using estimates of the polyserial model parameters θ . We provide details in Section A of the Supplementary Material.

### 3.3 Maximum likelihood estimation

Suppose we observe a sample $\{(X_{i},Y_{i}){\}}_{i=1}^{N}$ of *N* independent copies of (X,Y) generated by the polyserial model at some *true* parameter vector $\theta_{*}={\left(\rho_{*},\mu_{*},\sigma_{*}^{2},\tau_{*}^{⊤}\right)}^{⊤}$ . The statistical problem is to estimate the true $\theta_{*}$ from the observed sample, which is traditionally achieved by the ML estimator proposed by Cox (1974) and Olsson et al. (1982).

The ML estimator (MLE) of $\theta_{*}$ is defined as the log-likelihood maximizer

(3.5) $$\begin{array}{c} \hat{\theta_{N}^{MLE}}=\backslash arg\max_{\theta \in \Theta }\left.l,b,r,a,ce\sum_{i=1}^{N}\log\left(p_{XY}^{}\left(X_{i},Y_{i};\theta \right)\right),r,b,r,a\right.ce, \end{array}$$

where the parameter space

(3.6) $$\begin{array}{c} \Theta =\left.l,b,r,a,ce{\left(\rho ,\mu ,\sigma^{2},\tau^{⊤}\right)}^{⊤}|\rho \in (-1,1),\mu \in R,\sigma >0,-\infty <\tau_{1}<\cdots <\tau_{r-1}<+\infty ,r,b,r,a\right.ce \end{array}$$

is the set of legal parameters θ the MLE maximizes over. As such, Θ rules out degenerate cases, such as ρ=±1 , non-positive standard deviations (SDs), or non-monotonic thresholds. Assuming that the polyserial model is correctly specified for the data at hand, Cox (1974) and Olsson et al. (1982) show that the MLE is consistent for the true $\theta_{*}$ , asymptotically normally distributed, and fully efficient.

As a computationally attractive alternative to ML estimation, Olsson et al. (1982) propose a two-step (TS) estimation procedure. In the first step, one computes as estimators of μ , $\sigma^{2}$ , and $\tau_{k},k=1,\cdots ,r-1,$ the sample statistics

(3.7) $$\begin{array}{c} \hat{\mu_{\text{TS}}}=\frac{1}{N}\sum_{i=1}^{N}X_{i},\;\hat{\sigma_{\text{TS}}^{2}}=\frac{1}{N-1}\sum_{i=1}^{N}{\left(X_{i}-\hat{\mu_{\text{TS}}}\right)}^{2},\;\hat{\tau_{k,\text{TS}}}=\Phi^{-1}\left(\frac{1}{N}\sum_{j=1}^{k}N_{Y,j}\right), \end{array}$$

respectively, where 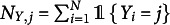 denotes the empirical marginal frequency of the *j*-th response option of *Y*. In the second step, one substitutes for these sample statistics in the log-likelihood in (3.5) and maximizes the ensuing log-likelihood with respect to the remaining parameter, ρ , via conditional ML (conditional on the sample statistics). The main advantage of the TS approach is reduced computing time because one needs to numerically solve the maximization problem (3.5) only with respect to one parameter, ρ , rather than all *d* parameters in θ . A drawback is diminished efficiency as compared to (joint) ML, where all *d* parameters are estimated simultaneously. By means of simulation experiments, Olsson et al. (1982) find that if the polyserial model is correctly specified, ML and the TS estimator produce similar results, with differences decreasing with an increasing number of response options for *Y*. The TS approach is the default estimator for polyserial correlation in the SEM software package lavaan (Rosseel, 2012).

While it is in principle possible to robustify the TS approach against contamination by using the same estimation theory as in this article, doing so would result in substantial theoretical and computational drawbacks. We discuss this in detail in Section C.4 of the Supplementary Material.

Alternative estimators of polyserial correlation have been proposed by Bedrick and Breslin (1996), Lord (1963), and Brogden (1949), with Bedrick (1990, 1992), Koopman (1983) and Kraemer (1981) studying the theoretical properties of the latter two. We discuss these approaches in more detail in Section 4.2, after conceptualizing misspecification of the polyserial model.

## 4 Model misspecification

In order to study the effects of partial model misspecification, we first define this concept and then explain how it differs from distributional misspecification.

### 4.1 Partial misspecification of the polyserial model

The polyserial model is misspecified if at least one observation for the continuous-ordinal variable pair (X,Y) has not been generated by the bivariate partially-latent normality model in (3.2). Akin to Welz et al. (2026b), we consider a *partial misspecification* framework where only a fraction (1−ε) of observations in a given sample are generated by an underlying normal distribution $P_{X\eta }$ with true parameter $\theta_{*}$ , whereas a fixed but unknown fraction ε of observations are generated from some different but unspecified underlying distribution $H_{X\eta }$ .^3^ Since $H_{X\eta }$ is unspecified, its correlation structure may differ from $P_{X\eta }$ so that observations (X,Y) generated by the underlying $H_{X\eta }$ (after discretization) may be uninformative for the true polyserial correlation coefficient.

Formally, the polyserial model is said to be *partially* misspecified if the unknown sampling distribution of the observed-latent variable pair (X,η) is given by

(4.1) Fε,Xηx,v=(1−ε)P^Xηx,v;θ∗+εHXηx,v

for x,v∈R . Correspondingly, the implied unknown sampling distribution of the observed variables (X,Y) reads

(4.2) Fε,XYx,y=(1−ε)P^XYx,y;θ∗+εHXYx,y

for x∈R,y∈Y , where

$$\begin{array}{c} H_{XY}\left(x,y\right)=\int_{-\infty }^{\tau_{\varepsilon ,y}}H_{X\eta }\left(x,dv\right) \end{array}$$

is the distribution of the observations for which the polyserial model is (partially) misspecified. The unknown and unspecified discretization thresholds $-\infty =\tau_{\varepsilon ,0}<\tau_{\varepsilon ,1}<\cdots <\tau_{\varepsilon ,r-1}<\tau_{\varepsilon ,r}=+\infty$ need not equal the true discretization thresholds $\tau_{*,1}<\cdots <\tau_{*,r-1}$ of the polyserial model. Furthermore, denote by $f_{\varepsilon ,XY}$ the unknown density corresponding to the sampling distribution $F_{\varepsilon ,XY}$ .

Misspecification models of the type in (4.1) are standard in the robust statistics literature, where they are known as *Huber contamination models*, owing to pioneering work of Huber (1964). Following Welz et al. (2026b), we therefore adopt terminology from robust statistics and call ε the *contamination fraction*, the uninformative $H_{X\eta }$ the *contamination distribution* (or simply *contamination*), and $F_{\varepsilon ,X\eta }$ the *contaminated* distribution. In the case of a zero-valued contamination fraction ( ε=0 ), there is no misspecification so that the polyserial model is correctly specified. Indeed, if ε=0 , then F0,Xη(⋅,⋅)=P^Xη⋅,⋅;θ∗ and F0,XY(⋅,⋅)=P^XY⋅,⋅;θ∗ , so the partial misspecification framework in (4.1) nests the correctly specified polyserial model.

Neither the contamination fraction ε nor the contamination distribution $H_{X\eta }$ in (4.1) is assumed to be known. Thus, both quantities are left completely unspecified in practice, which *“means that we are not making any assumptions on the degree, magnitude, or type of contamination (which is possibly absent altogether)”* (Welz et al., 2026b). Hence, the polyserial model may be misspecified due to an unlimited variety of reasons, for instance, but not limited to outliers or nonnormality in the continuous *X*, and/or careless responding or item misunderstanding in the ordinal *Y*. Because ε and $H_{X\eta }$ are unspecified in practice, the polyserial model distribution P^XY⋅,⋅;θ∗ remains the distribution of interest: Our only aim is to estimate the parameter $\theta_{*}$ of the polyserial model while reducing the impact of potential contamination in the observed data. Consequently, the contaminated distributions $F_{\varepsilon ,XY}$ and $F_{\varepsilon ,X\eta }$ are never estimated. They are purely theoretical objects to study the properties of estimators of the polyserial model when that model is partially misspecified due to data contamination.

While no assumption is made on the specific value of the contamination fraction ε , we impose the restriction ε∈0,0.5) , which is common in robust statistics (e.g., Hampel et al., 1986, p. 67). This restriction ensures proper identification: If half or more of the data points were contaminated, it would no longer be possible to distinguish between contamination and observations generated by the polyserial model, at least not without further assumptions. Under such additional assumptions, also values of ε≥0.5 could be considered. We refer to Welz et al. (2026b) for a more detailed discussion.

**Figure 1:**
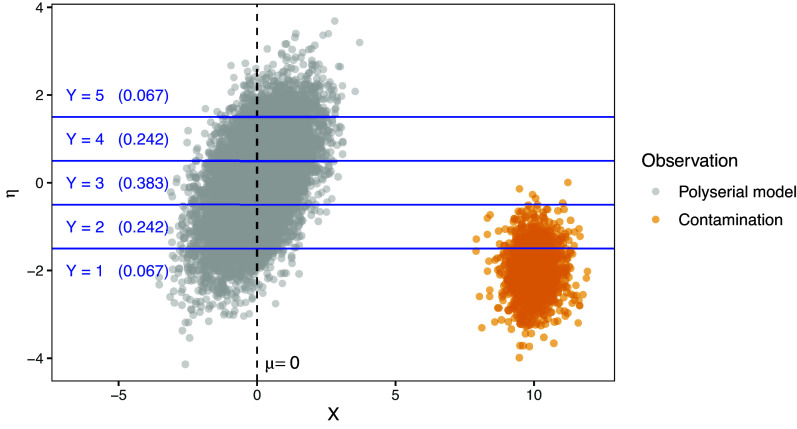
Simulated data where the polyserial model is misspecified for a fraction ε=0.15 of the N=10,000 points. The gray dots are draws of (X,η) from the polyserial model with true parameters $\rho =0.5,\mu =0,\sigma^{2}=1$ , while the orange dots are draws from a contamination distribution $H_{X\eta }$ , being a bivariate *t*-distribution here with noncentrality parameter ${\left(10,-2\right)}^{⊤}$ , scale matrix diag(0.25,0.25) , and 10 degrees of freedom. The horizontal lines mark the thresholds that discretize the latent η to the observed ordinal *Y* with five response options. The numbers in parentheses indicate the population marginal probability of the respective response option under the true polyserial model. Figure 1 long description.

Figure 1 visualizes a simulated example data set generated by the contaminated distribution $F_{\varepsilon ,X\eta }$ in (4.1) with contamination fraction ε=0.15 . In this example, the contamination distribution $H_{X\eta }$ , whose random draws are orange dots, is a shifted bivariate *t*-distribution with noncentrality parameter set to ${\left(10,-2\right)}^{⊤}$ , scale matrix diag(0.25,0.25) , and 10 degrees of freedom. The remaining realizations (gray dots) are generated by the bivariate normal distribution $P_{X\eta }$ with true parameters $\mu_{*}=0$ , $\sigma_{*}^{2}=1$ , and $\rho_{*}=0.5$ . Here, contamination manifests through somewhat larger values in the *X*-dimension and inflation of the first two response options in the *Y*-dimension (after discretization), resulting in a (partially) misspecified polyserial model. Applying the MLE in (3.5) to the observed data for (X,Y) in Figure 1 yields a sign-flipped polyserial correlation estimate of −0.522 , representing a substantial bias with respect to the true value of $\rho_{*}=0.5$ . In contrast, the robust estimator—which will be introduced in Section 5 and uses the exact same information as ML—estimates a correlation of 0.498 , which is accurate for the true value of $\rho_{*}=0.5$ .

We stress that there exist nonnormal joint distributions of (X,η) that, after discretizing the latent η variable with fixed thresholds, result in the same density for the observed (X,Y) as if the partially-latent (X,η) were jointly normal. This phenomenon is known as *discretize equivalence* of latent variables (Foldnes & Grønneberg, 2019). It follows that there exist contamination distributions $H_{X\eta }$ and contamination fractions ε>0 in (4.1) under which the contaminated density $f_{\varepsilon ,XY}$ of the observed (X,Y) is exactly equal to the polyserial model density in (3.3) at the true parameter, that is, fε,XY(x,y)=p^XYx,y;θ∗ for all (x,y)∈R×Y . Hence, in this scenario, the polyserial model is misspecified, but the misspecification does not have adverse effects because the true polyserial model density of (X,Y) remains unaffected. To avoid cumbersome notation in our analysis, we follow Welz et al. (2026b) and assume *consequential* misspecification throughout this article, that is, fε,XY(x,y)≠p^XYx,y;θ∗ for at least one response y∈Y whenever ε>0 , given x∈R . Nevertheless, *“it is silently understood that misspecification need not be consequential”* (Welz et al., 2026b), in which case there is no practical problem and both ML and robust estimator are consistent for the true $\theta_{*}$ .

Before we define our robust estimator, we briefly juxtapose the partial misspecification framework to distributional misspecification.

### 4.2 Distributional misspecification

The polyserial model is said to be *distributionally* misspecified if it is misspecified for *all* observations in a sample. This contrasts the partial misspecification framework in (4.1) where the model is only misspecified for a (possibly zero-valued) fraction of the observations. Let $G=G_{X,\eta }$ denote the unknown joint distribution of *X* and the latent η , which, under distributional misspecification, is nonnormal for all data points. In distributional misspecification, the object of interest is the correlation coefficient between *X* and η under the distribution *G*, denoted $\rho_{G}=C{or}_{G}\left[X,\eta \right]$ , instead of the correlation coefficient under bivariate normality (which would be the polyserial correlation coefficient). Although our robust estimator is designed for partial misspecification rather than distributional misspecification, it can in certain situations also offer a robustness gain under distributional misspecification. We discuss this in more detail in Section 7.3.

## 5 Robust estimation of polyserial correlation

It is well-known that ML estimation is highly susceptible to partial model misspecification due to data contamination (e.g., Hampel et al., 1986; Huber, 1964; Huber & Ronchetti, 2009), so it might be desirable to construct alternative estimators that are more robust against contamination. However, attempting to robustify estimation of the polyserial model against contamination bears the inherent challenge of how to handle the mixed-type structure of the data with one continuous and one ordinal variable.

While there exist general frameworks for robust estimation with exclusively continuous variables and exclusively categorical variables, none of them are applicable to mixed variables. On the one hand, robust *M*-estimation as proposed by Huber (1964) and related methods (e.g., Huber & Ronchetti, 2009) are intended for continuous random variables, and, broadly speaking, achieve robustness by downweighting observations with extreme values. For instance, Alfons et al. (2022) use a variation of *M*-estimation, MM -estimation, to robustify mediation analyses against outliers, heavy-tailedness, or skewness of the observed distribution. Such estimators are not applicable to mixed data because the ordinal variable cannot take extreme values due to being categorical, nor does it admit a direct numerical interpretation to begin with. On the other hand, the theory of robust *C*-estimation (Welz, 2024) is designed for exclusively categorical variables. *C*-estimation downweighs categories whose empirical frequency disagrees with their corresponding theoretical frequency under a postulated model. Since one variable in the polyserial model is continuous, it does not have discrete categories, so *C*-estimators cannot be applied. However, it turns out that the fundamental idea of downweighting data points that cannot be modeled sufficiently well by a postulated model can be exploited to achieve robustness through minimum DPD estimation (Basu et al., 1998). We explain in this section how minimum DPD estimation can be applied to the polyserial model.

For further reference, define the Kullback–Leibler (KL) divergence (Kullback & Leibler, 1951) between two bivariate densities $g_{1}$ and $g_{2}$ , each defined on $R^{2}$ (or a common subset thereof), by

(5.1) $$\begin{array}{c} KL\left(g_{1}||g_{2}\right)=\int \int g_{1}(s,t)\log\left(\frac{g_{1}(s,t)}{g_{2}(s,t)}\right)dtds, \end{array}$$

where the integrals are taken over the densities’ domain. If the second dimension of the densities corresponds to a discrete random variable, that is, the first variable is continuous, but the second one is discrete, replace the inner integral in (5.1) by a summation over the second variable’s domain.^4^

Throughout this section, suppose that one has access to a random sample of *N* independent continuous-ordinal variable pairs, $(X_{i},Y_{i}),i=1,\cdots ,N$ , following the unknown sampling distribution $F_{\varepsilon ,XY}$ in (4.2). Hence, the polyserial model is possibly misspecified for an unknown fraction ε of the observed sample.

### 5.1 Maximum likelihood revisited

The observed sample can be uniquely characterized by a particular empirical density function 
(5.2)

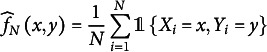

for x∈R,y∈Y , where the indicator function 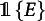 takes value 1 if an event *E* is true, and 0 otherwise. As such, the empirical density $\hat{f_{N}}$ only takes nonzero values when evaluated at points in the observed sample $\{(X_{i},Y_{i}){\}}_{i=1}^{N}$ . The MLE $\hat{\theta_{N}^{MLE}}$ in (3.5) can be expressed as the minimizer of the KL divergence between the empirical density $\hat{f_{N}}$ and model density p^XY⋅,⋅;θ , which is given by

(5.3) $$\begin{array}{c} KL\left(\hat{f_{N}}||p_{XY}^{}\left(\cdot ,\cdot ;\theta \right)\right)=\frac{1}{N}\sum_{i=1}^{N}\left(\log\left(\hat{f_{N}^{}}\left(X_{i},Y_{i}\right)\right)-\log\left(p_{XY}^{}\left(X_{i},Y_{i};\theta \right)\right)\right) \end{array}$$

and follows by definition of $\hat{f_{N}}$ and the KL divergence in (5.1) as well as the convention 0log(0)=0 .^5^ We refer to White (1982) for a detailed exposition of the connection between KL divergence and ML.

As alluded to earlier, ML estimation tends to be highly susceptible to contamination in the observed sample. It may therefore be desirable to consider alternatives that are designed to more robust against contamination. It turns out that the idea of minimizing a given divergence between the empirical density $\hat{f_{N}}$ and the model density p^XY⋅,⋅;θ can be exploited to construct contamination-robust estimators by choosing alternative divergences to the KL divergence. This is exactly what *minimum power divergence* estimation (Basu et al., 1998) does.

### 5.2 Minimum power divergence

Basu et al. (1998) propose a class of divergences between two densities that generalizes the KL divergence, but is less affected by data contamination. Suppose $g_{1}$ and $g_{2}$ are bivariate densities supported on $R^{2}$ or a common subset thereof. For a fixed tuning constant α>0 , the divergence of Basu et al. (1998) between $g_{1}$ and $g_{2}$ is defined by

$$\begin{array}{c} D_{\alpha }\left(g_{1}||g_{2}\right)=\int \int \left(g_{2}^{1+\alpha }(s,t)-\left(1+\frac{1}{\alpha }\right)g_{1}(s,t)g_{2}^{\alpha }(s,t)+\frac{1}{\alpha }g_{1}^{1+\alpha }(s,t)\right)dtds, \end{array}$$

where each integral is taken over the densities’ domain. Note that the divergence $D_{\alpha }$ is not defined at α=0 . To overcome this issue, Basu et al. (1998) define $D_{0}$ as the limit of $D_{\alpha }$ as α↓0 , that is,

(5.4) $$\begin{array}{c} D_{0}\left(g_{1}||g_{2}\right)=\lim_{\alpha ↓0}D_{\alpha }\left(g_{1}||g_{2}\right)=\int \int g_{1}(s,t)\log\left(\frac{g_{1}(s,t)}{g_{2}(s,t)}\right)dtds=KL\left(g_{1}||g_{2}\right), \end{array}$$

where the second equality follows from the identity $z\mapsto \log z=\lim_{\alpha ↓0}\alpha^{-1}(z^{\alpha }-1)$ and the third equality from the definition of the KL divergence in (5.1). Basu et al. (1998) call the divergence $D_{\alpha },\alpha \geq 0$ , the DPD. It follows that the DPD generalizes the KL divergence. We explain in a moment the intuition behind the DPD.

As before, if the second dimension of the two densities $g_{1},g_{2}$ corresponds to a discrete random variable, that is, the first variable in $g_{j}$ is continuous, but the second one is discrete, replace the inner integral in the DPD $D_{\alpha },\alpha \geq 0$ , by a summation over the second variable’s domain.

### 5.3 Proposed robust estimator

For α>0 , the DPD between the empirical density $\hat{f_{N}}$ in (5.2) and the polyserial model’s density p^XY⋅,⋅;θ reads

(5.5) $$\begin{array}{c} D_{\alpha }\left(\hat{f_{N}}||p_{XY}^{}\left(\cdot ,\cdot ;\theta \right)\right)=\int_{R}\sum_{y\in Y}p_{XY}^{1+\alpha }\left(x,y;\theta \right)dx-(1+\alpha^{-1})\frac{1}{N}\sum_{i=1}^{N}p_{XY}^{\alpha }\left(X_{i},Y_{i};\theta \right)+\alpha^{-1}, \end{array}$$

which follows from writing out $\hat{f_{N}}$ . For the choice α=0 , the DPD $D_{0}\left(\hat{f_{N}}||p_{XY}^{}\left(\cdot ,\cdot ;\theta \right)\right)$ reduces to the KL divergence in (5.3) by (5.4).

For a pre-specified tuning constant α≥0 , our proposed estimator minimizes the divergence $D_{\alpha }$ between $\hat{f_{N}}$ and p^XY⋅,⋅;θ with respect to the model parameter θ∈Θ . Specifically, our proposed estimator is given by

(5.6) $$\begin{array}{c} \hat{\theta_{N}}=\backslash arg\min_{\theta \in \Theta }D_{\alpha }\left(\hat{f_{N}}||p_{XY}^{}\left(\cdot ,\cdot ;\theta \right)\right). \end{array}$$

In particular, if α=0 , the estimator $\hat{\theta_{N}}$ coincides with the MLE $\hat{\theta_{N}^{MLE}}$ (see Section 5.1). Since it minimizes a DPD, estimator $\hat{\theta_{N}}$ constitutes a *minimum DPD estimator* (Basu et al., 1998). As such, a limit theory for $\hat{\theta_{N}}$ is readily available from Basu et al. (1998), who derive the theoretical properties of such estimators for general models. Before we turn to the estimator’s limit theory, we first provide some intuition as to *why* minimum DPD estimators are more robust than ML whenever α>0 .

An estimator defined as the minimum of a (differentiable) loss function can be equivalently characterized as a root of the loss’ gradient; a characterization known as *estimating equation*. The estimating equation of the MLE (α=0) can be written as

$$\begin{array}{c} \frac{1}{N}\sum_{i=1}^{N}u_{\theta }\left(X_{i},Y_{i}\right)=0, \end{array}$$

which is satisfied for $\theta =\hat{\theta_{N}^{MLE}}$ , and where the *d*-vector

uθx,y=∂∂θlogp^XYx,y;θ,x∈R,y∈Y,

denotes the log-likelihood *score* function. A closed-form expression of $u_{\theta }\left(x,y\right)$ is provided in Section A of the Supplementary Material. Analogously, the estimating equation of the proposed estimator $\hat{\theta_{N}}$ in (5.6) can be shown to read

(5.7) $$\begin{array}{c} \frac{1}{N}\sum_{i=1}^{N}p_{XY}^{\alpha }\left(X_{i},Y_{i};\theta \right)u_{\theta }\left(X_{i},Y_{i}\right)-c_{\alpha }(\theta )=0, \end{array}$$

which is satisfied for $\theta =\hat{\theta_{N}}$ and where $c_{\alpha }(\theta )=\int_{R}\sum_{y\in Y}p_{XY}^{1+\alpha }\left(x,y;\theta \right)u_{\theta }\left(x,y\right)dx$ is a correction factor independent of observed data. Observe that for α=0 , the estimating equation (5.7) equals that of the MLE,^6^ in which all observations in the sample $\{(X_{i},Y_{i}){\}}_{i=1}^{N}$ are weighted equally. Conversely, when α>0 , the term

$$\begin{array}{c} \frac{1}{N}\sum_{i=1}^{N}p_{XY}^{\alpha }\left(X_{i},Y_{i};\theta \right)u_{\theta }\left(X_{i},Y_{i}\right) \end{array}$$

in estimating equation (5.7) *“provides a relative-to-the-model downweighting for [contaminated]* ^7^ *observations”* (Basu et al., 1998, p. 551) with individual-specific weights

(5.8) $$\begin{array}{c} \tilde{w_{i,\alpha }}(\theta )=p_{XY}^{\alpha }\left(X_{i},Y_{i};\theta \right). \end{array}$$

A weight $\tilde{w_{i,\alpha }}(\theta )$ is bounded from below by 0 and takes values close to 0 for observations that the polyserial model cannot fit well, such as contaminated data points. As such, *“observations that are widely discrepant to the [polyserial] model will get nearly zero weights”* (Basu et al., 1998, pp. 551–552). Larger choices of α>0 lead to more stringent downweighting. For instance, a contaminated observation with an ordinal response $Y_{i}$ that strongly disagrees with the latent threshold structure implied by the model density $p_{XY}$ would receive a weight close to 0.

If contamination is absent (ε=0) , it can be shown that the robust estimator $\hat{\theta_{N}}$ and the MLE $\hat{\theta_{N}^{MLE}}$ in (3.5) are asymptotically equal to one another for all choices α≥0 . This property is implied by the property of *Fisher consistency* that minimum DPD estimators possess (implied by Theorem 1 in Basu et al., 1998, see page 551 therein).

Being expressible as a root of a summation over the sample where each summand is a certain function evaluated at a single observation (Eq. (5.7)), minimum DPD estimators are a special case of *M*-estimation (see Basu et al., 1998, Section 3.1). However, unlike classic *M*-estimation as proposed in Huber (1964), minimum DPD estimation does not require the data to admit a numerical interpretation due to operationalizing “outlyingness” through discrepant model fit rather than extreme values. Consequently, it can be directly applied to models for mixed data, such as the polyserial model.

### 5.4 Rescaling of weights

By construction, for a given tuning constant α>0 , the robust estimator’s raw weights $\tilde{w_{i,\alpha }}(\theta )$ in (5.8) are bounded from below by 0 and from above by some data-independent finite value $M_{\alpha }(\theta )$ only depending on α and θ , which is defined by Mα(θ)=sup_^pXYαx,y;θ:x∈R,y∈Y} . The fact that the upper bound $M_{\alpha }(\theta )$ varies for different values of θ and α makes it difficult to compare raw weights across different estimates and specifications. Since the raw weights are intended as a tool for detecting contaminated observations, it would be beneficial to have such a comparability. In the following, we describe a simple novel procedure to rescale the weights to always be bounded from above by 1. Rescale the raw weights by their upper bound $M_{\alpha }(\theta )$ to obtain

(5.9) $$\begin{array}{c} w_{i,\alpha }(\theta )=\tilde{w_{i,\alpha }}(\theta )/M_{\alpha }(\theta ),\;i=1,\cdots ,N. \end{array}$$

We henceforth refer to the rescaled weights $w_{i,\alpha }(\theta )$ simply as *weights*. The rescaled weights are contained in the interval 0,1 and take values close to 0 for poorly fitting observations and value 1 for perfectly fitting observations. This rescaling is without loss of generality because the estimating equation (5.7) can equivalently be expressed as

$$\begin{array}{c} \frac{1}{N}\sum_{i=1}^{N}w_{i,\alpha }(\theta )u_{\theta }\left(X_{i},Y_{i}\right)-c_{\alpha }(\theta )/M_{\alpha }(\theta )=0. \end{array}$$

Section B of the Supplementary Material describes an algorithm for computing the upper bound $M_{\alpha }(\theta )$ . The usefulness of the rescaled weights as a tool for identifying contaminated data points will be demonstrated in an empirical application in Section 8, where they successfully detect observations discrepant from the model.

## 6 Statistical and computational properties

This section is devoted to the properties of minimum DPD estimators for estimating polyserial models. We first discuss their estimands under contamination, then their asymptotic properties and efficiency properties, and then computational aspects as well as our software implementation.

### 6.1 Estimand

It is instructive to discuss what the proposed minimum DPD estimator $\hat{\theta_{N}}$ in (5.6) actually estimates. Recall that $f_{\varepsilon ,XY}$ denotes the density of the sampling distribution $F_{\varepsilon ,XY}$ in (4.2) that the observed sample follows. Just like $F_{\varepsilon ,XY}$ , the population density $f_{\varepsilon ,XY}$ is completely unspecified and unknown in practice, including contamination fraction ε . For a fixed tuning constant α≥0 , the estimand $\theta_{0}$ of estimator $\hat{\theta_{N}}$ is the parameter that minimizes the DPD between the population density and the polyserial model density, that is,

$$\begin{array}{c} \theta_{0}=\backslash arg\min_{\theta \in \Theta }D_{\alpha }\left(f_{\varepsilon ,XY}||p_{XY}^{}\left(\cdot ,\cdot ;\theta \right)\right). \end{array}$$

Observe that $D_{\alpha }\left(f_{\varepsilon ,XY}||p_{XY}^{}\left(\cdot ,\cdot ;\theta \right)\right)$ is simply the population analog of the empirical divergence $D_{\alpha }\left(\hat{f_{N}}||p_{XY}^{}\left(\cdot ,\cdot ;\theta \right)\right)$ that is minimized by the estimator $\hat{\theta_{N}}$ in (5.6).

In the absence of contamination ( ε=0 ), one has that f0,XY(⋅,⋅)=p^XY⋅,⋅;θ∗ by (4.2), and it follows from Theorem 1 in Basu et al. (1998) that $\theta_{0}=\theta_{*}$ , so that the population divergence is zero-valued. Subsequently, just like the MLE, $\hat{\theta_{N}}$ is unbiased for the true $\theta_{*}$ (in population) if the model is correctly specified. This property is known as *Fisher consistency*. We stress that Fisher consistency holds true for *all* choices of α≥0 . In other words, if contamination is absent, then the proposed estimator is unbiased (in population) no matter the choice of tuning constant α .

On the other hand, if contamination is present (ε>0) , then the estimand $\theta_{0}$ will generally differ from the true $\theta_{*}$ . How much they differ depends on the contamination fraction ε and contamination type $H_{X\eta }$ in the contaminated distribution (4.1), as well as the choice of tuning constant α≥0 . Roughly speaking, holding the unknown contaminated distribution $F_{\varepsilon ,X\eta }$ constant, the larger α , the closer $\theta_{0}$ tends to be to the true $\theta_{*}$ .^8^ In other words, if the polyserial model is misspecified, larger values of α asymptotically tend to lead to less bias for the true $\theta_{*}$ .

### 6.2 Asymptotic analysis

We are now ready to study the asymptotic properties of the proposed estimator $\hat{\theta_{N}}$ in (5.6). Under mild standard regularity conditions, it can be shown that $\hat{\theta_{N}}$ is asymptotically consistent for estimand $\theta_{0}$ , that is,

$$\begin{array}{c} \hat{\theta_{N}}\backslash stackrelP⟶\theta_{0}, \end{array}$$

as N→∞ , and is furthermore asymptotically Gaussian,

$$\begin{array}{c} \sqrt{N}\left(\hat{\theta_{N}}-\theta_{0}\right)\backslash stackreld⟶N_{d}\left(0,\Sigma \left(\theta_{0}\right)\right), \end{array}$$

as N→∞ , where “ \stackrelP⟶ ” and “ \stackreld⟶ ” denote convergence in probability and distribution, respectively. These two stochastic convergence results are rigorously established in Theorem A.1 in the Supplementary Material. The theorem follows immediately from a more general result in Basu et al. (1998). The asymptotic covariance matrix $\Sigma \left(\theta_{0}\right)$ has a closed-form sandwich-type construction

(6.1) $$\begin{array}{c} \Sigma \left(\theta_{0}\right)=J{\left(\theta_{0}\right)}^{-1}K\left(\theta_{0}\right)J{\left(\theta_{0}\right)}^{-1}, \end{array}$$

where the d×d full-rank matrices $\theta \mapsto J\left(\theta \right),K\left(\theta \right)$ are defined in Section A of the Supplementary Material. Neither $J\left(\theta \right)$ nor $K\left(\theta \right)$ are observed in practice, so neither is $\Sigma \left(\theta_{0}\right)$ , but each of these matrices can be consistently estimated, as we will explain momentarily. Owing to this asymptotic normality result, one can construct standard errors and confidence intervals for the estimand $\theta_{0}$ . We stress that the estimator remains asymptotically normal even when the polyserial model is misspecified. Similar results for inference in general misspecified models with sandwich-type covariance constructions have been derived by, for example, White (1982) and Huber (1967) for the MLE, Basu et al. (1998) for robust minimum DPD estimators (of which our proposed estimator is a special case), and Welz (2024) for a class of robust estimators for general categorical data.

Being a function of the unobserved estimand $\theta_{0}$ as well as additional unobserved population quantities, the population covariance matrix $\Sigma \left(\theta_{0}\right)$ in (6.1) is also unobserved in practice. Nevertheless, we can consistently estimate it by constructing certain estimators $\hat{J_{N}}\left(\theta \right)$ and $\hat{K_{N}}\left(\theta \right)$ , which are pointwise consistent for $J\left(\theta \right)$ and $K\left(\theta \right)$ , respectively, for a given parameter vector θ∈Θ . Since both estimators are continuous in θ , it follows from the continuous mapping theorem that the sample analog of the sandwich-type construction in (6.1)

$$\begin{array}{c} \hat{\Sigma_{N}}\left(\theta \right)=\hat{J_{N}}{\left(\theta \right)}^{-1}\hat{K_{N}}\left(\theta \right)\hat{J_{N}}{\left(\theta \right)}^{-1} \end{array}$$

is pointwise consistent for $\Sigma \left(\theta \right)$ . Combining this result with the convergence result $\hat{\theta_{N}}\backslash stackrelP⟶\theta_{0}$ , the plug-in estimator $\hat{\Sigma_{N}}\left(\hat{\theta_{N}}\right)$ is consistent for the asymptotic covariance matrix $\Sigma \left(\theta_{0}\right)$ . We refer to Section A of the Supplementary Material for the definition of $\hat{J_{N}}\left(\theta \right)$ and $\hat{K_{N}}\left(\theta \right)$ as well as details.

### 6.3 Efficiency

For the tuning constant α=0 , which corresponds to the MLE $\hat{\theta_{N}^{MLE}}$ , it can be shown (see Section A.5 of the Supplementary Material) that if the polyserial model is correctly specified ( ε=0 ), the asymptotic covariance matrix in (6.1) reduces to the inverted *Fisher information* of the polychoric model, $I_{\theta_{0}}^{-1}$ , where

Iθ=∫R∑y∈Yp^XYx,y;θuθx,yuθx,y⊤dx

denotes the Fisher information of the polyserial model at θ∈Θ . Hence, (6.1) nests the well-known result that the MLE’s asymptotic covariance matrix is equal to the inverted Fisher information matrix. It is furthermore well-known that ML estimation is fully efficient, meaning that no unbiased estimator has a smaller variance (e.g., Theorem 3.10 in Lehmann & Casella, 1998).

In contrast, for strictly positive tuning constants α>0 , the asymptotic covariance matrix of the corresponding estimator $\hat{\theta_{N}}$ in (6.1) is generally *not* equal to the inverse Fisher information matrix at the polyserial model. Thus, recalling the full efficiency property of the MLE, our robust estimator is *less* efficient than the MLE. The efficiency loss is due to downweighting of observations with low probability under the polyserial model, which happens even when the model is correctly specified. Consequently, while the robust estimator and MLE are both consistent and unbiased for the true parameter value as long as contamination is absent, the former has a larger estimation variance.

To quantify the efficiency loss, we calculate at the population level the relative efficiency of our robust estimator compared to the MLE when the polyserial model is correctly specified ( ε=0 ). Recall that in this zero-contamination case, the estimand $\theta_{0}$ is equal to the true parameter value $\theta_{*}$ for all α≥0 due to the property of Fisher consistency. For a given true value $\theta_{*}$ , the *relative efficiency* (e.g., Van der Vaart, 1998, Chapter 8.2) of our estimator for the true polyserial correlation $\rho_{*}$ with respect to the fully efficient MLE is given by

$$\begin{array}{c} \frac{Var\left[\hat{\rho_{N}^{MLE}}\right]}{Var\left[\hat{\rho_{N}}\right]}={\left(I_{\theta_{*}}^{-1}\right)}_{1,1}/{\left(\Sigma \left(\theta_{*}\right)\right)}_{1,1}, \end{array}$$

where the operator ${\left(\cdot \right)}_{1,1}$ picks out the top left element of a matrix. The efficiency loss of minimum DPD estimators can also be expressed as a function of α for a given model (see Basu et al., 1998, Section 4.2).

Table 1 lists the relative efficiencies of various choices of the tuning constant α for an ordinal *Y* with r=5 response options and true parameter vector $\theta_{*}={\left(\rho_{*},\mu_{*},\sigma_{*}^{2},\tau_{*,1},\tau_{*,2},\tau_{*,3},\tau_{*,4}\right)}^{⊤}={\left(0.5,0,1,-1.5,-0.5,0.5,1.5\right)}^{⊤}$ . Up to about α=0.25 , the relative efficiency of the robust estimator stays above 90%, and then decreases roughly linearly to slightly below 50% for α=1 . Choices of α>1 , while yielding even more robustness, would result in a comparatively poor relative efficiency of considerably less than 50% and are therefore not considered in this article. The efficiency results for other choices of the true parameter vector $\theta_{*}$ are very similar and are therefore deferred to Section C of the Supplementary Material. Our software implementation, discussed in more detail in Section 6.4, provides functionality to compute the relative efficiency at arbitrary user-specified parameter vectors and tuning constants α .

**Table 1:** Relative efficiency for estimating the polyserial correlation coefficient $\rho_{*}$ when *Y* has r=5 response options, for various choices of the tuning constant α at a true parameter vector $\theta_{*}={\left(\rho_{*},\mu_{*},\sigma_{*}^{2},\tau_{*,1},\tau_{*,2},\tau_{*,3},\tau_{*,4}\right)}^{⊤}={\left(0.5,0,1,-1.5,-0.5,0.5,1.5\right)}^{⊤}$ Table 1 long description.

α	0 (MLE)	0.1	0.25	0.5	0.75	1
rel. efficiency	1.000	0.983	0.916	0.762	0.612	0.488

A loss of efficiency is a common property of many robust estimators involving continuous random variables, and, as demonstrated in this section, our proposed estimator is no exception.^9^ As such, our estimator is based on a compromise between efficiency and robustness. Borrowing an insurance metaphor from Anscombe (1960), we *“sacrifice some efficiency at the model, in order to insure against accidents caused by deviations from the model”* (Huber & Ronchetti, 2009, p. 5). However, it turns out that the efficiency loss is relatively minor for reasonably small positive tuning constants like α=0.1 (Table 1), which, as the simulation experiments in Section 7 will reveal, suffices to gain substantial robustness over ML estimation.

### 6.4 Implementation

We provide an open-source implementation of our robust estimator as part of the package robcat (for “ROBust CATegorical data analysis”; Welz et al., 2026a) for the statistical programming environment R (R Core Team, 2024). The package is freely available from CRAN (the Comprehensive R Archive Network) at https://CRAN.R-project.org/package=robcat. We used this package for obtaining all numerical results in this article.

In principle, every suitable method for numerical optimization can be used to minimize the robust estimator’s minimization problem in (5.6). In our experience, unconstrained optimization via the BFGS algorithm (e.g., Nocedal & Wright, 2006, Section 6.1) works well. Yet, additional stability could be gained from making explicit the constraints in the parameter space Θ in (3.6), for which standard algorithms for constrained optimization can be used, such as the simplex method of Nelder and Mead (1965). In our implementation, we adopt a similar default behavior as the implementation of robust polychoric correlation estimation (Welz et al., 2026b) in package robcat. Specifically, the implementation first tries unconstrained optimization with the BFGS algorithm. If instability or numerical nonconvergence are encountered or a constraint is violated, it instead uses the algorithm of Nelder and Mead (1965) for constrained optimization.^10^ Nevertheless, numerous other optimization algorithms are supported, and users can freely choose their preferred option.

As for the choice of tuning constant α≥0 in the DPD function (5.5), higher values lead to greater robustness but less efficiency, whereas values closer to 0 are less robust but more efficient. We shall see in the simulation experiments in the next section that the choice α=0.5 constitutes a good compromise between robustness against contamination up to about ε=0.3 while being reasonably efficient with about 76% of the MLE’s efficiency (Table 1). Therefore, α=0.5 is the default choice in our implementation. While this is the default choice, we acknowledge that there might be situations in which choices that lead to more (or less) robustness are more appropriate, so we advise users to decide on α on a case-by-case basis.

Moreover, we do not recommend the TS estimation procedure in (3.7) for robust estimation. Recall from (3.7) that the TS estimators for $\mu_{*}$ and $\sigma_{*}^{2}$ are given by the sample mean and sample variance, respectively, of the $X_{i},i=1,\cdots ,N,$ while the estimators for the thresholds are computed from the empirical cumulative frequencies of the $Y_{i},i=1,\cdots ,N$ . However, if there is contamination in the sample, such as outliers in the data for *X* and careless responses in the data for *Y*, all of these three sample statistics might become heavily biased. This bias is then possibly inherited by the estimate of the correlation coefficient in the second stage. To avoid this problem, our robust estimator estimates all model parameters simultaneously.

## 7 Simulation experiments

This section conducts a number of simulation experiments to evaluate the robustness of the robust minimum DPD estimator against partial misspecification of the polyserial model. Section 7.1 describes the simulation design, Section 7.2 describes the results, and Section 7.3 briefly summarizes additional simulations that are featured in detail in the Supplementary Material.

### 7.1 Design

We consider a polyserial model under which the random vector (X,η) is jointly normally distributed according to (3.2) with true correlation parameter $\rho_{*}=0.5$ and the true first two moments of the observed *X* are $\mu_{*}=E\left[X\right]=0$ as well as $\sigma_{*}^{2}=Var\left[X\right]=1$ . The latent variable η also has zero mean and unit variance and governs the observed ordinal *Y* through the discretization process in (3.1) with true threshold parameters $\tau_{*,1}=-1.5,\tau_{*,2}=-0.5,\tau_{*,3}=0.5,and\tau_{*,4}=1.5,$ so that *Y* has r=5 response categories. Using integer scoring Y={1,2,⋯,5} , the corresponding true point polyserial correlation coefficient in this setting amounts to $\tilde{\rho_{*}}=0.477$ (see Section 3.2).

To simulate contamination, we replace a fraction ε of the data for (X,η) with draws from a particular contamination distribution $H_{X\eta }$ , which is not modeled by our robust estimator nor ever assumed to be known. Specifically, the contamination distribution $H_{X\eta }$ in this simulation is set to a shifted bivariate *t*-distribution with noncentrality parameter ${\left(10,-2\right)}^{⊤}$ , scale matrix diag(0.25,0.25) , and 10 degrees of freedom. To obtain contaminated data for the ordinal *Y*, we discretize this contamination distribution’s realizations in the η -dimension according to the same thresholds $\tau_{*,1},\cdots ,\tau_{*,4}$ as the uncontaminated realizations from the polyserial model. The ensuing contaminated observations are mean-shifted to the right in the continuous *X*-dimension and primarily inflate the first and second response options in the ordinal *Y*-dimension. For instance, the contaminated data in Figure 1 were generated by this process (with contamination fraction ε=0.15 ) and correspondingly exhibit these features. In the robust statistics literature, such contaminating data are an example of *negative leverage points* because they drag positive correlational estimates toward zero or even negative values, thereby creating *negative* leverage.

We sample N=500 observations $(X_{i},Y_{i}),i=1,\cdots ,N$ , from this process with contamination fraction ε∈{0,0.002,0.01,0.05,0.1,0.15,0.2,0.3,0.4,0.49} . Note that ε=0.002 corresponds to only one single contaminated data point for the sample size N=500 . For each simulated data set, we estimate the true parameter $\theta_{*}$ by means of a minimum DPD estimator $\hat{\theta_{N}}$ with tuning constants α∈{0,0.1,0.25,0.5,0.75,1} . This procedure is repeated 5,000 times.

Recall that the tuning constant choice α=0 corresponds to the MLE, for which we use the usual Fisher-information-based asymptotic covariance matrix to compute standard errors instead of the sandwich-type construction in (6.1). Further, recall that larger values of α lead to a more robust estimator, at the cost of a loss in efficiency. In fact, the relative efficiencies in Table 1 were computed for the same parameter value that serves as true value $\theta_{*}$ in this simulation. We do not consider α -values beyond 1 due to their poor efficiency properties of having substantially less than 50% of the MLE’s efficiency (see Table 1). In addition to an estimate $\hat{\rho_{N}}$ of the true polyserial correlation coefficient $\rho_{*}$ , we also construct an estimate $\hat{{\tilde{\rho }}_{N}}$ of the true point polyserial correlation coefficient $\tilde{\rho_{*}}$ from the individual parameter estimates in $\hat{\theta_{N}}$ (see Section 3.2 for details).

Let $\hat{\rho_{N}}$ be a polyserial correlation estimate computed from a given simulated data set, and $\hat{SE}\left(\hat{\rho_{N}}\right)$ be its associated standard error estimate constructed from the limit theory in Theorem A.1 in the Supplementary Material. We evaluate performance by means of the following metrics:

- Bias of the polyserial correlation estimate, $\hat{\rho_{N}}-\rho_{*}$ , and the point polyserial correlation estimate, $\hat{{\tilde{\rho }}_{N}}-\tilde{\rho_{*}}$ , averaged over the 5,000 repetitions.
- Average approximate bias of the standard error estimate, defined as the difference between $\hat{SE}\left(\hat{\rho_{N}}\right)$ and the sample SD of the individual correlation estimates $\hat{\rho_{N}}$ across the 5,000 repetitions, where the latter is a finite-sample approximation of the true population standard error. A rigorous definition is provided in Section C.2 of the Supplementary Material.
- Coverage at the significance level γ=0.05 , defined as the proportion (across repetitions) of confidence intervals $\left[\hat{\rho_{N}}\mp q_{1-\gamma /2}\cdot \hat{SE}\left(\hat{\rho_{N}}\right)\right]$ that contain the true $\rho_{*}$ , where $q_{1-\gamma /2}$ denotes the (1−γ/2) quantile of the standard normal distribution.
- Average confidence interval length at significance level γ=0.05 , where the length of an individual confidence interval is given by $2\cdot q_{1-\gamma /2}\cdot \hat{SE}\left(\hat{\rho_{N}}\right)$ .

### 7.2 Results

**Figure 2:**
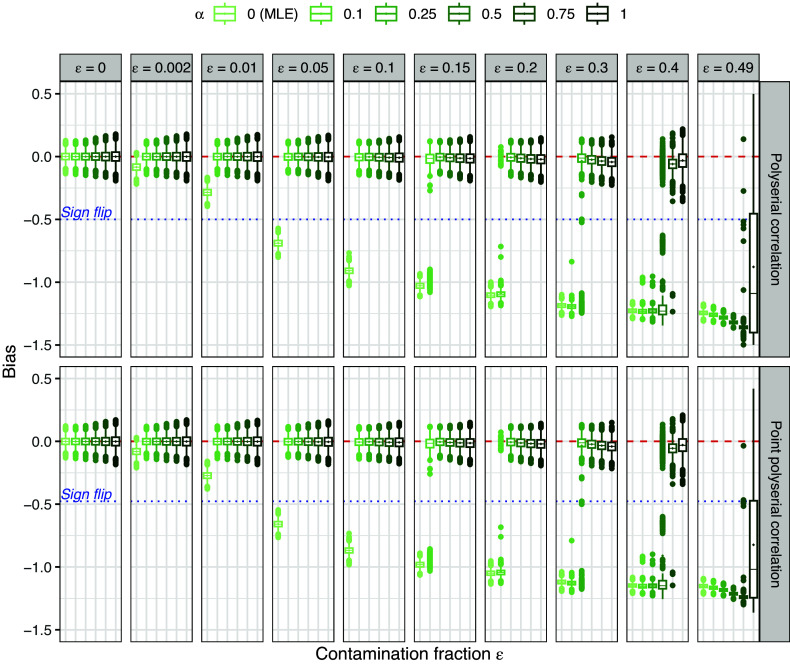
Boxplots of the bias of the considered estimators for the polyserial correlation coefficient, $\hat{\rho_{N}}-\rho_{*}$ (top panel) and the point polyserial correlation coefficient with integer scoring, $\hat{{\tilde{\rho }}_{N}}-\tilde{\rho_{*}}$ (bottom panel), for various contamination fractions in the misspecified polyserial models across 5,000 repetitions. Diamonds represent the respective average bias. The dotted lines at $\rho_{*}=-0.5$ and $-\tilde{\rho_{*}}=-0.477$ indicate a sign flip in the respective estimate. Figure 2 long description.

We start evaluating the results by visualizing the bias of the polyserial and point polyserial estimates. Figure 2 illustrates by means of boxplots the biases across the 5,000 repetitions. An analogous plot for the parameter vector θ is provided in Section C of the Supplementary Material; the results are similar to those of the correlation estimates. Furthermore, as can be immediately seen from Figure 2, the results for polyserial and point polyserial correlation are very much alike, so we restrict the following discussion to polyserial correlation. In particular, Table 2 contains the performance measures for polyserial correlation.

**Table 2:** Performance measures for estimating polyserial correlation coefficients in the simulation in Section 7.1 at significance level γ=0.05 (averaged across 5,000 repetitions) Table 2 long description.

		Point estimate			Standard error		Confidence interval	
Contamination	α	$\hat{\rho_{N}}$	Bias	SD	$\hat{SE}\left(\hat{\rho_{N}}\right)$	Bias	Coverage	Length
ε=0	0 (MLE)	0.500	0.000	0.035	0.036	0.001	0.951	0.142
	0.1	0.500	0.000	0.036	0.036	0.000	0.948	0.141
	0.25	0.499	−0.001	0.037	0.037	0.000	0.949	0.147
	0.5	0.499	−0.001	0.041	0.041	0.000	0.950	0.162
	0.75	0.499	−0.001	0.046	0.046	0.001	0.951	0.182
	1	0.500	0.000	0.051	0.052	0.001	0.953	0.205
ε=0.002	0 (MLE)	0.416	−0.084	0.035	0.039	0.003	0.395	0.152
	0.1	0.499	−0.001	0.036	0.036	0.000	0.948	0.141
	0.25	0.499	−0.001	0.037	0.037	0.000	0.950	0.147
	0.5	0.499	−0.001	0.041	0.041	0.000	0.949	0.162
	0.75	0.499	−0.001	0.046	0.046	0.001	0.951	0.182
	1	0.499	−0.001	0.051	0.052	0.001	0.953	0.205
ε=0.01	0 (MLE)	0.215	−0.285	0.033	0.057	0.024	0.000	0.224
	0.1	0.499	−0.001	0.036	0.036	0.000	0.947	0.142
	0.25	0.499	−0.001	0.037	0.038	0.000	0.950	0.147
	0.5	0.499	−0.001	0.041	0.041	0.001	0.951	0.162
	0.75	0.499	−0.001	0.046	0.046	0.001	0.953	0.182
	1	0.499	−0.001	0.051	0.052	0.001	0.953	0.205
ε=0.05	0 (MLE)	−0.190\phantom0	−0.690	0.032	0.073	0.042	0.000	0.287
	0.1	0.497	−0.003	0.037	0.037	0.000	0.949	0.146
	0.25	0.498	−0.002	0.038	0.038	0.000	0.948	0.150
	0.5	0.497	−0.003	0.041	0.042	0.000	0.947	0.164
	0.75	0.496	−0.004	0.046	0.047	0.001	0.951	0.183
	1	0.496	−0.004	0.051	0.052	0.001	0.953	0.204
ε=0.1	0 (MLE)	−0.409\phantom0	−0.909	0.030	0.063	0.033	0.000	0.248
	0.1	0.494	−0.006	0.039	0.039	0.000	0.948	0.151
	0.25	0.497	−0.003	0.039	0.039	0.000	0.950	0.154
	0.5	0.494	−0.006	0.042	0.043	0.000	0.948	0.167
	0.75	0.492	−0.008	0.046	0.047	0.001	0.950	0.183
	1	0.491	−0.009	0.051	0.052	0.001	0.952	0.202
ε=0.15	0 (MLE)	−0.529\phantom0	−1.029	0.027	0.059	0.032	0.000	0.233
	0.1	0.369	−0.131	0.327	0.042	−0.285\phantom1	0.830	0.164
	0.25	0.495	−0.005	0.040	0.040	0.000	0.948	0.157
	0.5	0.490	−0.010	0.043	0.043	0.000	0.948	0.170
	0.75	0.487	−0.013	0.046	0.047	0.001	0.948	0.184
	1	0.485	−0.015	0.050	0.051	0.001	0.949	0.201
ε=0.2	0 (MLE)	−0.603	−1.103	0.024	0.060	0.035	0.000	0.234
	0.1	−0.591	−1.091	0.078	0.037	−0.041\phantom0	0.004	0.145
	0.25	0.493	−0.007	0.041	0.041	0.000	0.947	0.162
	0.5	0.486	−0.014	0.044	0.044	0.000	0.945	0.172
	0.75	0.481	−0.019	0.047	0.047	0.000	0.941	0.185
	1	0.478	−0.022	0.050	0.051	0.001	0.939	0.199
ε=0.3	0 (MLE)	−0.686	−1.186	0.020	0.063	0.043	0.000	0.247
	0.1	−0.694	−1.194	0.021	0.024	0.003	0.000	0.094
	0.25	0.464	−0.036	0.171	0.043	−0.128\phantom0	0.926	0.170
	0.5	0.475	−0.025	0.045	0.046	0.000	0.923	0.179
	0.75	0.464	−0.036	0.047	0.048	0.001	0.901	0.187
	1	0.456	−0.044	0.049	0.050	0.001	0.882	0.196
ε=0.4	0 (MLE)	−0.727	−1.227	0.018	0.065	0.047	0.000	0.257
	0.1	−0.732	−1.232	0.022	0.019	−0.003\phantom0	0.000	0.073
	0.25	−0.729	−1.229	0.024	0.019	−0.005\phantom0	0.000	0.074
	0.5	−0.475	−0.975	0.500	0.027	−0.473\phantom0	0.191	0.106
	0.75	0.440	−0.060	0.058	0.056	−0.002\phantom0	0.772	0.221
	1	0.468	−0.032	0.074	0.127	0.053	0.818	0.498
ε=0.49	0 (MLE)	−0.745	−1.245	0.017	0.066	0.049	0.000	0.260
	0.1	−0.760	−1.260	0.016	0.016	0.000	0.000	0.064
	0.25	−0.782	−1.282	0.015	0.015	0.000	0.000	0.059
	0.5	−0.820	−1.320	0.014	0.014	0.000	0.000	0.053
	0.75	−0.858	−1.358	0.038	0.031	−0.007\phantom0	0.001	0.118
	1	−0.379	−0.879	0.676	0.122	−0.554\phantom0	0.007	0.478

In the absence of contamination (ε=0) , all estimators yield accurate results, but the robust estimators (those with α>0 ) exhibit more variation than the MLE ( α=0 ), reflecting their diminished efficiency (e.g., Table 1). Furthermore, all estimators attain the nominal coverage level of 95%.

However, the MLE’s performance rapidly deteriorates once contamination is introduced (ε>0) . For instance, one single bad data point ( ε=0.002 here) suffices for the MLE to exhibit a notable bias of about −0.084 , resulting in a deteriorated coverage of less than 40%. At contamination ε=0.01 , its coverage even drops to 0%. At contamination level ε=0.05 , its point estimate experiences a sign flip: instead of yielding a positive estimate of the true value $\rho_{*}=0.5$ , its average estimate of −0.19 is negative. The ML estimate continues to deteriorate until it roughly stabilizes at about ε=0.2 with an average bias of about −0.7 .

Conversely, the minimum DPD estimators ( α>0 ) turn out to be substantially more robust to contamination than ML. Up until and including the contamination fraction of ε=0.1 , all minimum DPD estimators are virtually unaffected and maintain the nominal coverage level. At ε=0.15 , the coverage of the estimator with α=0.1 drops slightly to about 83%. At ε=0.2 , its coverage drops to nearly 0%, while all choices with α>0.1 remain stable with a coverage above 90%. Furthermore, all minimum DPD estimators up to this point accurately estimate the standard error of polyserial correlation (except α=0.1 at ε>0.15 , where its coverage is near 0). In the high-contamination setting of ε=0.3 , estimators with α>0.1 start exhibiting a minor but notable bias, while at ε=0.4 , only the choices α∈{0.75,1} retain a reasonably good performance, while all other estimators have coverage rates of or near zero. At ε=0.49 , the highest considered contamination level, all estimators break down.^11^

This simulation demonstrates that ML estimation of the polyserial correlation coefficient is highly susceptible to already very low levels of contamination. Conversely, using our proposed estimator yields substantial gains in robustness. For instance, at tuning constant α=0.1 , which only sacrifices less than 2% efficiency (Table 1), the robust estimator remains accurate up until a contamination level of about ε=0.1 . Robustness against higher contamination levels can be achieved by choosing higher values of α . In addition, the simulation also exposes the boundaries of the robust estimator: In extremely high-contamination settings of beyond 40% contamination, even the estimator with the highest considered tuning constant value of α=1 breaks down. However, it is questionable whether modeling with data of such extremely poor quality is meaningful to begin with.

### 7.3 Additional simulations

We perform three additional simulation studies, all described in detail in Section D of the Supplementary Material. These simulations are intended to explore the benefits and limitations of our proposed methodology.

The first additional simulation, described in Section D.1 of the Supplementary Material, has the same setup as the design in Section 7.1, but the contamination manifests through extreme outliers in both the *X*- and η -dimension. This simulation is primarily intended to study the behavior of the MLE and robust estimator under gross errors in the data. In brief, one single gross error observation in a sample suffices for the MLE to produce sign-flipped estimates that are extremely biased. In contrast, the robust estimator remains nearly unaffected by small to moderate contamination fractions with such gross errors, and only starts exhibiting considerable biases in high-contamination settings.

The second additional simulation, described in Section D.2 of the Supplementary Material, is motivated by the fact that the simulation design in Section 7.1 is primarily concerned with mean-shifted contamination. Mean-shifted contamination is characterized by the population mean of the contamination distribution $H_{X\eta }$ being markedly different than that of the true normal distribution $P_{X\eta }$ . However, contamination may also manifest through changes in *correlation* rather than means. The second additional simulation therefore focuses on a *correlation-shifted* contamination distribution $H_{X\eta }$ , which is equal to the true normal distribution $P_{X\eta }$ , except for a sign-flipped correlation coefficient $-\rho_{*}$ . In brief, for moderate polyserial correlation $\rho_{*}$ (where $H_{X\eta }$ and $P_{X\eta }$ substantially overlap), our robust estimator does not yield an improvement over ML. Conversely, it does provide a notable gain in robustness for larger true correlations $\rho_{*}$ (where $H_{X\eta }$ and $P_{X\eta }$ barely overlap).

Combined with the simulations on mean-shifted contamination in Section 7.1 and Section D.1 of the Supplementary Material, the results of the simulation on correlation-shifted contamination suggest that the potential for robustness gain depends on the overlap between the contamination distribution $H_{X\eta }$ and true normal distribution $P_{X\eta }$ . If there is much overlap, the robust estimator cannot distinguish well between contamination and regular observations since it does not make any assumptions on the former, so it may not improve upon ML. In contrast, if $H_{X\eta }$ and $P_{X\eta }$ do not overlap much—like with mean-shifted contamination or correlation-shifted contamination with strong true correlation—the robust estimator can identify contaminated observations and subsequently downweigh their influence to achieve considerable robustness gains.

The third additional simulation, described in Section D.3 of the Supplementary Material, is concerned with distributional misspecification, where the polyserial model is misspecified for *all* observations in a sample (cf. Section 4.2). The simulation shows that the potential of robustness gain with our robust estimator depends on the case-specific characteristics of the nonnormal sampling distribution of (X,η) for which the polyserial model is distributionally misspecified. In brief, if the sampling distribution can be reasonably well approximated by a mixture of a normal distribution and some other unknown distribution to emulate the contamination model in (4.1), then the robust estimator can improve upon the MLE. If the sampling distribution does not admit such an approximation, the robust estimator may not yield an improvement but produce similar estimates as ML, which can be quite poor under distributional misspecification (e.g., Bedrick, 1995, and our simulations in Section D.3 of the Supplementary Material). Thus, we advice applied researchers to always test for partially-latent normality when ML and robust estimator yield similar estimates. While such similarity may be due to normality indeed holding true, it may also be due to distributional misspecification from a specific nonnormal distribution for which the robust estimator cannot improve over ML. We provide details and more explicit guidance in Section D.3 of the Supplementary Material.

## 8 Empirical application

To demonstrate our proposed estimator in practice, we apply it to an empirical data set from personality psychology. The data are from an administration of the *Eysenck Personality Inventory* (Eysenck & Eysenck, 1964) to N=231 undergraduate students at Northwestern University (collected by William Revelle) and are publicly available in the R package psychTools (Revelle, 2025). We restrict ourselves to the continuous variable stateanx and the ordinal variable epilie in this demonstration.

**Figure 3:**
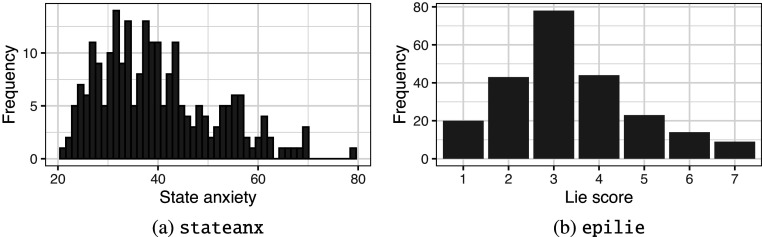
Histogram of the continuous variable stateanx (left) and response frequencies of the ordinal variable epilie (right) in the data of Revelle (2025), for N=231 observations. Figure 3 long description.

The continuous variable stateanx is the score of an unspecified scale measuring *state anxiety*, defined as a temporary emotional reaction to adverse events (e.g., Saviola et al., 2020).^12^ Figure 3a provides a histogram of this variable in the data of Revelle (2025). The empirical distribution seems to be skewed to the right. In particular, there is one unusually large observation in its right tail with value 79 that seems to be separated from the rest of the data, so it may be viewed as an outlier (the sample mean is 39.8). Hence, due to the inflated right tail, the validity of the normality assumption seems questionable. Indeed, the nonparametric Shapiro–Wilk test rejects marginal normality of this variable with a test statistic of W=0.953 and a *p*-value of <0.001 .

The ordinal variable epilie is the score of a lie scale and measures social desirability of a participant’s responses: The higher the score, the less trustworthy their responses. Originally, this variable has 10 unique ordinal response options.^13^ However, in the data of Revelle (2025), no participant has the highest score of 9 or lowest score of 0, and only two participants have the second-highest score of 8. To avoid numerical instability in the estimation of thresholds,^14^ we therefore assign the two participants with score 8 the score 7, that is, we merge the seventh and eighth response categories, and treat the score 1 as the first response category. Thus, the variable epilie here takes values in Y={1,2,⋯,7} with a total of r=7 response options. Figure 3b visualizes the frequency of each response option.

Suppose we are interested in the polyserial correlation coefficient between state anxiety (stateanx) and the ordinal lie score (epilie) in the data of Revelle (2025). We therefore fit the polyserial model to these data using ML and our proposed robust estimator. For the latter, we focus on the results with tuning constant α=0.5 , being the default choice in our software, but also report those with other choices of α .

Table 3 summarizes the results of the two estimators. The correlation estimate is weaker for the robust estimator with a value of −0.107 than for the MLE with a value of −0.142 . The largest differences occur in the moment estimates of the continuous variable stateanx: Both μ and $\sigma^{2}$ are estimated to be notably larger by ML (estimates of about 39.8 and 131.6, respectively) compared to the robust estimator (estimates of about 38 and 107.7, respectively). The threshold estimates are similar. Thus, overall, the robust estimates tend to be weaker in absolute magnitude than those of ML.

**Table 3:** Parameter estimates and standard error estimates ( $\hat{\text{SE}}$ ) for the correlation between the continuous variable stateanx and the ordinal variable epilie (with r=7 response options) in the data of Revelle (2025), using the robust estimator with α=0.5 and the MLE Table 3 long description.

		Robust			ML	
Parameter		Estimate	$\hat{\text{SE}}$		Estimate	$\hat{\text{SE}}$
ρ		−0.107	0.076		−0.142	0.067
μ		37.990	0.615		39.842	0.900
$\sigma^{2}$		107.730	10.280		131.575	14.485
$\tau_{1}$		−1.359	0.120		−1.358	0.118
$\tau_{2}$		−0.586	0.091		−0.604	0.088
$\tau_{3}$		0.276	0.085		0.276	0.084
$\tau_{4}$		0.828	0.096		0.841	0.094
$\tau_{5}$		1.268	0.116		1.286	0.116
$\tau_{6}$		1.849	0.152		1.771	0.152

**Figure 4:**
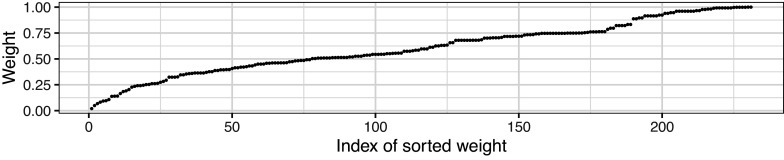
Weights $w_{i,\alpha }\left(\hat{\theta_{N}}\right),i=1,\cdots ,N$ , computed with the robust parameter estimates at tuning constants α=0.5 , using the data in Revelle (2025) for the variables stateanx and epilie. For a clearer visualization, the N=231 weights are sorted here. Figure 4 long description.

To obtain insights as to why the robust estimator’s results differ from those of the MLE, we calculate the individual-specific weights in (5.9) using the robust parameter estimates. Figure 4 visualizes the sorted weights. While many observations can be fitted well with weights of nearly 1, there are also observations that receive a weight of close to 0, indicating that strong downweighting has taken place. We therefore investigate in detail all observations whose weight is reasonably close to zero, say, below 0.1. Table 4 lists the weights and values of the two variables for observations whose estimated weights are below 0.1. The smallest weight (valued 0.02) belongs to the observation with the outlying value of 79 in variable stateanx (Figure 3a). Thus, the robust estimator has identified this observation as outlying and subsequently downweighs it in the estimation procedure. In similar fashion, the other observations in Table 4 are those with relatively large stateanx values. It therefore seems that the robust estimator is downweighting observations whose stateanx values are in the heavy right tail (see Figure 3a). A notable exception is the observation that has a somewhat less high stateanx value of only 61. Nevertheless, this observation’s lie score amounts to the maximum value of 7, indicating low trustworthiness of the given responses. The fact that this observation was strongly downweighted suggests that it could not sufficiently well modeled by the polyserial model, which might be due to its low trustworthiness.

**Table 4:** Weights and values of variables stateanx (continuous) and epilie (ordinal with r=7 ) for observations in the data of Revelle (2025) whose robustly estimated weights are below 0.1 (using tuning constant α=0.5 ) Table 4 long description.

*i*	1	2	3	4	5	6
$w_{i,\alpha }\left(\hat{\theta_{N}}\right)$	0.020	0.049	0.068	0.082	0.093	0.096
stateanx ( $X_{i}$ )	79	69	61	68	70	67
epilie ( $Y_{i}$ )	3	5	7	1	3	4

*Note*: The observations are sorted according to the estimated weights.

**Figure 5:**
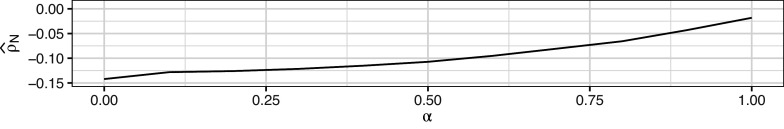
Estimates of the polyserial correlation coefficient for different values of tuning constant α ( α=0 is MLE), computed on the variables stateanx and epilie in the data of Revelle (2025).

As a sensitivity analysis, Figure 5 plots the polyserial correlation estimates computed for various choices of tuning constant α . All strictly positive choices of α yield estimates that are weaker in magnitude than the MLE, but stay around −0.1 up until approximately α=0.6 . Beyond that tuning constant value, the estimates sharply converge to about 0, primarily reflecting more stringent downweighting of the heavy right tail of the continuous variable stateanx. Overall, though, for tuning constants α≤0.6 , we obtain the robust finding of a correlation of about −0.1 , being weaker than the ML estimate of −0.14 .

This empirical application demonstrated the practical usefulness of our proposed robust estimator by showing how it identifies observations that are likely discrepant to the polyserial model and subsequently downweights their influence. It furthermore demonstrates how the estimated weights are an attractive tool for interpretation and exploratory data analysis.

## 9 Discussion and conclusion

Motivated by recent interest in the psychometric literature in the non-robustness of ML estimation of latent variable models under model misspecification, we study estimation of the polyserial correlation model, which models the association between a continuous variable and an ordinal variable. We consider a *partial* misspecification framework stemming from the robust statistics literature where the polyserial model is misspecified for an unknown, possibly zero-valued fraction of an observed sample. Crucially, this framework does not impose any assumptions on *how* the model is possibly misspecified, which might be due to, for instance but not limited to, outliers or careless responses. We show that one single observation not generated by the polyserial model suffices for the commonly used MLE to produce arbitrarily poor results.

As a remedy, we propose a novel estimator designed to be robust against partial misspecification of the polyserial model. The robust estimator leverages the theory of minimum DPD (Basu et al., 1998) estimation, which, as we show, is able to cope with mixed-type variables, unlike standard approaches for robust estimation with exclusively continuous or exclusively categorical variables. The proposed minimum DPD estimator achieves robustness by implicitly downweighting observations that cannot be sufficiently well-fitted by the polyserial model. The ensuing weights are a useful analytical tool for identifying observations discrepant to the polyserial model, such as (but not limited to) outliers and careless responses. Our proposed methodology is implemented as part of the free open-source R package robcat (Welz et al., 2026a), which is publicly available at https://CRAN.R-project.org/package=robcat.

We show that the proposed robust estimator is consistent as well as asymptotically normally distributed, thereby enabling inference. The price to pay for enhanced robustness is diminished statistical efficiency of our estimator compared to ML. However, we show that substantial robustness can be gained with our estimator while maintaining more than 98% of ML efficiency.

We verify the estimator’s robustness and theoretical properties by means of numerous simulation studies. An empirical application on a data set from personality psychology furthermore demonstrates its practical usefulness, where it identifies outlying data points.

A central practical consideration is the choice of tuning constant α , governing the robustness-efficiency tradeoff. While simulations suggest that α=0.5 is a good compromise with about 76% of ML efficiency, we acknowledge that a detailed investigation is an important avenue of further research. We recommend to always compare various choices of α , like in Figure 5, to assess estimation stability and severity of (partial) model misspecification. Alternatively, one may fix the efficiency loss and select the corresponding value of α as part of an ex-ante power analysis (cf. Basu et al., 1998, Section 5).

This article suggests a number of extensions. For instance, while our software implementation is reasonably fast with about 2 seconds for computing a robust estimate, substantial speedups could be achieved by rewriting the source code in C++ via Rcpp (Eddelbuettel, 2013). Moreover, the ability to robustly estimate polyserial correlation could be particularly useful in structural equation modeling with mixed data to robustify such analyses against partial model misspecification. We leave these avenues to further research. Overall, we believe that our robust estimator can contribute to the growing literature on making latent variable analyses less dependent on easily violated modeling assumptions.

## Supporting information

10.1017/psy.2026.10091.sm001 Welz supplementary material Welz supplementary material

## Data Availability

Replication files are publicly available on GitHub at https://github.com/mwelz/robust-polyserial-replication.

## Footnotes

1 It should be noted that ML estimation of polychoric correlation was originally believed to be fairly robust against distributional misspecification of latent normality (e.g., Coenders et al., 1997; Flora & Curran, 2004; Li, 2016), an assessment based on simulation experiments using the method of Vale and Maurelli (1983) for generating nonnormal bivariate data that are then discretized to be ordinal. However, Grønneberg and Foldnes (2019) show that ordinal data generated in this way are indistinguishable from ordinal data generated by latent normality. Using a method that ensures proper violation of latent normality (the VITA method of Grønneberg & Foldnes, 2017) reveals strong susceptibility of ML estimation of polychoric correlation to such violations (e.g., Foldnes & Grønneberg, 2020, 2022).

2 Being a random vector comprising a continuous and discrete variable, (X,Y) technically does not have a joint density in a regular sense, that is, a Randon–Nikodým derivative with respect to the two-dimensional Lebesgue measure. The formally correct way to refer to $p_{XY}$ in (3.3) would be that it is the Randon–Nikodým derivative of the distribution $P_{XY}$ in (3.4) with respect to the product of the one-dimensional Lebesgue measure and the counting measure. For the sake of brevity, however, we refer to $p_{XY}$ simply as joint density in this article, acknowledging an abuse of terminology.

3 Being a population quantity and not a sample quantity, it is strictly speaking imprecise to call ε a fraction. It would be more accurate to call ε a (contamination) population probability with which one samples from the contamination distribution $H_{X\eta }$ instead of the model distribution $P_{X\eta }$ . However, to be consistent with robust statistics literature, we refer to ε as a fraction throughout this article.

4 To explicitly accommodate discrete distributions in the definition of the KL divergence, we could have adapted the Riemann-integral-based definition in (5.1) to a definition based on Lebesgue integration with respect to a general measure that can either be the Lebesgue measure (for continuous distributions) or the counting measure (for discrete distributions). However, we believe that is more instructive to refrain from introducing measure-theoretic concepts in this article.

5 Alternatively, the MLE (and also the proposed robust estimator) can be equivalently defined using statistical divergence functionals, as done in Basu et al. (1998).

6 By elementary likelihood theory, c0(θ)=∫R∑y∈Yp^XYx,y;θuθx,ydx=EPXYuθX,Y=0 for all θ∈Θ .

7 The original quote of Basu et al. (1998, p. 551) refers to contaminated observations as *outlying* observations. In their terminology, an outlier is any data point that was not generated by the postulated model. However, since one often associates outliers with extreme values rather than discordant model fit, we believe that the term “outlying” is potentially misleading, so we prefer “contaminated.”

8 We stress that this sentence is a heuristic rather than a formal result. Since no assumption is made on the contamination distribution $H_{X\eta }$ , there may exist contaminated distributions $F_{\varepsilon ,X\eta }$ for which higher values of α do *not* uniformly reduce bias. We refer to Hampel et al. (1986, Chapter 8.2d) for a discussion on the fundamental difficulty of deriving bias-reduction results of robust procedures in contamination models such as (4.1).

9 In contrast, for exclusively categorical variables, it is possible to construct robust estimators that are fully efficient. See Welz (2024) for general models of categorical data, and Welz et al. (2026b) for the special case of the polychoric correlation model.

10 In our experience, the higher the contamination fraction, the more likely it is that unconstrained optimization fails, so constrained algorithms must be used instead. As an example, we refer to the convergence statistics for the simulation in Section 7, reported in Section C of the Supplementary Material.

11 All point estimators always numerically converged across all configurations. However, in extremely high-contamination settings, the covariance matrix estimator could occasionally not be computed due to singularity issues. Such non-existent standard error estimates were omitted in Figure 2 and Table 2. Nonconvergence statistics are provided in Section C of the Supplementary Material.

12 Technically, being an aggregate score of ordinal measurements, the variable stateanx is strictly speaking not continuous. However, due to the granularity of the measurements (see Figure 3a), it may be viewed as being quasi-continuous.

13 The Eysenck Personality Inventory constructs the variable epilie as the number of times that a given participant has responded “yes” in nine separate yes–no questions. Since higher scores are associated with higher levels of socially desirable responding, this variable can be interpreted as being ordinal.

14 It may happen that a robust estimator attempts to effectively eliminate thresholds corresponding to (nearly) empty cells in a certain way that we describe in detail in Section D.3.2 of the Supplementary Material, thereby causing instability. This phenomenon has been noted before by Welz et al. (2026b).

## Supplementary material

The supplementary material for this article can be found at https://doi.org/10.1017/psy.2026.10091.

## Figure 1 Long description

The horizontal axis is labeled X, with a dashed vertical line at X equals zero marked mu equals zero. The vertical axis is labeled eta. Gray points, representing the polyserial model, are densely clustered left of X equals zero, spanning eta from about minus four to four. Orange points, representing contamination, form a tight cluster centered near X equals ten and eta equals zero. Five horizontal blue lines segment the eta axis into five regions, each labeled from bottom to top as Y equals one with probability zero point zero six seven, Y equals two with probability zero point two four two, Y equals three with probability zero point three eight three, Y equals four with probability zero point two four two, and Y equals five with probability zero point zero six seven. The legend at the right identifies gray as polyserial model and orange as contamination.

Navigate back to Figure 1.

## Table 1 Long description

The table has one header row and one data row. The header row lists alpha at the far left, followed by columns labeled 0 (M L E), 0.1, 0.25, 0.5, 0.75, and 1. The data row, labeled rel. efficiency, contains values: 1.000 under 0 (M L E), 0.983 under 0.1, 0.916 under 0.25, 0.762 under 0.5, 0.612 under 0.75, and 0.488 under 1. The table summarizes how relative efficiency for estimating the polyserial correlation coefficient decreases as alpha increases, with the highest efficiency at alpha equals zero and the lowest at alpha equals one. The context specifies Y has five response options and the true parameter vector is rho sub star equals 0.5, mu sub star equals 0, sigma sub star squared equals 1, tau sub star 1 equals minus 1.5, tau sub star 2 equals minus 0.5, tau sub star 3 equals 0.5, tau sub star 4 equals 1.5.

Navigate back to Table 1.

## Figure 2 Long description

The chart is organized into two horizontal panels. The top panel is labeled Polyserial correlation and the bottom panel is labeled Point polyserial correlation. Each panel contains ten vertical grids, each corresponding to a contamination fraction epsilon, increasing from left to right: 0, 0.002, 0.01, 0.05, 0.1, 0.15, 0.2, 0.3, 0.4, and 0.49. The x-axis is labeled Contamination fraction epsilon and the y-axis is labeled Bias, ranging from -1.5 to 0.5. Within each grid, seven boxplots are shown for alpha values 0 (M L E), 0.1, 0.25, 0.5, 0.75, and 1, with lighter to darker green shades. Diamonds indicate the average bias for each estimator. In both panels, a horizontal red dashed line marks the zero bias level. In the top panel, a blue dotted line at bias equals negative 0.5 is labeled Sign flip, and in the bottom panel, a blue dotted line at bias equals negative 0.477 is labeled Sign flip. As contamination fraction increases, the spread and downward shift of the boxplots become more pronounced, especially for lower alpha values, indicating increased negative bias and estimator instability. The sign flip lines highlight where estimator bias crosses critical thresholds.

Navigate back to Figure 2.

## Table 2 Long description

The table is organized with contamination level epsilon as the primary row anchor, with values 0, 0.002, 0.01, 0.05, 0.1, 0.15, 0.2, 0.3, 0.4, and 0.49. For each epsilon, sub-rows are defined by alpha values: 0 (MLE), 0.1, 0.25, 0.5, 0.75, and 1. Columns are grouped into three main sections: point estimate (with columns for estimated rho sub N, bias, and SD), standard error (estimated SE of rho sub N and bias), and confidence interval (coverage and length). For epsilon equals 0, all alpha values yield estimated rho sub N near 0.5, bias near zero, SD and estimated SE near 0.035 to 0.051, coverage near 0.95, and interval length from 0.142 to 0.205. As epsilon increases, for alpha equals 0 (MLE), estimated rho sub N decreases sharply, bias becomes strongly negative, SD decreases, estimated SE increases, coverage drops to zero, and interval length increases. For alpha greater than zero, estimated rho sub N remains near 0.5, bias stays near zero, SD and estimated SE remain stable, coverage stays near 0.95, and interval length increases moderately. At high contamination (epsilon equals 0.49), alpha equals 0 (MLE) yields estimated rho sub N of negative 0.745, bias negative 1.245, SD 0.017, estimated SE 0.066, bias 0.049, coverage zero, and interval length 0.260. For alpha equals 1, estimated rho sub N is negative 0.379, bias negative 0.879, SD 0.676, estimated SE 0.122, bias negative 0.554, coverage 0.007, and interval length 0.478. The table demonstrates that robust estimators (alpha greater than zero) maintain accuracy and coverage under contamination, while MLE performance degrades rapidly.

Navigate back to Table 2.

## Table 3 Long description

The table presents parameter estimates and standard errors for the correlation between the continuous variable stateanx and the ordinal variable epilie, with seven response options, using both the robust estimator with alpha equals zero point five and maximum likelihood estimation. Columns from left to right are: parameter, robust estimate, robust standard error, M L estimate, and M L standard error. Parameters listed from top to bottom are rho, mu, sigma squared, tau sub 1, tau sub 2, tau sub 3, tau sub 4, tau sub 5, and tau sub 6. For rho, robust estimate is negative zero point one zero seven with standard error zero point zero seven six; M L estimate is negative zero point one four two with standard error zero point zero six seven. For mu, robust estimate is thirty seven point nine nine zero with standard error zero point six one five; M L estimate is thirty nine point eight four two with standard error zero point nine zero zero. For sigma squared, robust estimate is one hundred seven point seven three zero with standard error ten point two eight zero; M L estimate is one hundred thirty one point five seven five with standard error fourteen point four eight five. For tau sub 1, robust estimate is negative one point three five nine with standard error zero point one two zero; M L estimate is negative one point three five eight with standard error zero point one one eight. For tau sub 2, robust estimate is negative zero point five eight six with standard error zero point zero nine one; M L estimate is negative zero point six zero four with standard error zero point zero eight eight. For tau sub 3, both robust and M L estimates are zero point two seven six with standard errors zero point zero eight five and zero point zero eight four, respectively. For tau sub 4, robust estimate is zero point eight two eight with standard error zero point zero nine six; M L estimate is zero point eight four one with standard error zero point zero nine four. For tau sub 5, robust estimate is one point two six eight with standard error zero point one one six; M L estimate is one point two eight six with standard error zero point one one six. For tau sub 6, robust estimate is one point eight four nine with standard error zero point one five two; M L estimate is one point seven seven one with standard error zero point one five two.

Navigate back to Table 3.

## Table 4 Long description

The table contains three rows and six columns of data, with columns labeled 1 through 6. The first row lists robustly estimated weights w sub i comma alpha open parenthesis theta hat sub N close parenthesis: 0.020, 0.049, 0.068, 0.082, 0.093, 0.096. The second row lists stateanx values X sub i: 79, 69, 61, 68, 70, 67. The third row lists epilie values Y sub i: 3, 5, 7, 1, 3, 4. All observations are sorted in ascending order by weight, and each weight is less than 0.1. The tuning constant alpha equals 0.5 and the ordinal variable epilie has r equals 7 levels.

Navigate back to Table 4.

## Figure 3 Long description

In the left panel, the x axis is labeled State anxiety and ranges from twenty to eighty. The y axis is labeled Frequency and ranges from zero to fifteen. The histogram shows a multimodal distribution with peaks around thirty and forty, and lower frequencies above sixty. In the right panel, the x axis is labeled Lie score and ranges from one to seven. The y axis is labeled Frequency and ranges from zero to eighty. The bar chart shows the highest frequency at score three, moderate frequencies at scores two and four, and decreasing frequencies from five to seven. Panel labels are a stateanx and b epilie.

Navigate back to Figure 3.

## Figure 4 Long description

The x axis is labeled Index of sorted weight, ranging from zero to two hundred thirty. The y axis is labeled Weight, spanning zero to one. Each point represents a weight w sub i comma alpha, calculated using robust parameter estimates at alpha equals zero point five for stateanx and epilie variables from Revelle two thousand twenty five. The points form an upward trend, starting near zero at the leftmost index and increasing gradually to reach one at the rightmost index. The distribution is nearly linear but shows small plateaus and steps, indicating clusters of similar weights. No outliers or abrupt jumps are visible.

Navigate back to Figure 4.
