# Supplementary material for: Robust Estimation of Polyserial Correlation Coefficients: A Density Power Divergence Approach
Source: Psychometrika. 2026 Feb 10;91(2):445–72. doi: 10.1017/psy.2026.10091 (PMC13310821; doi:10.1017/psy.2026.10091)
Supplement: Welz supplementary material [file S003331232610091Xsup001.pdf]

*Psychometrika* (2026), **xx**:xx 1–31

CAMBRIDGE
UNIVERSITY PRESS

ONLINE SUPPLEMENT

# Online Supplementary Materials to "Robust estimation of polyserial correlation coefficients: A density power divergence approach"

Max Welz[1]

[1]Department of Psychology, University of Zurich, Zurich, CH-8050, Switzerland. E-mail: max.welz@uzh.ch.

**Abstract**

This online supplement to the paper *"Robust estimation of polyserial correlation coefficients: A density power divergence approach"* (Welz, 2026) contains four sections. Section A provides technical details on the polyserial model and robust estimation thereof. Section B describes an algorithm for the rescaling of outlyingness weights. Section C contains additional results from the main text. Section D provides additional simulation studies. Unless stated otherwise, all references to labels that start with an Arabic numeral, such as (1.1), refer to labels in the main paper. Conversely, all references to labels starting with a Roman letter, such as (A.1), refer to labels in this online supplement.

## A. Technical details

This appendix section contains technical details on the polyserial model and robust estimation thereof. Concerning the polyserial model, Subsection A.1 covers identification of point polyserial correlation and Subsection A.2 provides alternative but equivalent definitions of the polyserial model density. Concerning estimation, Subsection A.3 introduces compact notation for the asymptotic analysis, Subsection A.4 rigorously establishes the main theorem, Subsection A.5 compares the asymptotic covariance matrices of ML to the robust estimator, Subsection A.6 discusses covariance matrix estimation, and Subsection A.7 provides closed-form gradient and Hessian expressions.

### *A.1. Identification of point polyserial correlation*

Point polyserial correlation is the correlation between the observed variable $X$ and the observed ordinal variable $Y$ under the partially-latent normality assumption. To identify point polyserial correlation, one needs to introduce a scoring system for the response options of the ordinal $Y$. Recall that we assume that $Y$ takes values in the set $\mathcal{Y} = \{1, 2, \dots, r\}$, where the elements in $\mathcal{Y}$ need not admit a numerical interpretation. Assigning a scoring system introduces a numeric meaning to each response category. We refer to Fernández et al. (2020), Ivanova and Berger (2001), and Olsson et al. (1982, pp. 340–341) for discussions of different scoring systems.

We temporarily drop the assumption that the response set of $Y$ is given by adjacent integers $1, 2, \dots, r$, and instead assume that $\mathcal{Y} = \{y_1, y_2, \dots, y_r\}$, where the response options $-\infty < y_1 < y_2 < \cdots < y_r < +\infty$ are real-valued *and* admit a numerical interpretation. As such, there is a particular scoring system associated with the response options. Then, under the polyserial model at a parameter vector $\boldsymbol{\theta}$, the marginal probability of the $j$-th response option, $y_j$, is given by

$$p_Y\left(y_j; \boldsymbol{\theta}\right) = \Phi\left(\tau_j\right) - \Phi\left(\tau_{j-1}\right), \qquad j = 1, \dots, r,$$

so the population mean $\mu_Y$ and variance $\sigma_Y^2$ of $Y$ are respectively identified through

$$\begin{aligned} \mu_Y &= \mathbb{E}\left[Y\right] = \sum_{j=1}^{r} y_j \cdot p_Y\left(y_j; \boldsymbol{\theta}\right), \qquad \text{and} \\ \sigma_Y^2 &= \mathbb{V}\text{ar}\left[Y\right] = \sum_{j=1}^{r} y_j^2 \cdot p_Y\left(y_j; \boldsymbol{\theta}\right) - \mu_Y^2. \end{aligned} \tag{A.1}$$

To clearly distinguish between the population moments of $X$ and $Y$, we write $\mu_X = \mu$ and $\sigma_X^2 = \sigma^2$ whenever there is a risk of ambiguity. It can be shown that the product moment of the two observed variables is equal to

$$\mu_{XY} = \mathbb{E}\left[XY\right] = \mu_X \left( y_r - \sum_{j=1}^{r-1} \Phi\left(\tau_j\right)\left(y_{j+1} - y_j\right) \right) + \rho\sigma_X \sum_{j=1}^{r-1} \phi\left(\tau_j\right)\left(y_{j+1} - y_j\right), \tag{A.2}$$

where $\rho$ denotes the polyserial correlation coefficient from the normality model in (3.2) and $\phi(\cdot)$ denotes the univariate standard normal density function. It follows from the identity $\mathbb{C}\text{ov}\left[X,\ Y\right] = \mu_{XY} - \mu_X\mu_Y$

that the population correlation between $X$ and $Y$ can be written as

$$\widetilde{\rho} = \mathbb{C}\text{or}\,[X,\ Y] = \frac{\mathbb{C}\text{ov}\,[X,\ Y]}{\sigma_X \sigma_Y} = \frac{1}{\sigma_X \sigma_Y}\left(\mu_{XY} - \mu_X \mu_Y\right), \tag{A.3}$$

where explicit expressions for $(\mu_Y, \sigma_Y^2)$ and $\mu_{XY}$ are provided in Equations (A.1) and (A.2), respectively. The correlation coefficient $\widetilde{\rho}$ between the continuous $X$ and the ordinal $Y$ is referred to as *point polyserial coefficient*. For a given scoring system, $\widetilde{\rho}$ can be computed from the parameter vector $\boldsymbol{\theta} = \left(\rho, \mu, \sigma^2, \boldsymbol{\tau}^\top\right)^\top$ of the polyserial model, a property established by Olsson et al. (1982). In particular, an estimator of $\widetilde{\rho}$ can be constructed by substituting $\boldsymbol{\theta}$ for an estimate thereof in Equations (A.1)–(A.3).

For the remainder of this appendix, we resort back to the original assumption that the support of the ordinal $Y$ is given by a set of adjacent integers without numeric interpretation, $\mathcal{Y} = \{1, 2, \ldots, r\}$, which, we reiterate, is without loss of generality for identifying the polyserial correlation coefficient.

### *A.2. Alternative expressions of the polyserial model density*

The expressions of the polyserial model's density given in this paper in (3.3) are different but equivalent to the expression used in Olsson et al. (1982). For completeness, we provide the expressions of Olsson et al. (1982) in this subsection.

Under the polyserial model, the joint density of observing a realization $x \in \mathbb{R}$ of $X$ and a response $y \in \mathcal{Y} = \{1, \ldots, r\}$ of the ordinal $Y$ at a parameter vector $\boldsymbol{\theta} \in \boldsymbol{\Theta}$ can decomposed as

$$p_{XY}(x, y; \boldsymbol{\theta}) = p_X(x; \boldsymbol{\theta})\, p_{Y|X}(y \mid x; \boldsymbol{\theta}), \tag{A.4}$$

where the right-hand side follows by Bayes' theorem. The marginal density $p_X(x; \boldsymbol{\theta})$ and the conditional density $p_{Y|X}(y \mid x; \boldsymbol{\theta})$ are implied by the bivariate normality model (3.2) and are defined as follows.

For the marginal density of $X$ in (A.4), we have

$$p_X(x; \boldsymbol{\theta}) = \frac{1}{\sqrt{2\pi\sigma^2}} \exp\left(-\frac{1}{2\sigma^2}(x - \mu)^2\right), \qquad x \in \mathbb{R},$$

which is the univariate normal density with mean $\mu$ and variance $\sigma^2$. This density only depends on these two parameters, so it holds true that $p_X(x; \boldsymbol{\theta}) = p_X\left(x; \mu, \sigma^2\right)$.

For the conditional density $Y|X$ in (A.4), we have

$$p_{Y|X}(y \mid x; \boldsymbol{\theta}) = \Phi\left(\tau_y^*(x; \boldsymbol{\theta})\right) - \Phi\left(\tau_{y-1}^*(x; \boldsymbol{\theta})\right), \qquad y \in \mathcal{Y},\ x \in \mathbb{R},$$

where $\Phi(\cdot)$ denotes the univariate standard normal cumulative distribution function, and

$$\tau_y^*(x; \boldsymbol{\theta}) = \frac{\tau_y - \rho(x - \mu)/\sigma}{\sqrt{1 - \rho^2}}.$$

Finally, it follows from the density $p_{XY}(x, y; \boldsymbol{\theta})$ in (A.4) that the polyserial model distribution function in (3.4) can also be expressed as

$$P_{XY}(x, y; \boldsymbol{\theta}) = \int_{-\infty}^{x} \sum_{\{w \in \mathcal{Y} : w \le y\}} p_X(u; \boldsymbol{\theta})\, p_{Y|X}(w \mid u; \boldsymbol{\theta})\, \mathrm{d}u,$$

for $x \in \mathbb{R}$, $y \in \mathcal{Y}$, and parameter vector $\boldsymbol{\theta} \in \boldsymbol{\Theta}$.

### *A.3. Notation*

To simplify notation, we introduce the following compact notation. First, write $z = (x, y)^\top$ to collect continuous-ordinal pairs $(x, y) \in \mathbb{R} \times \mathcal{Y}$, and, analogously, write $\boldsymbol{Z} = (X, Y)^\top$ for the corresponding random variables. Thus, the polyserial model density $p_{XY}(x, y; \boldsymbol{\theta})$ now reads $p_{\boldsymbol{Z}}(z; \boldsymbol{\theta})$. Throughout this section, assume that one has access to a random sample of observations $\boldsymbol{Z}_i = (X_i, Y_i)^\top, i = 1, \ldots, N$, that are distributed according to the unknown sampling distribution $F_{\varepsilon,XY}$ in (4.2).

Furthermore, in a slight abuse of integral notation, for some possibly vector-valued function $\psi(z; \boldsymbol{\theta})$ of $z \in \mathbb{R} \times \mathcal{Y} = \mathcal{Z}$ and $\boldsymbol{\theta} \in \boldsymbol{\theta}$, we define the shorthand

$$\int_{\mathcal{Z}} \psi(z; \boldsymbol{\theta}) p_{\boldsymbol{Z}}(z; \boldsymbol{\theta})\, \mathrm{d}z = \int_{\mathbb{R}} \sum_{y \in \mathcal{Y}} \psi(x, y; \boldsymbol{\theta}) p_{XY}(x, y; \boldsymbol{\theta})\, \mathrm{d}x.$$

In addition, let

$$\nabla_{\boldsymbol{\theta}} \psi(z; \boldsymbol{\theta}) = \frac{\partial \psi(z; \boldsymbol{\theta})}{\partial \boldsymbol{\theta}} \qquad \text{and} \qquad \nabla^2_{\boldsymbol{\theta}} \psi(z; \boldsymbol{\theta}) = \frac{\partial^2 \psi(z; \boldsymbol{\theta})}{\partial \boldsymbol{\theta} \partial \boldsymbol{\theta}^\top}$$

respectively denote the gradient and Hessian with respect to $\boldsymbol{\theta}$ of $\psi(z; \boldsymbol{\theta})$.

The Fisher information matrix of the polyserial model at a parameter vector $\boldsymbol{\theta} \in \boldsymbol{\Theta}$ is defined as

$$\boldsymbol{I}_{\boldsymbol{\theta}} = \int_{\mathcal{Z}} p_{\boldsymbol{Z}}(z; \boldsymbol{\theta})\, \boldsymbol{u}_{\boldsymbol{\theta}}(z)\, \boldsymbol{u}_{\boldsymbol{\theta}}(z)^\top\, \mathrm{d}z, \tag{A.5}$$

where the log-likelihood score function is given by

$$\begin{aligned} \boldsymbol{u}_{\boldsymbol{\theta}}(z) &= \nabla_{\boldsymbol{\theta}} \log p_{\boldsymbol{Z}}(z; \boldsymbol{\theta}) \\ &= \frac{1}{p_{\boldsymbol{Z}}(z; \boldsymbol{\theta})} \nabla_{\boldsymbol{\theta}} p_{\boldsymbol{Z}}(z; \boldsymbol{\theta}) \\ &= \frac{1}{p_{Y|X}(y \mid x; \boldsymbol{\theta})} \nabla_{\boldsymbol{\theta}} p_{Y|X}(y \mid x; \boldsymbol{\theta}) + \frac{1}{p_X(x; \boldsymbol{\theta})} \nabla_{\boldsymbol{\theta}} p_X(x; \boldsymbol{\theta}), \end{aligned}$$

where the third equality follows from (A.4) and the chain rule. Alternatively, under the polyserial model, the Fisher information can be equivalently defined as

$$\boldsymbol{I}_{\boldsymbol{\theta}} = \int_{\mathcal{Z}} p_{\boldsymbol{Z}}(z; \boldsymbol{\theta})\, \boldsymbol{Q}_{\boldsymbol{\theta}}(z)\, \mathrm{d}z,$$

where

$$\begin{aligned} \boldsymbol{Q}_{\boldsymbol{\theta}}(z) &= -\nabla^2_{\boldsymbol{\theta}} \log p_{\boldsymbol{Z}}(z; \boldsymbol{\theta}) \\ &= -\Bigg\{ \frac{1}{p_{Y|X}(y \mid x; \boldsymbol{\theta})} \nabla^2_{\boldsymbol{\theta}} p_{Y|X}(y \mid x; \boldsymbol{\theta}) - \frac{1}{p^2_{Y|X}(y \mid x; \boldsymbol{\theta})} \nabla_{\boldsymbol{\theta}} p_{Y|X}(y \mid x; \boldsymbol{\theta})\, \nabla^\top_{\boldsymbol{\theta}} p_{Y|X}(y \mid x; \boldsymbol{\theta}) + \\ &\qquad \frac{1}{p_X(x; \boldsymbol{\theta})} \nabla^2_{\boldsymbol{\theta}} p_X(x; \boldsymbol{\theta}) - \frac{1}{p^2_X(x; \boldsymbol{\theta})} \nabla_{\boldsymbol{\theta}} p_X(x; \boldsymbol{\theta})\, \nabla^\top_{\boldsymbol{\theta}} p_X(x; \boldsymbol{\theta}) \Bigg\} \end{aligned}$$

denotes the negative Hessian matrix of the log-likelihood, where the second equality follows by (A.4) in conjunction with the chain rule and product rule. We provide closed-form expressions of the gradient and Hessian of the densities $p_{Y|X}(y \mid x; \boldsymbol{\theta})$ and $p_X(x; \boldsymbol{\theta})$ in Appendix A.7.

### *A.4. Main theorem*

We start by introducing certain regularity conditions under which Basu et al. (1998) establish consistency for the estimand $\boldsymbol{\theta}_0$ as well as asymptotic normality of minimum DPD estimators. These regularity assumptions are stated in Assumption A.1 and are adapted to the polyserial model. We omit assumptions that are automatically satisfied for the polyserial model, such as certain differentiability conditions on the postulated model. We refer to Basu et al. (1998) for a complete presentation of the assumptions for minimum DPD estimation.

**Assumption A.1.** For any given tuning constant $\alpha \geq 0$, impose the following regularity conditions.

A.1 The distribution $H_{XY}$ in (4.2) has the same support as the polyserial model distribution $P_{XY}$, that is, $\mathbb{R} \times \mathcal{Y}$.
A.2 The estimand $\boldsymbol{\theta}_0$, being defined as the minimizer of the population DPD $\mathrm{D}_\alpha\left(f_{\varepsilon,\boldsymbol{Z}} \,\|\, p_{\boldsymbol{Z}}(\cdot,\cdot;\boldsymbol{\theta})\right)$, is a global minimum, unique, and an interior point of the parameter space $\boldsymbol{\Theta}$.
A.3 The $d \times d$ population matrix $\boldsymbol{J}(\boldsymbol{\theta}), \boldsymbol{\theta} \in \boldsymbol{\Theta}$, defined by

$$\begin{aligned}\boldsymbol{J}(\boldsymbol{\theta}) = &\int_{\mathcal{Z}} p_{\boldsymbol{Z}}^{1+\alpha}(z;\boldsymbol{\theta})\,\boldsymbol{u}_{\boldsymbol{\theta}}(z)\,\boldsymbol{u}_{\boldsymbol{\theta}}(z)^\top \mathrm{d}z\, + \\ &\int_{\mathcal{Z}} p_{\boldsymbol{Z}}^{\alpha}(z;\boldsymbol{\theta})\left(\boldsymbol{Q}_{\boldsymbol{\theta}}(z) - \alpha\boldsymbol{u}_{\boldsymbol{\theta}}(z)\,\boldsymbol{u}_{\boldsymbol{\theta}}(z)^\top\right)\Big(f_{\varepsilon,\boldsymbol{Z}}(z) - p_{\boldsymbol{Z}}(z;\boldsymbol{\theta})\Big)\mathrm{d}z,\end{aligned} \tag{A.6}$$

is positive definite in an open neighborhood of the estimand $\boldsymbol{\theta}_0$.
A.4 In an open neighborhood of $\boldsymbol{\theta}_0$, all third-order partial derivatives (with respect to the parameters) of the population DPD $\mathrm{D}_\alpha\left(f_{\varepsilon,\boldsymbol{Z}} \,\|\, p_{\boldsymbol{Z}}(\cdot,\cdot;\boldsymbol{\theta})\right)$ exist and are finite.

Assumption A.1 requires that the second argument of the distribution $H_{XY}$ takes values in $\mathcal{Y}$ and that its first argument is real-valued. Such an assumption is natural for compatibility of the two distributions, and ensures well-definedness of the sampling distribution $F_{\varepsilon,XY}$ in (4.2) Assumption A.2 ensures that the estimand $\boldsymbol{\theta}_0$ is point-identified by the sampling distribution $F_{\varepsilon,XY}$. Assumption A.3 is necessary for well-definedness of the estimator's asymptotic covariance matrix because the latter requires invertibility of $\boldsymbol{J}(\boldsymbol{\theta}_0)$, as we shall see. In similar fashion, Assumption A.4 is also required for existence and finiteness of the asymptotic covariance matrix.

The following theorem establishes the asymptotic properties of our proposed estimator. It follows immediately by Theorem 2 in Basu et al. (1998).

**Theorem A.1.** *Grant Assumption A.1. For fixed $\alpha \geq 0$, it holds true that*

$$\widehat{\boldsymbol{\theta}}_N \xrightarrow{\mathbb{P}} \boldsymbol{\theta}_0$$

*as well as*

$$\sqrt{N}\left(\widehat{\boldsymbol{\theta}}_N - \boldsymbol{\theta}_0\right) \xrightarrow{\mathrm{d}} \mathrm{N}_d\Big(\boldsymbol{0}, \boldsymbol{\Sigma}(\boldsymbol{\theta}_0)\Big),$$

*as $N \to \infty$, where*

$$\boldsymbol{\Sigma}(\boldsymbol{\theta}_0) = \boldsymbol{J}(\boldsymbol{\theta}_0)^{-1} \boldsymbol{K}(\boldsymbol{\theta}_0) \boldsymbol{J}(\boldsymbol{\theta}_0)^{-1}.$$

*The $d \times d$ matrices $\boldsymbol{J}(\boldsymbol{\theta}_0)$ and $\boldsymbol{K}(\boldsymbol{\theta}_0)$ are respectively defined in (A.6) and*

$$\boldsymbol{K}(\boldsymbol{\theta}) = \int_{\mathcal{Z}} f_{\varepsilon,\boldsymbol{Z}}(\boldsymbol{z}) p_{\boldsymbol{Z}}^{2\alpha}(\boldsymbol{z};\boldsymbol{\theta}) \boldsymbol{u}_{\boldsymbol{\theta}}(\boldsymbol{z}) \boldsymbol{u}_{\boldsymbol{\theta}}(\boldsymbol{z})^\top \mathrm{d}\boldsymbol{z} - \boldsymbol{\xi}(\boldsymbol{\theta}) \boldsymbol{\xi}(\boldsymbol{\theta})^\top, \qquad \boldsymbol{\theta} \in \boldsymbol{\Theta},$$

*where*

$$\boldsymbol{\xi}(\boldsymbol{\theta}) = \int_{\mathcal{Z}} f_{\varepsilon,\boldsymbol{Z}}(\boldsymbol{z}) p_{\boldsymbol{Z}}^{\alpha}(\boldsymbol{z};\boldsymbol{\theta}) \boldsymbol{u}_{\boldsymbol{\theta}}(\boldsymbol{z}) \mathrm{d}\boldsymbol{z}$$

*is a $d$-vector, and both matrices are positive definite at $\boldsymbol{\theta}_0$.*

It is worth mentioning that the proof of Basu et al. (1998) follows a classic argument for establishing asymptotic normality, namely the proof of Theorem 3.10 in Lehmann and Casella (1998), but adapts it to the more general case of minimum DPD estimation because the proof of Lehmann and Casella (1998) is restricted to ML estimation.

Being a population object, the asymptotic covariance matrix $\boldsymbol{\Sigma}(\boldsymbol{\theta}_0)$ is unobserved in practice. We describe in Appendix A.6 how it can be estimated in practice. Before that, though, we describe in the following section how it relates to the asymptotic covariance matrix of the MLE.

### *A.5. Uncertainty quantification: Comparison with ML*

The matrix $\boldsymbol{J}(\boldsymbol{\theta})$ in (A.6) can be compactly expressed as

$$\boldsymbol{J}(\boldsymbol{\theta}) = \boldsymbol{A}(\boldsymbol{\theta}) + \boldsymbol{B}(\boldsymbol{\theta}),$$

where

$$\boldsymbol{A}(\boldsymbol{\theta}) = \int_{\mathcal{Z}} p_{\boldsymbol{Z}}^{1+\alpha}(\boldsymbol{z};\boldsymbol{\theta}) \big((1+\alpha)\boldsymbol{u}_{\boldsymbol{\theta}}(\boldsymbol{z}) \boldsymbol{u}_{\boldsymbol{\theta}}(\boldsymbol{z})^\top - \boldsymbol{Q}_{\boldsymbol{\theta}}(\boldsymbol{z})\big) \mathrm{d}\boldsymbol{z}$$

only depends on functions of the polyserial model, and

$$\boldsymbol{B}(\boldsymbol{\theta}) = \int_{\mathcal{Z}} f_{\varepsilon,\boldsymbol{Z}}(\boldsymbol{z}) p_{\boldsymbol{Z}}^{\alpha}(\boldsymbol{z};\boldsymbol{\theta}) \big(\boldsymbol{Q}_{\boldsymbol{\theta}}(\boldsymbol{z}) - \alpha \boldsymbol{u}_{\boldsymbol{\theta}}(\boldsymbol{z}) \boldsymbol{u}_{\boldsymbol{\theta}}(\boldsymbol{z})^\top\big) \mathrm{d}\boldsymbol{z},$$

depends on the possibly contaminated sampling density $f_{\varepsilon,\boldsymbol{Z}}$.

Recall from Section 6.1 that if the polyserial model is correctly specified ($\varepsilon = 0$), we have that the estimand corresponds to the true parameter, that is, $\boldsymbol{\theta}_0 = \boldsymbol{\theta}_*$, and, furthermore, $f_{0,\boldsymbol{Z}}(\boldsymbol{z}) = p_{\boldsymbol{Z}}(\boldsymbol{z};\boldsymbol{\theta}_*)$ for all $\boldsymbol{z} \in \boldsymbol{Z}$. In this zero-contamination case (and this case only), one can easily verify that we have for the MLE (tuning constant choice $\alpha = 0$) that

$$\int_{\mathcal{Z}} f_{\varepsilon,\boldsymbol{Z}}(\boldsymbol{z}) p_{\boldsymbol{Z}}^{2\alpha}(\boldsymbol{z};\boldsymbol{\theta}_*) \boldsymbol{u}_{\boldsymbol{\theta}_*}(\boldsymbol{z}) \boldsymbol{u}_{\boldsymbol{\theta}_*}(\boldsymbol{z})^\top \mathrm{d}\boldsymbol{z} = \boldsymbol{I}_{\boldsymbol{\theta}_*}$$

as well as

$$\begin{aligned}\boldsymbol{A}(\boldsymbol{\theta}_*) &= \boldsymbol{I}_{\boldsymbol{\theta}_*} - \boldsymbol{I}_{\boldsymbol{\theta}_*} = \boldsymbol{0},\\ \boldsymbol{B}(\boldsymbol{\theta}_*) &= \boldsymbol{I}_{\boldsymbol{\theta}_*},\\ \boldsymbol{\xi}(\boldsymbol{\theta}_*) &= \boldsymbol{0},\end{aligned}$$

so it follows from the expressions derived in Theorem A.1 that

$$\boldsymbol{\Sigma}(\boldsymbol{\theta}_*) = \boldsymbol{I}_{\boldsymbol{\theta}_*}^{-1}.$$

The fact that under correct model specification, the asymptotic covariance matrix of $\sqrt{N}\left(\widehat{\boldsymbol{\theta}}_N^{\mathrm{MLE}} - \boldsymbol{\theta}_*\right)$ equals the inverse of the Fisher information matrix at the true parameter $\boldsymbol{\theta}_*$ is a familiar property of the MLE, which is nested by Theorem A.1. Conversely, if the polyserial model is misspecified ($\varepsilon > 0$), one can show that the MLE's asymptotic covariance matrix, being $\boldsymbol{\Sigma}(\boldsymbol{\theta}_0)$ at $\alpha = 0$, is equal to well-known sandwich-type covariance matrix expressions derived by White (1982) and Huber (1967).

For strictly positive choices of $\alpha$, we obtain a more robust estimator than the MLE. However, when $\alpha > 0$ and the model is correctly specified ($\varepsilon = 0$), one can immediately see that

$$\begin{aligned}\int_{\mathcal{Z}} f_{\varepsilon,\boldsymbol{Z}}(z) p_{\boldsymbol{Z}}^{2\alpha}(z;\boldsymbol{\theta}_*)\, \boldsymbol{u}_{\boldsymbol{\theta}_*}(z)\, \boldsymbol{u}_{\boldsymbol{\theta}_*}(z)^\top \,\mathrm{d}z &\neq \boldsymbol{I}_{\boldsymbol{\theta}_*},\\ \boldsymbol{A}(\boldsymbol{\theta}_*) &\neq \boldsymbol{0},\\ \boldsymbol{B}(\boldsymbol{\theta}_*) &\neq \boldsymbol{I}_{\boldsymbol{\theta}_*},\\ \boldsymbol{\xi}(\boldsymbol{\theta}_*) &\neq \boldsymbol{0},\end{aligned}$$

which is due to the presence of terms involving $\alpha$ that do *not* vanish when $\alpha > 0$. Thus, in the absence of contamination, our estimator's asymptotic covariance matrix is different than that of the MLE. It now follows from the Cramér-Rao lower bound that our estimator is *not* efficient and, consequently, has a larger estimation variance than the MLE. As discussed in Section 6.2, a loss of efficiency at the postulated model is a common property of many robust estimators, which can be seen as the price of robustness.

### *A.6. Covariance matrix estimation*

The asymptotic covariance matrix $\boldsymbol{\Sigma_0}(\boldsymbol{\theta}_0)$ is unobserved in practice because it depends on the unknown sampling density $f_{\varepsilon,\boldsymbol{Z}}$ as well as the unknown estimand $\boldsymbol{\theta}_0$. This section explains how a consistent estimator of $\boldsymbol{\Sigma}(\boldsymbol{\theta}_0)$ can be constructed.

First, in the definitions of the population objects $\big(\boldsymbol{B}(\boldsymbol{\theta}), \boldsymbol{J}(\boldsymbol{\theta}), \boldsymbol{\xi}(\boldsymbol{\theta}), \boldsymbol{K}(\boldsymbol{\theta})\big)$, replace the unknown sampling density $f_{\varepsilon,\boldsymbol{Z}}(z)$ by its empirical counterpart in (5.2), that is,

$$\widehat{f}_N(z) = \frac{1}{N}\sum_{i=1}^{N} \mathbb{1}\{\boldsymbol{Z}_i = z\}, \qquad z \in \mathcal{Z},$$

resulting in the sample objects

$$\widehat{\boldsymbol{B}}_N(\boldsymbol{\theta}) = \frac{1}{N}\sum_{i=1}^{N} p_{XY}^{\alpha}(X_i, Y_i; \boldsymbol{\theta})\left(\boldsymbol{Q}_{\boldsymbol{\theta}}(X_i, Y_i) - \alpha \boldsymbol{u}_{\boldsymbol{\theta}}(X_i, Y_i)\, \boldsymbol{u}_{\boldsymbol{\theta}}(X_i, Y_i)^\top\right),$$
$$\widehat{\boldsymbol{J}}_N(\boldsymbol{\theta}) = \boldsymbol{A}(\boldsymbol{\theta}) + \widehat{\boldsymbol{B}}_N(\boldsymbol{\theta}),$$
$$\widehat{\boldsymbol{\xi}}_N(\boldsymbol{\theta}) = \frac{1}{N}\sum_{i=1}^{N} p_{XY}^{\alpha}(X_i, Y_i; \boldsymbol{\theta})\, \boldsymbol{u}_{\boldsymbol{\theta}}(X_i, Y_i),$$
$$\widehat{\boldsymbol{K}}_N(\boldsymbol{\theta}) = \frac{1}{N}\sum_{i=1}^{N} p_{XY}^{2\alpha}(X_i, Y_i; \boldsymbol{\theta})\, \boldsymbol{u}_{\boldsymbol{\theta}}(X_i, Y_i)\, \boldsymbol{u}_{\boldsymbol{\theta}}(X_i, Y_i)^\top - \widehat{\boldsymbol{\xi}}_N(\boldsymbol{\theta})\, \widehat{\boldsymbol{\xi}}_N(\boldsymbol{\theta})^\top.$$

Observe that the matrix $\boldsymbol{A}(\boldsymbol{\theta})$ in $\widehat{\boldsymbol{J}}_N(\boldsymbol{\theta})$ does not require a sample counterpart because it does not depend on the unknown $f_{\varepsilon,\boldsymbol{Z}}$, so it can be computed for a given parameter $\boldsymbol{\theta}$.

Basu et al. (1998) show that for a given $\boldsymbol{\theta} \in \boldsymbol{\Theta}$, the sample matrices $\left(\widehat{\boldsymbol{B}}_N(\boldsymbol{\theta}), \widehat{\boldsymbol{J}}_N(\boldsymbol{\theta}), \widehat{\boldsymbol{\xi}}_N(\boldsymbol{\theta}), \widehat{\boldsymbol{K}}_N(\boldsymbol{\theta})\right)$ are pointwise consistent estimators of their corresponding population counterparts $\left(\boldsymbol{B}(\boldsymbol{\theta}), \boldsymbol{J}(\boldsymbol{\theta}), \boldsymbol{\xi}(\boldsymbol{\theta}), \boldsymbol{K}(\boldsymbol{\theta})\right)$, as $N \to \infty$. Thus, it follows from the continuous mapping theorem that

$$\widehat{\boldsymbol{\Sigma}}_N(\boldsymbol{\theta}) = \widehat{\boldsymbol{J}}_N(\boldsymbol{\theta})^{-1}\, \widehat{\boldsymbol{K}}_N(\boldsymbol{\theta})\, \widehat{\boldsymbol{J}}_N(\boldsymbol{\theta})^{-1}$$

is pointwise consistent for $\boldsymbol{\Sigma}(\boldsymbol{\theta}) = \boldsymbol{J}(\boldsymbol{\theta})^{-1}\boldsymbol{K}(\boldsymbol{\theta})\boldsymbol{J}(\boldsymbol{\theta})^{-1}$. In particular, by $\widehat{\boldsymbol{\theta}}_N \xrightarrow{\mathbb{P}} \boldsymbol{\theta}_0$ in conjunction with the continuous mapping theorem, the matrix $\widehat{\boldsymbol{\Sigma}}_N\left(\widehat{\boldsymbol{\theta}}_N\right)$ is a consistent estimator of the asymptotic covariance matrix $\boldsymbol{\Sigma}(\boldsymbol{\theta}_0)$ in Theorem A.1.

Regarding computation, for a given $\boldsymbol{\theta} \in \boldsymbol{\Theta}$, computation of the objects $\left(\widehat{\boldsymbol{B}}_N(\boldsymbol{\theta}), \widehat{\boldsymbol{\xi}}_N(\boldsymbol{\theta}), \widehat{\boldsymbol{K}}_N(\boldsymbol{\theta})\right)$ is cheap because they only require computation of finite summations of terms with closed-form expressions. The only computational bottleneck is the matrix $\boldsymbol{A}(\boldsymbol{\theta})$, which is a component of $\widehat{\boldsymbol{J}}_N(\boldsymbol{\theta})$ and is defined as

$$\begin{aligned}\boldsymbol{A}(\boldsymbol{\theta}) &= \int_{\mathcal{Z}} p_{\boldsymbol{Z}}^{1+\alpha}(z; \boldsymbol{\theta})\left((1+\alpha)\boldsymbol{u}_{\boldsymbol{\theta}}(z)\, \boldsymbol{u}_{\boldsymbol{\theta}}(z)^\top - \boldsymbol{Q}_{\boldsymbol{\theta}}(z)\right) \mathrm{d}z \\ &= \int_{\mathbb{R}} p_X^{1+\alpha}(x; \boldsymbol{\theta}) \sum_{y \in \mathcal{Y}} p_{Y|X}^{1+\alpha}(y \mid x; \boldsymbol{\theta})\left((1+\alpha)\boldsymbol{u}_{\boldsymbol{\theta}}(x, y)\, \boldsymbol{u}_{\boldsymbol{\theta}}(x, y)^\top - \boldsymbol{Q}_{\boldsymbol{\theta}}(x, y)\right) \mathrm{d}x,\end{aligned}$$

where the second equality follows by (A.4). As such, due to the presence of the one-dimensional integral over the real numbers, the matrix $\boldsymbol{A}(\boldsymbol{\theta})$ does not posses a closed-form expression. Hence, in practice, the integral needs to be numerically approximated. Since $\boldsymbol{A}(\boldsymbol{\theta})$ is a $d \times d$ matrix, $d^2$ one-dimensional integrals need to be computed in elementwise manner. However, since $\boldsymbol{A}(\boldsymbol{\theta})$ is symmetric—owing to the symmetry of the Hessian $\boldsymbol{Q}_{\boldsymbol{\theta}}(x, y)$ and the outer matrix $\boldsymbol{u}_{\boldsymbol{\theta}}(x, y)\, \boldsymbol{u}_{\boldsymbol{\theta}}(x, y)^\top$—it suffices to only compute the $d(d+1)/2$ unique one-dimensional integrals.

As for directly estimating the Fisher information matrix, a commonly used ML-based estimator is the sample average

$$\frac{1}{N}\sum_{i=1}^{N} \boldsymbol{u}_{\widehat{\boldsymbol{\theta}}_N^{\text{MLE}}}(\boldsymbol{Z}_i)\, \boldsymbol{u}_{\widehat{\boldsymbol{\theta}}_N^{\text{MLE}}}(\boldsymbol{Z}_i)^\top,$$

which is consistent for the true Fisher information matrix $\boldsymbol{I}_{\boldsymbol{\theta}_*}$ in (A.5) if the polyserial model is correctly specified for all observations ($\varepsilon = 0$). This estimator is, for instance, the default way ML standard errors

for the polyserial model are computed in the package `lavaan` (Rosseel, 2012). Likewise, we use this estimator for computing covariance matrix estimates for the MLE.

### *A.7. Gradient and Hessian expressions*

This section derives closed-form expressions of the gradient and Hessian of the model density $p_{XY}(x, y; \boldsymbol{\theta})$ for $(x, y) \in \mathbb{R} \times \mathcal{Y}$ and

$$\boldsymbol{\theta} = \left(\rho, \mu, \sigma^2, \boldsymbol{\tau}^\top\right)^\top \in \boldsymbol{\Theta},$$

where $\boldsymbol{\tau} = (\tau_1, \ldots, \tau_{-1})^\top$, and we have the restrictions that $\rho \in (-1, 1)$, $\sigma > 0$, and $-\infty < \tau_1 < \cdots < \tau_{r-1} < \infty$. We use the density expressions used in Olsson et al. (1982) that are provided in Appendix A.2.

For further reference, the density of the univariate standard normal distribution is defined as

$$\phi(v) = \frac{1}{\sqrt{2\pi}} \exp\left(-v^2/2\right)$$

with first derivative

$$\phi'(v) = -v\phi(v)$$

and associated cumulative distribution function

$$\Phi(v) = \int_{-\infty}^{v} \phi(s)\, \mathrm{d}s,$$

for $v \in \mathbb{R}$.

#### A.7.1. First order expressions

By the chain rule and definition of $p_{XY}(x, y; \boldsymbol{\theta})$ in (A.4), it holds true that

$$\frac{\partial}{\partial \boldsymbol{\theta}} p_{XY}(x, y; \boldsymbol{\theta}) = p_X(x; \boldsymbol{\theta}) \frac{\partial p_{Y|X}(y \mid x; \boldsymbol{\theta})}{\partial \boldsymbol{\theta}} + p_{Y|X}(y \mid x; \boldsymbol{\theta}) \frac{\partial p_X(x; \boldsymbol{\theta})}{\partial \boldsymbol{\theta}},$$

where

$$\frac{\partial p_X(x; \boldsymbol{\theta})}{\partial \boldsymbol{\theta}} = \left(\frac{\partial p_X(x; \boldsymbol{\theta})}{\partial \rho}, \frac{\partial p_X(x; \boldsymbol{\theta})}{\partial \mu}, \frac{\partial p_X(x; \boldsymbol{\theta})}{\partial (\sigma^2)}, \frac{\partial p_X(x; \boldsymbol{\theta})}{\partial \tau_1}, \ldots, \frac{\partial p_X(x; \boldsymbol{\theta})}{\partial \tau_{r-1}}\right)^\top$$

is the gradient of the marginal density, and

$$\frac{\partial p_{Y|X}(y \mid x; \boldsymbol{\theta})}{\partial \boldsymbol{\theta}} = \left(\frac{\partial p_{Y|X}(y \mid x; \boldsymbol{\theta})}{\partial \rho}, \frac{\partial p_{Y|X}(y \mid x; \boldsymbol{\theta})}{\partial \mu}, \frac{\partial p_{Y|X}(y \mid x; \boldsymbol{\theta})}{\partial (\sigma^2)}, \frac{\partial p_{Y|X}(y \mid x; \boldsymbol{\theta})}{\partial \tau_1}, \ldots, \frac{\partial p_{Y|X}(y \mid x; \boldsymbol{\theta})}{\partial \tau_{r-1}}\right)^\top$$

is the gradient of the conditional density. In the following, we provide closed-form expressions for each component in these two gradients.

Starting with $\frac{\partial p_X(x;\boldsymbol{\theta})}{\partial \boldsymbol{\theta}}$, Olsson et al. (1982) show that

$$\frac{\partial p_X(x;\boldsymbol{\theta})}{\partial \mu} = p_X(x;\boldsymbol{\theta}) \frac{x-\mu}{\sigma^2}$$

and

$$\frac{\partial p_X(x;\boldsymbol{\theta})}{\partial (\sigma^2)} = \frac{p_X(x;\boldsymbol{\theta})}{2\sigma^2}\left(\left(\frac{x-\mu}{\sigma}\right)^2 - 1\right).$$

Since the marginal density only depends on $\mu$ and $\sigma^2$, all of its remaining derivatives are zero-valued, that is,

$$\frac{\partial p_X(x;\boldsymbol{\theta})}{\partial \rho} = \frac{\partial p_X(x;\boldsymbol{\theta})}{\partial \tau_1} = \cdots = \frac{\partial p_X(x;\boldsymbol{\theta})}{\partial \tau_{r-1}} = 0.$$

For the conditional density's gradient $\frac{\partial p_{Y|X}(y \mid x;\boldsymbol{\theta})}{\partial \boldsymbol{\theta}}$, Olsson et al. (1982) show that

$$\frac{\partial p_{Y|X}(y \mid x;\boldsymbol{\theta})}{\partial \rho} = \\ (1-\rho^2)^{-3/2}\left(\phi\left(\tau_y^*(x;\boldsymbol{\theta})\right)\left(\rho\tau_y - \frac{x-\mu}{\sigma}\right) - \phi\left(\tau_{y-1}^*(x;\boldsymbol{\theta})\right)\left(\rho\tau_{y-1} - \frac{x-\mu}{\sigma}\right)\right)$$

and

$$\frac{\partial p_{Y|X}(y \mid x;\boldsymbol{\theta})}{\partial \mu} = \frac{\rho}{\sigma\sqrt{1-\rho^2}}\left(\phi\left(\tau_y^*(x;\boldsymbol{\theta})\right) - \phi\left(\tau_{y-1}^*(x;\boldsymbol{\theta})\right)\right)$$

and

$$\frac{\partial p_{Y|X}(y \mid x;\boldsymbol{\theta})}{\partial (\sigma^2)} = \frac{\rho(x-\mu)}{2\sigma^3\sqrt{1-\rho^2}}\left(\phi\left(\tau_y^*(x;\boldsymbol{\theta})\right) - \phi\left(\tau_{y-1}^*(x;\boldsymbol{\theta})\right)\right)$$

and, for $k = 1,\ldots,r-1$,

$$\frac{\partial p_{Y|X}(y \mid x;\boldsymbol{\theta})}{\partial \tau_k} = \begin{cases} \phi\left(\tau_y^*(x;\boldsymbol{\theta})\right)\Big/\sqrt{1-\rho^2} & \text{if } k = y, \\ -\phi\left(\tau_{y-1}^*(x;\boldsymbol{\theta})\right)\Big/\sqrt{1-\rho^2} & \text{if } k = y-1, \\ 0 & \text{otherwise.} \end{cases}$$

### A.7.2. Ancillary derivatives

Before we turn to second order derivatives, it is useful to derive the gradient of $\tau_y^*(x;\boldsymbol{\theta})$ with respect to $\boldsymbol{\theta}$. It can be shown that

$$\frac{\partial \tau_y^*(x;\boldsymbol{\theta})}{\partial \rho} = \frac{1}{\sqrt{1-\rho^2}}\left(\frac{\tau_y^*(x;\boldsymbol{\theta})}{\sqrt{1-\rho^2}} - \frac{x-\mu}{\sigma}\right)$$

and

$$\frac{\partial \tau_y^*(x;\boldsymbol{\theta})}{\partial \mu} = \frac{\rho}{\sigma\sqrt{1-\rho^2}}$$

and

$$\frac{\partial \tau_y^*(x;\boldsymbol{\theta})}{\partial(\sigma^2)} = \frac{\rho(x-\mu)}{2\sigma^3\sqrt{1-\rho^2}}$$

and, for $k = 1, \ldots, r-1$,

$$\frac{\partial \tau_y^*(x;\boldsymbol{\theta})}{\partial \tau_k} = \begin{cases} 1\Big/\sqrt{1-\rho^2} & \text{if } k = y, \\ 0 & \text{otherwise.} \end{cases}$$

**A.7.3. Second order expressions**

The (symmetric) Hessian matrix of the marginal density is given by

$$\frac{\partial^2 p_X(x;\boldsymbol{\theta})}{\partial\boldsymbol{\theta}\partial\boldsymbol{\theta}^\top} = \begin{pmatrix} \frac{\partial^2 p_X(x;\boldsymbol{\theta})}{\partial\rho\partial\rho} & \frac{\partial^2 p_X(x;\boldsymbol{\theta})}{\partial\rho\partial\mu} & \frac{\partial^2 p_X(x;\boldsymbol{\theta})}{\partial\rho\partial(\sigma^2)} & \frac{\partial^2 p_X(x;\boldsymbol{\theta})}{\partial\rho\partial\tau_1} & \cdots & \frac{\partial^2 p_X(x;\boldsymbol{\theta})}{\partial\rho\partial\tau_{r-1}} \\ \frac{\partial^2 p_X(x;\boldsymbol{\theta})}{\partial\mu\partial\rho} & \frac{\partial^2 p_X(x;\boldsymbol{\theta})}{\partial\mu\partial\mu} & \frac{\partial^2 p_X(x;\boldsymbol{\theta})}{\partial\mu\partial(\sigma^2)} & \frac{\partial^2 p_X(x;\boldsymbol{\theta})}{\partial\mu\partial\tau_1} & \cdots & \frac{\partial^2 p_X(x;\boldsymbol{\theta})}{\partial\mu\partial\tau_{r-1}} \\ \frac{\partial^2 p_X(x;\boldsymbol{\theta})}{\partial(\sigma^2)\partial\rho} & \frac{\partial^2 p_X(x;\boldsymbol{\theta})}{\partial(\sigma^2)\partial\mu} & \frac{\partial^2 p_X(x;\boldsymbol{\theta})}{\partial(\sigma^2)\partial(\sigma^2)} & \frac{\partial^2 p_X(x;\boldsymbol{\theta})}{\partial(\sigma^2)\partial\tau_1} & \cdots & \frac{\partial^2 p_X(x;\boldsymbol{\theta})}{\partial(\sigma^2)\partial\tau_{r-1}} \\ \frac{\partial^2 p_X(x;\boldsymbol{\theta})}{\partial\tau_1\partial\rho} & \frac{\partial^2 p_X(x;\boldsymbol{\theta})}{\partial\tau_1\partial\mu} & \frac{\partial^2 p_X(x;\boldsymbol{\theta})}{\partial\tau_1\partial(\sigma^2)} & \frac{\partial^2 p_X(x;\boldsymbol{\theta})}{\partial\tau_1\partial\tau_1} & \cdots & \frac{\partial^2 p_X(x;\boldsymbol{\theta})}{\partial\tau_1\partial\tau_{r-1}} \\ \vdots & \vdots & \vdots & \vdots & \ddots & \vdots \\ \frac{\partial^2 p_X(x;\boldsymbol{\theta})}{\partial\tau_{r-1}\partial\rho} & \frac{\partial^2 p_X(x;\boldsymbol{\theta})}{\partial\tau_{r-1}\partial\mu} & \frac{\partial^2 p_X(x;\boldsymbol{\theta})}{\partial\tau_{r-1}\partial(\sigma^2)} & \frac{\partial^2 p_X(x;\boldsymbol{\theta})}{\partial\tau_{r-1}\partial\tau_1} & \cdots & \frac{\partial^2 p_X(x;\boldsymbol{\theta})}{\partial\tau_{r-1}\partial\tau_{r-1}} \end{pmatrix}, \tag{A.7}$$

and the Hessian of the conditional density $\frac{\partial^2 p_{Y|X}(y \mid x;\boldsymbol{\theta})}{\partial\boldsymbol{\theta}\partial\boldsymbol{\theta}^\top}$ is constructed analogously by replacing $p_X(x;\boldsymbol{\theta})$ by $p_{Y|X}(y \mid x;\boldsymbol{\theta})$ in (A.7). We proceed by deriving the second order derivatives of both the marginal and the conditional density.

For the second order derivatives of the marginal density $p_X(x;\boldsymbol{\theta})$, tedious but straightforward applications of the chain and product rule yield

$$\frac{\partial^2 p_X(x;\boldsymbol{\theta})}{\partial\mu\partial\mu} = \frac{1}{\sigma^2}\left((x-\mu)\frac{\partial p_X(x;\boldsymbol{\theta})}{\partial\mu} - p_X(x;\boldsymbol{\theta})\right)$$

and

$$\frac{\partial^2 p_X(x;\boldsymbol{\theta})}{\partial\mu\partial\sigma^2} = \frac{x-\mu}{\sigma^2}\left(\frac{\partial p_X(x;\boldsymbol{\theta})}{\partial(\sigma^2)} - \frac{p_X(x;\boldsymbol{\theta})}{\sigma^2}\right)$$

and

$$\frac{\partial^2 p_X(x;\boldsymbol{\theta})}{\partial(\sigma^2)\partial(\sigma^2)} = \frac{1}{2\sigma^2}\left(\frac{\partial p_X(x;\boldsymbol{\theta})}{\partial(\sigma^2)}\left(\left(\frac{x-\mu}{\sigma}\right)^2 - 1\right) + \frac{p_X(x;\boldsymbol{\theta})}{\sigma^2}\left(1 - 2\left(\frac{x-\mu}{\sigma}\right)^2\right)\right).$$

Because the marginal density only depends on $\mu$ and $\sigma^2$, its remaining second order derivatives are all zero. It follows that among all unique elements in the Hessian in (A.7), only $\frac{\partial^2 p_X(x;\boldsymbol{\theta})}{\partial\mu\partial\mu}$, $\frac{\partial^2 p_X(x;\boldsymbol{\theta})}{\partial\mu\partial(\sigma^2)}$, and $\frac{\partial^2 p_X(x;\boldsymbol{\theta})}{\partial(\sigma^2)\partial(\sigma^2)}$ are nonzero.

For the second order derivatives of the conditional density $p_{Y|X}(y \mid x; \boldsymbol{\theta})$, tedious but straightforward applications of the chain and product rule yield

$$\frac{\partial^2 p_{Y|X}(y \mid x; \boldsymbol{\theta})}{\partial\rho\partial\rho} =$$
$$\frac{3\rho}{(1-\rho^2)^{5/2}}\left(\phi\left(\tau_y^*(x;\boldsymbol{\theta})\right)\left(\rho\tau_y - \frac{x-\mu}{\sigma}\right) - \phi\left(\tau_{y-1}^*(x;\boldsymbol{\theta})\right)\left(\rho\tau_{y-1} - \frac{x-\mu}{\sigma}\right)\right) +$$
$$\frac{1}{(1-\rho^2)^{3/2}}\Bigg[\phi'\left(\tau_y^*(x;\boldsymbol{\theta})\right)\left(\rho\tau_y - \frac{x-\mu}{\sigma}\right)\frac{\partial\tau_y^*(x;\boldsymbol{\theta})}{\partial\rho} + \tau_y\phi\left(\tau_y^*(x;\boldsymbol{\theta})\right) -$$
$$\phi'\left(\tau_{y-1}^*(x;\boldsymbol{\theta})\right)\left(\rho\tau_{y-1} - \frac{x-\mu}{\sigma}\right)\frac{\partial\tau_{y-1}^*(x;\boldsymbol{\theta})}{\partial\rho} - \tau_{y-1}\phi\left(\tau_{y-1}^*(x;\boldsymbol{\theta})\right)\Bigg]$$

and

$$\frac{\partial^2 p_{Y|X}(y \mid x; \boldsymbol{\theta})}{\partial\mu\partial\mu} = \frac{\rho}{\sigma\sqrt{1-\rho^2}}\left(\phi'\left(\tau_y^*(x;\boldsymbol{\theta})\right)\frac{\partial\tau_y^*(x;\boldsymbol{\theta})}{\partial\mu} - \phi'\left(\tau_{y-1}^*(x;\boldsymbol{\theta})\right)\frac{\partial\tau_{y-1}^*(x;\boldsymbol{\theta})}{\partial\mu}\right)$$

and

$$\frac{\partial^2 p_{Y|X}(y \mid x; \boldsymbol{\theta})}{\partial(\sigma^2)\partial(\sigma^2)} = \frac{\rho(x-\mu)}{2\sigma^3\sqrt{1-\rho^2}}\Bigg[-\frac{3}{2\sigma^2}\left(\phi\left(\tau_y^*(x;\boldsymbol{\theta})\right) - \phi\left(\tau_{y-1}^*(x;\boldsymbol{\theta})\right)\right) +$$
$$\phi'\left(\tau_y^*(x;\boldsymbol{\theta})\right)\frac{\partial\tau_y^*(x;\boldsymbol{\theta})}{\partial(\sigma^2)} - \phi'\left(\tau_{y-1}^*(x;\boldsymbol{\theta})\right)\frac{\partial\tau_{y-1}^*(x;\boldsymbol{\theta})}{\partial(\sigma^2)}\Bigg]$$

and, for $k = 1, \dots, r-1$,

$$\frac{\partial^2 p_{Y|X}(y \mid x; \boldsymbol{\theta})}{\partial\tau_k\partial\tau_k} = \begin{cases} \frac{1}{\sqrt{1-\rho^2}}\phi'\left(\tau_y^*(x;\boldsymbol{\theta})\right)\frac{\partial\tau_y^*(x;\boldsymbol{\theta})}{\partial\tau_y} & \text{if } k = y, \\ -\frac{1}{\sqrt{1-\rho^2}}\phi'\left(\tau_{y-1}^*(x;\boldsymbol{\theta})\right)\frac{\partial\tau_{y-1}^*(x;\boldsymbol{\theta})}{\partial\tau_{y-1}} & \text{if } k = y-1, \\ 0 & \text{otherwise.} \end{cases}$$

For the cross-derivatives, it can be shown that

$$\frac{\partial^2 p_{Y|X}(y \mid x; \boldsymbol{\theta})}{\partial\rho\partial\mu} = \frac{1}{\sigma\sqrt{1-\rho^2}}\Bigg[\left(1 + \frac{\rho^2}{1-\rho^2}\right)\left(\phi\left(\tau_y^*(x;\boldsymbol{\theta})\right) - \phi\left(\tau_{y-1}^*(x;\boldsymbol{\theta})\right)\right) +$$
$$\rho\left(\phi'\left(\tau_y^*(x;\boldsymbol{\theta})\right)\frac{\partial\tau_y^*(x;\boldsymbol{\theta})}{\partial\rho} - \phi'\left(\tau_{y-1}^*(x;\boldsymbol{\theta})\right)\frac{\partial\tau_{y-1}^*(x;\boldsymbol{\theta})}{\partial\rho}\right)\Bigg]$$

and

$$\frac{\partial^2 p_{Y|X}(y \mid x;\boldsymbol{\theta})}{\partial\rho\partial(\sigma^2)} = \frac{x-\mu}{2\sigma^3\sqrt{1-\rho^2}}\left[\left(1+\frac{\rho^2}{1-\rho^2}\right)\left(\phi\left(\tau_y^*(x;\boldsymbol{\theta})\right)-\phi\left(\tau_{y-1}^*(x;\boldsymbol{\theta})\right)\right)+\right.$$
$$\left.\rho\left(\phi'\left(\tau_y^*(x;\boldsymbol{\theta})\right)\frac{\partial\tau_y^*(x;\boldsymbol{\theta})}{\partial\rho}-\phi'\left(\tau_{y-1}^*(x;\boldsymbol{\theta})\right)\frac{\partial\tau_{y-1}^*(x;\boldsymbol{\theta})}{\partial\rho}\right)\right]$$

and

$$\frac{\partial^2 p_{Y|X}(y \mid x;\boldsymbol{\theta})}{\partial(\sigma^2)\partial\mu} = \frac{\rho}{\sigma\sqrt{1-\rho^2}}\left[\phi'\left(\tau_y^*(x;\boldsymbol{\theta})\right)\frac{\partial\tau_y^*(x;\boldsymbol{\theta})}{\partial(\sigma^2)}-\phi'\left(\tau_{y-1}^*(x;\boldsymbol{\theta})\right)\frac{\partial\tau_{y-1}^*(x;\boldsymbol{\theta})}{\partial(\sigma^2)}-\right.$$
$$\left.\frac{1}{2\sigma^2}\left(\phi\left(\tau_y^*(x;\boldsymbol{\theta})\right)-\phi\left(\tau_{y-1}^*(x;\boldsymbol{\theta})\right)\right)\right]$$

and, for $k=1,\ldots,r-1$,

$$\frac{\partial^2 p_{Y|X}(y \mid x;\boldsymbol{\theta})}{\partial\mu\partial\tau_k} = \begin{cases}\frac{\rho}{\sigma(1-\rho^2)}\phi'\left(\tau_y^*(x;\boldsymbol{\theta})\right) & \text{if } k=y,\\ -\frac{\rho}{\sigma(1-\rho^2)}\phi'\left(\tau_{y-1}^*(x;\boldsymbol{\theta})\right) & \text{if } k=y-1,\\ 0 & \text{otherwise,}\end{cases}$$

and

$$\frac{\partial^2 p_{Y|X}(y \mid x;\boldsymbol{\theta})}{\partial(\sigma^2)\partial\tau_k} = \begin{cases}\frac{\rho(x-\mu)}{2\sigma^3(1-\rho^2)}\phi'\left(\tau_y^*(x;\boldsymbol{\theta})\right) & \text{if } k=y,\\ -\frac{\rho(x-\mu)}{2\sigma^3(1-\rho^2)}\phi'\left(\tau_{y-1}^*(x;\boldsymbol{\theta})\right) & \text{if } k=y-1,\\ 0 & \text{otherwise,}\end{cases}$$

and

$$\frac{\partial^2 p_{Y|X}(y \mid x;\boldsymbol{\theta})}{\partial\rho\partial\tau_k} = \begin{cases}\frac{\partial}{\partial\rho}\left[\frac{\phi\left(\tau_y^*(x;\boldsymbol{\theta})\right)}{\sqrt{1-\rho^2}}\right] & \text{if } k=y,\\ -\frac{\partial}{\partial\rho}\left[\frac{\phi\left(\tau_{y-1}^*(x;\boldsymbol{\theta})\right)}{\sqrt{1-\rho^2}}\right] & \text{if } k=y-1,\\ 0 & \text{otherwise,}\end{cases}$$

where

$$\frac{\partial}{\partial\rho}\left[\frac{\phi\left(\tau_k^*(x;\boldsymbol{\theta})\right)}{\sqrt{1-\rho^2}}\right] = \frac{\rho}{(1-\rho^2)^{3/2}}\phi\left(\tau_k^*(x;\boldsymbol{\theta})\right)+\frac{1}{\sqrt{1-\rho^2}}\phi'\left(\tau_k^*(x;\boldsymbol{\theta})\right)\frac{\partial\tau_k^*(x;\boldsymbol{\theta})}{\partial\rho},$$

for $k\in\{1,\ldots,r-1\}$, and, finally,

$$\frac{\partial^2 p_{Y|X}(y \mid x;\boldsymbol{\theta})}{\partial\tau_i\partial\tau_j}=0$$

for $i\neq j$.

We verified the correctness of all analytical expressions of derivatives provided in this Appendix A.7 using numerical approximations. The corresponding code is provided in the online replication material.

## B. Algorithm for upper bound of raw weights

Recall from Section 5.3 that the nonnegative individual-specific raw weights in estimating equation (5.7) are defined as

$$\widetilde{w}_{i,\alpha}(\boldsymbol{\theta}) = p_{XY}^{\alpha}\left(X_i, Y_i; \boldsymbol{\theta}\right), \qquad i = 1, \ldots, N,$$

which are bounded from below by 0 and bounded from above by

$$M_\alpha(\boldsymbol{\theta}) = \sup\left\{p_{XY}^{\alpha}\left(x, y; \boldsymbol{\theta}\right) : x \in \mathbb{R}, y \in \mathcal{Y}\right\}. \tag{B.1}$$

The upper bound $M_\alpha(\boldsymbol{\theta})$ does not depend on observed data and is finite because it is assumed in the polyserial model that $\sigma^2 > 0$ and $\rho \in (-1, 1)$. We require this upper bound to construct the rescaled weights

$$w_{i,\alpha}(\boldsymbol{\theta}) = \widetilde{w}_{i,\alpha}(\boldsymbol{\theta}) \big/ M_\alpha(\boldsymbol{\theta})$$

in Equation (5.9) so that the (rescaled) weights take values in $[0, 1]$. In the following, we describe an algorithm to compute the upper bound $M_\alpha(\boldsymbol{\theta})$. The algorithm exploits the fact that the domain of the second argument of $p_{XY}^{\alpha}\left(\cdot, \cdot; \boldsymbol{\theta}\right)$ is finite, namely $\mathcal{Y}$.

0. Fix $\boldsymbol{\theta} = (\rho, \mu, \sigma^2, \tau_1, \ldots, \tau_{r-1})^\top \in \boldsymbol{\Theta}$ and $\alpha > 0$. Usually, these are respectively an estimate $\widehat{\boldsymbol{\theta}}_N$ and the tuning constant used to obtain that estimate in problem (5.6).
1. Fix a response option $y \in \mathcal{Y}$ and maximize the objective function

$$S_y(x) = p_{XY}^{\alpha}\left(x, y; \boldsymbol{\theta}\right) \tag{B.2}$$

   with respect to $x \in \mathbb{R}$, keeping $y$ fixed. Denote by $S_y^*$ the value of the objective at the argmax.
2. Repeat Step 1 for all $y \in \mathcal{Y}$ and return the upper bound $M_\alpha(\boldsymbol{\theta}) = \max\left\{S_y^* : y \in \mathcal{Y}\right\}$.

Note that the scalar gradient of the objective $S_y(x)$ in (B.2) can be shown to be equal to

$$\alpha p_{XY}^{\alpha-1}\left(x, y; \boldsymbol{\theta}\right) \int_{\tau_{y-1}}^{\tau_y} \left(\frac{\partial}{\partial x} p_{X\eta}\left(x, v; \boldsymbol{\theta}\right)\right) \mathrm{d}v,$$

which follows by the chain rule in conjunction with the Leibniz integral rule, and where

$$\frac{\partial}{\partial x} p_{X\eta}\left(x, v; \boldsymbol{\theta}\right) = -\frac{1}{\sigma(1-\rho^2)}\left(\frac{x-\mu}{\sigma} - \rho v\right) p_{X\eta}\left(x, v; \boldsymbol{\theta}\right), \qquad x, v \in \mathbb{R},$$

which follows by the definition of the bivariate normal density corresponding to the distribution in (3.2). This gradient can be used for maximizing the objective $S_y(x)$ by using standard methods for numerical optimization. In our implementation, we use the BFGS algorithm (e.g., Nocedal & Wright, 2006, Section 6.1) and initialize at $x = \mu$.

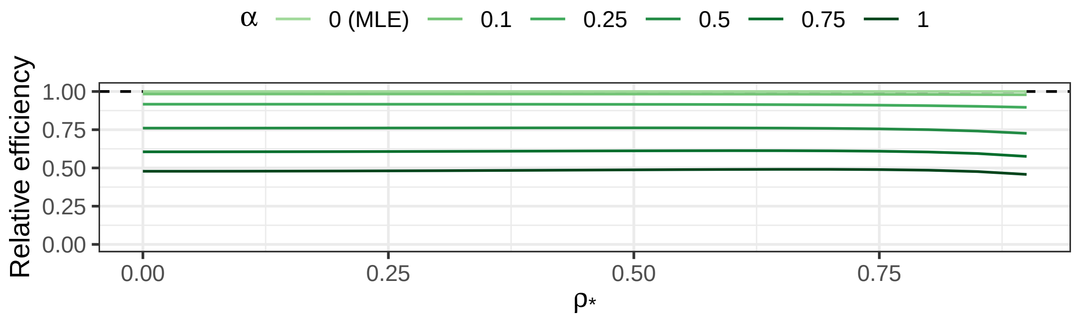


Figure C.1: Relative efficiency of the robust estimator (with respect to the MLE) for various choices of the tuning constant $\alpha$ (lines), expressed as a function of the true polyserial correlation coefficient $\rho_*$ ($x$-axis) where the remaining true parameters are fixed at $\left(\mu_*, \sigma_*^2, \tau_{*,1}, \tau_{*,2}, \tau_{*,3}, \tau_{*,4}\right)^\top = (0, 1, -1.5, -0.5, 0.5, 1.5)^\top$.

## C. Additional results from the main text

### *C.1. Additional results for efficiency*

Table 1 in Section 6.3 lists the relative efficiency (with respect to the MLE) of the robust estimator at the true parameter vector $\boldsymbol{\theta}_* = \left(\rho_*, \mu_*, \sigma_*^2, \tau_{*,1}, \tau_{*,2}, \tau_{*,3}, \tau_{*,4}\right)^\top = (0.5, 0, 1, -1.5, -0.5, 0.5, 1.5)^\top$, for various choices of tuning constant $\alpha$. Figure C.1 visualizes the relative efficiencies as a function of the true polyserial correlation coefficient $\rho_*$ with the remaining parameters in $\boldsymbol{\theta}_*$ fixed. The relative efficiencies of the robust estimators stay nearly constant, except for a small dip towards large correlations of about $\rho_* = 0.9$. Our package `robcat` provides functionality to compute statistical efficiency at custom values of $\boldsymbol{\theta}_*$.

### *C.2. Definition of performance measures*

A performance measure used in Section 7 is the approximate bias of the standard error estimation, which is defined as the difference between the average estimated standard error (averages over the simulation repetitions) and the standard deviation of the simulated sampling distribution of the correlation estimates. We rigorously define this performance measure in the following.

Denote by $\widehat{\rho}_N^{(t)}$ an estimate of the polyserial correlation coefficient in a $t$-th simulation repetition, $t = 1, \ldots, T$, where $T = 5,000$ for all simulation studies in this paper. The sample standard deviation of the $T$ individual correlation estimates is given by

$$\mathrm{SE}^{\mathrm{approx}}\left(\widehat{\rho}_N\right) = \sqrt{\frac{1}{T-1}\sum_{t=1}^{T}\left(\widehat{\rho}_N^{(t)} - \widehat{\rho}_N^{\mathrm{ave}}\right)^2},$$

where

$$\widehat{\rho}_N^{\mathrm{ave}} = \frac{1}{T}\sum_{t=1}^{T}\widehat{\rho}_N^{(t)},$$

is the sample mean of the estimates. The estimates' sample standard deviation $\mathrm{SE}^{\mathrm{approx}}\left(\widehat{\rho}_N\right)$ is an approximation to the unknown finite-sample standard error of the correlation estimator. While an asymptotic expression of the standard error exists—being the square root of the top left element of $\boldsymbol{\Sigma}\left(\boldsymbol{\theta}_0\right)$ in Theorem A.1—the true standard error $\mathrm{SE}\left(\widehat{\rho}_N\right)$ in finite samples is unknown. We therefore use the sample standard deviation $\mathrm{SE}^{\mathrm{approx}}\left(\widehat{\rho}_N\right)$ as an approximation.

The performance measure of the approximate bias of standard error estimation is now defined as

$$\widehat{\mathrm{SE}}^{\mathrm{ave}}\left(\widehat{\rho}_N\right) - \mathrm{SE}^{\mathrm{approx}}\left(\widehat{\rho}_N\right),$$

where

$$\widehat{\mathrm{SE}}^{\mathrm{ave}}\left(\widehat{\rho}_N\right) = \frac{1}{T}\sum_{t=1}^{T}\widehat{\mathrm{SE}}\left(\widehat{\rho}_N^{(t)}\right),$$

is the sample mean of the individual correlation standard error estimates. If the asymptotic theory in Theorem A.1 is correct, then, for a sufficiently large sample size, $\widehat{\mathrm{SE}}^{\mathrm{ave}}\left(\widehat{\rho}_N\right)$ should be close to the finite sample approximation for the true standard error, $\mathrm{SE}^{\mathrm{approx}}\left(\widehat{\rho}_N\right)$.

### *C.3. Additional simulation results*

Figure 2 in Section 7 visualizes the bias of the estimates for the polyserial correlation coefficient in the employed simulation. Since this coefficient is only one of the $d$ parameters of the polyserial model (alongside the population mean and variance of $X$ as well as the thresholds underlying $Y$), it is of interest to also assess the estimation accuracy for the remaining parameters in the parameter vector $\boldsymbol{\theta} = (\rho, \mu, \sigma^2, \boldsymbol{\tau}^\top)^\top$. To compare an estimate $\widehat{\boldsymbol{\theta}}_N$ to the true value $\boldsymbol{\theta}_*$, we use a geometric approach, namely the angle between $\widehat{\boldsymbol{\theta}}_N$ and $\boldsymbol{\theta}_*$, which is defined as

$$\mathrm{angle}\left(\widehat{\boldsymbol{\theta}}_N, \boldsymbol{\theta}_*\right) = \frac{180}{\pi}\cdot\cos^{-1}\left(\frac{\left|\boldsymbol{\theta}_*^\top\widehat{\boldsymbol{\theta}}_N\right|}{\|\boldsymbol{\theta}_*\|\|\widehat{\boldsymbol{\theta}}_N\|}\right),$$

where $\|\cdot\|$ denotes the Euclidean norm. Multiplying by the constant $180/\pi$ transforms the measurement unit from radians to degrees. Hence, the closer the angle to zero degrees, the better the estimation accuracy. Conversely, an angle of ninety degrees indicates a poor performance because the two vectors are perpendicular to one another. As performance measure over $T$ repetitions, we use the root mean squared error (RMSE) of the squared angles, that is,

$$\mathrm{RMSE} = \sqrt{\frac{1}{T}\sum_{t=1}^{T}\mathrm{angle}^2\left(\widehat{\boldsymbol{\theta}}_N^{(t)}, \boldsymbol{\theta}_*\right)},$$

where $\widehat{\boldsymbol{\theta}}_N^{(t)}$ is the estimate of the $t$-th repetition. This performance measure has been used before in the robust statistics literature, like in, e.g., Alfons et al. (2017).

Figure C.2 visualizes the RMSE (in degrees) of the different estimators across the considered contamination fractions. The results are very similar in quality as those for estimating the polyserial correlation coefficient in Figure 2: As soon as contamination is present, the MLE starts to exhibit a notable bias, and stabilizes at about $\varepsilon = 0.1$ with an angle of about $60^\circ$. Conversely, the robust estimators ($\alpha > 0$) are more accurate in the presence of contamination until they break gradually start to down as

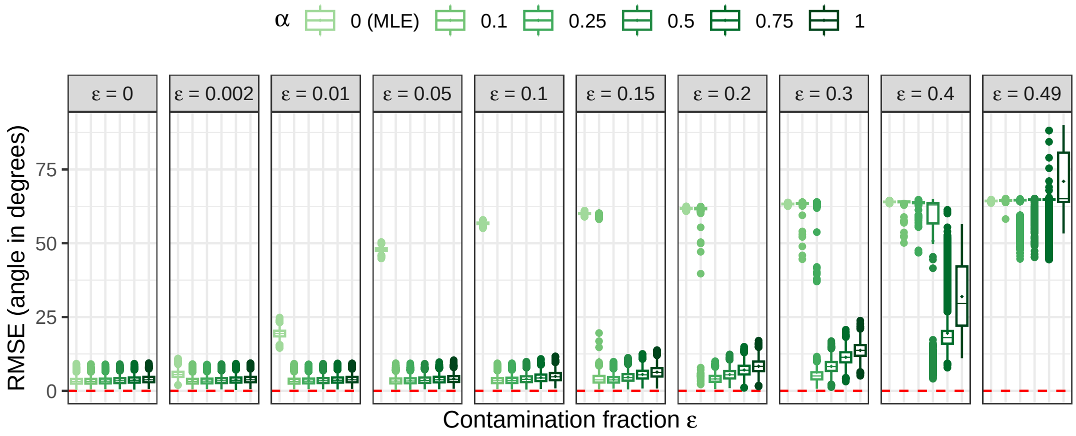


Figure C.2: Boxplots of the RMSE (in degrees) of the considered estimators, for various contamination fractions in the misspecified polyserial models across 5,000 repetitions. Diamonds represent the respective average RMSE.

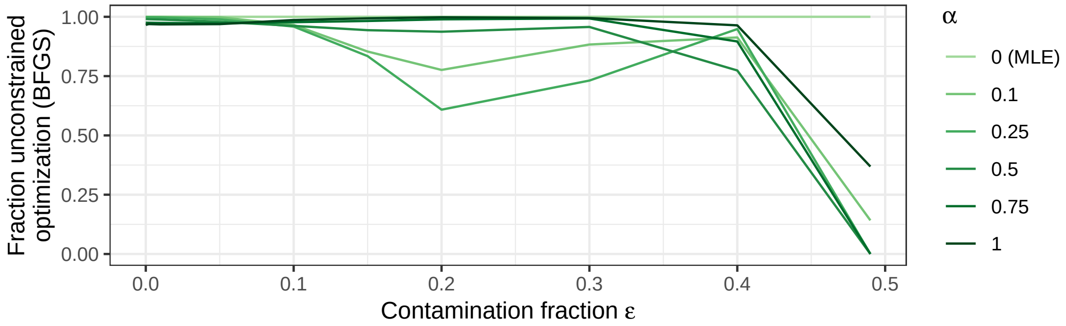


Figure C.3: Fraction of the 5,000 simulation repetitions in which unconstrained optimization via BFGS did not converge, so constrained Nelder-Mead optimization was subsequently used instead.

well as the contamination fraction is increased. Beyond $\varepsilon = 0.4$, all estimators have broken down and perform similarly poorly.

Recall from Section 6.4 that by default, our implementation first tries unconstrained optimization via the BFGS algorithm, and, in case of nonconvergence or an error, subsequently tries constrained optimization via the Nelder-Mead algorithm. Figure C.3 visualizes the frequency with which unconstrained optimization converged. In case of low contamination fractions, unconstrained optimization nearly always succeeded. Conversely, as the contamination fraction increases to higher values, unconstrained optimization fails more often, so a constrained algorithm must be used instead. Similar behavior was observed in all other simulations in this paper.

Furthermore, Figure C.4 illustrates the fraction of the 5,000 repetitions in which the correlation estimate $\widehat{\rho}_N$ of given estimator $\widehat{\boldsymbol{\theta}}_N$ did not numerically converge to a solution, neither with the unconstrained BFGS nor the constrained Nelder-Mead algorithm (see Section 6.4). In similar fashion, it visualizes the

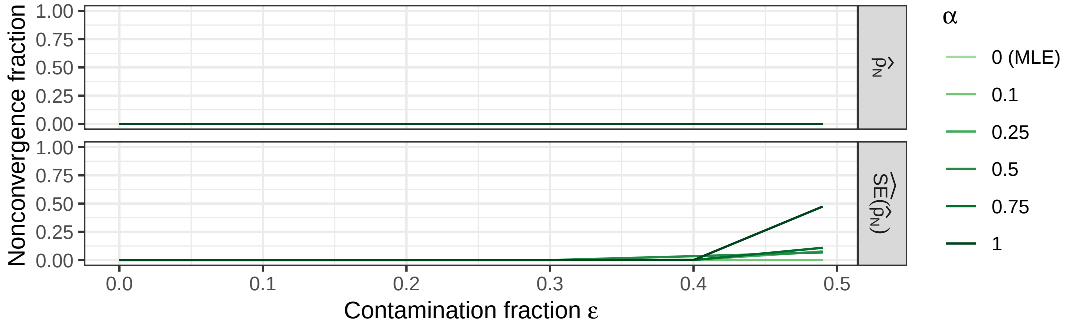


Figure C.4: Fraction of the 5,000 simulation repetitions in which a point estimator did not numerically converge—neither with the unconstrained BFGS nor the constrained Nelder-Mead algorithm—(top), and its associated standard error could not be computed due to singularity of $\widehat{\boldsymbol{J}}_N\left(\widehat{\boldsymbol{\theta}}_N\right)$ in the covariance matrix estimator (bottom). The point estimators always converged, resulting in overplotted lines.

fraction in which its associated estimated standard error $\widehat{\mathrm{SE}}\left(\widehat{\rho}_N\right)$ could not be computed due to singularity of matrix $\widehat{\boldsymbol{J}}_N\left(\widehat{\boldsymbol{\theta}}_N\right)$ in the covariance matrix estimator in Section 6.2. The point estimates $\widehat{\rho}_N$ always numerically converged to a solution across all configurations and repetitions. Up to (and including) contamination fraction $\varepsilon = 0.3$, the standard errors could virtually always be computed. Only in extremely high-contamination settings with $\varepsilon > 0.3$, as the estimators gradually break down (Figure C.2), standard errors could occasionally also not be computed. In particular, at the highest considered contamination fraction $\varepsilon = 0.49$, the estimator with tuning constant $\alpha = 1$ fails to compute standard errors in about half of the repetitions. However, this estimator itself seems to be unstable in this setting to begin with (see Figure 2), which may cause frequent singularity of matrix $\widehat{\boldsymbol{J}}_N\left(\widehat{\boldsymbol{\theta}}_N\right)$.

### *C.4. Discussion on robustifying the two-step approach*

As described in Section 3.3, polyserial correlation is often estimated through the two-step approach in (3.7), which is computationally faster but less efficient than joint ML estimation. It turns out that the two-step approach is also a maximum likelihood (ML) estimator in which model parameters are estimated sequentially rather than jointly. In particular, it can be shown (Muthén, 1984) that

- the first-step estimators $\left(\widehat{\mu}_{\mathrm{TS}}, \widehat{\sigma}^2_{\mathrm{TS}}\right)$ of $(\mu, \sigma^2)$ are ML estimates of a marginal location-scale normality model for $X$;
- the first-step threshold estimators $\widehat{\tau}_{k,\mathrm{TS}}$ are ML estimates of thresholds $\tau_k, k = 1, \dots, r-1$, in marginal latent normality model for $Y$,
- the second-step correlation estimator of $\rho$ is a conditional ML estimator in a bivariate normality model, conditional on the first-step estimates.

It is therefore natural to ask whether the two-step approach can also be robustified by using DPD estimators. Indeed, it is possible to apply separate DPD estimators to separately estimate each of the three models above in a robust manner, owing to the generality of the results in Basu et al. (1998). However, there are substantial theoretical and computational downsides of such a robustified two-stage approach, as we shall explain in the following.

First, using DPD for jointly estimating all model parameters already incurs an efficiency loss, as discussed in detail in Section 6.3. Since using DPD in a two-stage approach requires fitting three separate models, the efficiency loss of such a robustified two-step approach would potentially be substantial due to using three estimation procedures with diminished efficiency. One of the attractive features of joint DPD estimation (as proposed in Section 5) is that robustness can be gained with only a relatively minor efficiency loss of less than 2% (see Section 6.3).

Second, every DPD estimator requires numerically solving an integral over the postulated model's density to the power of $(1+\alpha)$ in every iteration of the employed optimization routine (Basu et al., 1998, Eq. 2.2). This numerical integral is the main computational bottleneck that causes the robust estimator in Section 5 to be computationally slower than joint ML. Recall that in our case, the integral is one-dimensional because the $Y$-dimension is a computationally cheap sum of $r$ summands; see Eq. (5.5). In a robustified two-step approach, however, there are three separate DPD estimators, each of which requires numerically solving a one-dimensional integral in every iteration. Hence, we expect the robustified two-stage approach to take even *longer* to compute than simultaneous estimation because the former requires separately solving three univariate integrals, while the latter only needs to solve one. While the main advantage of the two-step approach as in Olsson et al. (1982) is reduced computing time, such an advantage would therefore be absent (even reversed) in a DPD-based robustification thereof.

Thus, overall, robustifying the two-step approach via DPD would result in a procedure that is slower to compute and less efficient than joint estimation. Consequently, we believe that the latter approach is strictly preferable to the former.

## D. Additional simulations

### *D.1. Contamination through gross errors*

This simulation is concerned with *gross errors*, that is, extreme outliers. We are interested in how ML and our robust estimator react to such observations.

For the true polyserial model parameters, we choose the same configurations as in Section 7, namely $\rho_* = 0.5, \mu_* = 0, \sigma_*^2 = 1, \tau_{*,1} = -1.5, \tau_{*,2} = -0.5, \tau_{*,3} = 0.5, \tau_{*,4} = 1.5$, so that the ordinal variable again has five response categories. As contaminating distribution of $(X, \eta)$, we set a bivariate normal distribution with covariance matrix equal to identity and population mean $(a, -a)^\top$, where $a = 1,000,000$, and discretize the ensuing realizations in the $Y$-dimension with thresholds $\tau_{*,j}, j = 1, \dots, 4$. Thus, contamination manifests through extreme outliers in the $X$-dimension and inflation of the first response category in the $Y$-dimension. We consider the same contamination fractions as in Section 7, namely $\varepsilon \in \{0, 0.002, 0.01, 0.05, 0.1, 0.15, 0.2, 0.3, 0.4, 0.49\}$, as well as the same sample size, $N = 500$, number of repetitions, $T = 5,000$, and performance measures.

Figure D.1 visualize the simulation results. Already one single gross error observation ($\varepsilon = 0.002$ with the considered sample size) suffices for the MLE to suffer from a sign flip: While the true value is moderately positive ($\rho_* = 0.5$), the ML correlation estimates are always negative. Furthermore, the extreme values of the outliers cause the Fisher information matrix to become (computationally) singular, so no inferential statistics could be computed for the MLE for any positive contamination fraction in any repetition. Conversely, the robust estimators stay nearly unaffected by the presence of such gross errors up until about $\varepsilon = 0.2$ with accurate point estimates and coverage at the nominal level of 95%. Beyond $\varepsilon = 0.2$, the coverage of the robust estimators slightly decreases, but the point estimates remain fairly accurate. Only at the extreme contamination level of $\varepsilon = 0.49$ considerable bias occurs also for the robust estimators.

Interestingly, the robust estimator is more accurate under gross error contamination than under the less extreme contamination in Section 7 (see Figure 2). It seems plausible that gross error contamination is easier to distinguish from regular observations from the polyserial model than less extreme forms of contamination. Consequently, it might be easier for the robust estimator to downweight the correct observations compared to a situation when contamination and regular observations are more alike.

For completeness, Figure D.2 summarizes numerical nonconvergence. The point estimators always converged. With a positive contamination fraction $\varepsilon > 0$, ML standard errors could never be computed due to the normality-based Fisher information matrix becoming singular in this setting, further reflecting the instability of ML in the presence of gross errors.

### *D.2. Contamination through correlation shift*

The simulations in Sections 7 and D.1 have been concerned with mean-shifted contamination, that is, the contamination has been generated by a contamination distribution of $(X, \eta)$ whose population mean differs from the population mean of the true normal distribution $P_{X\eta}(\cdot, \cdot; \boldsymbol{\theta}_*)$. However, contamination may manifest in an unlimited variety of ways, with some not characterized by mean shifts but *correlation shifts*. A correlation shift occurs if the contamination distribution has the same population means and variances as the true distribution, but a different correlation structure. In our context of polyserial correlation, the contamination distribution $H_{X\eta}$ is equal to the true normal distribution $P_{X\eta}(\cdot, \cdot; \boldsymbol{\theta}_*)$, except for a sign-flipped correlation parameter, $-\rho_*$.

In this simulation, we consider partial misspecification of the polyserial model through correlation-shifted contamination. As true parameter values, we set $\mu = 0, \sigma^2 = 1$, and $\boldsymbol{\tau}_*^\top = (-1.5, -0.5, 0.5, 1.5)^\top$

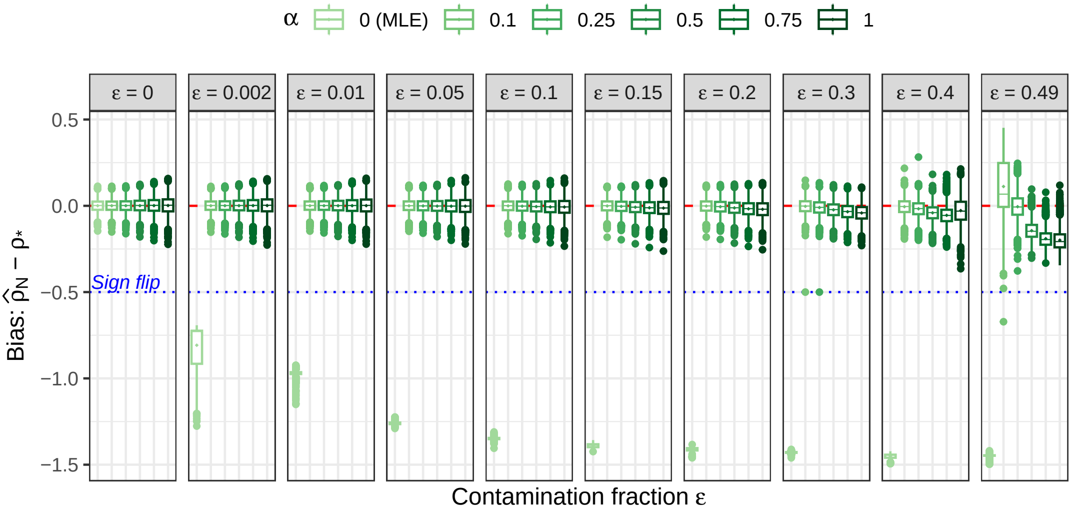


(a) Boxplot visualization of the bias. Diamonds represent the respective average bias.

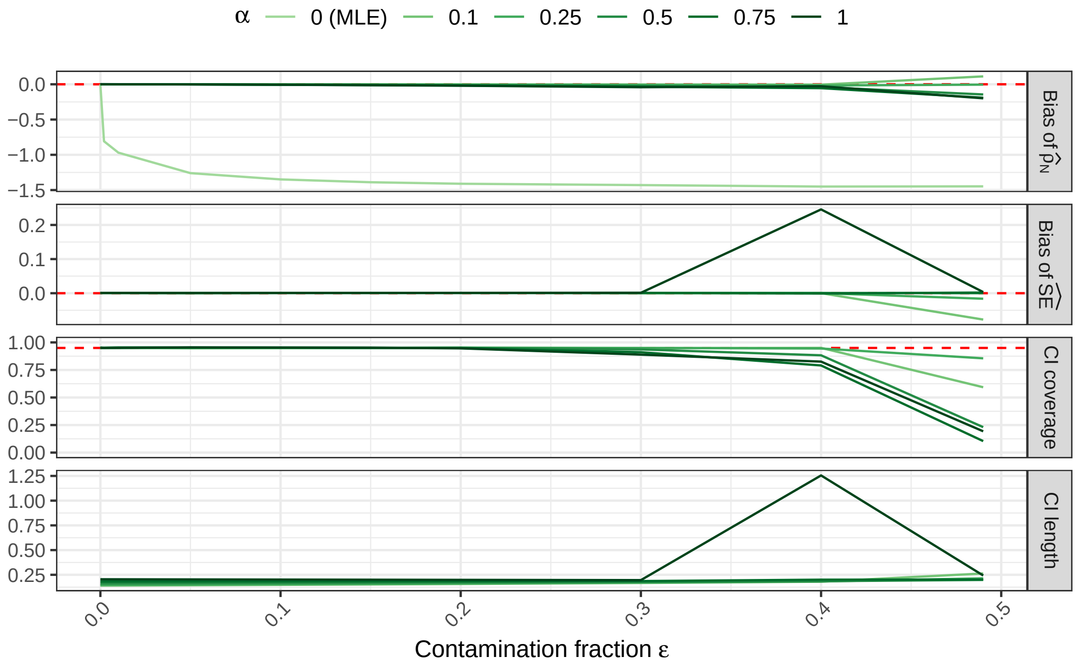


(b) Performance measures of $\widehat{\rho}_N$. For $\varepsilon > 0$, no standard errors (SE) could be computed for ML, so no SE estimation bias, coverage, or confidence interval length could be computed (cf. Figure D.2).

Figure D.1: Visualizations of the results of the "gross error" simulation design in Section D.1 for the considered estimators across 5,000 repetitions, at significance level $\gamma = 0.05$.

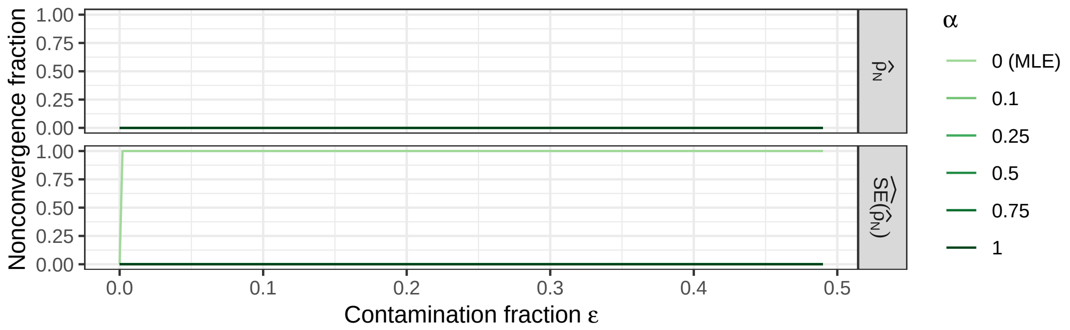


Figure D.2: For the "gross error" design: Fraction of the 5,000 simulation repetitions in which a point estimator did not numerically converge—neither with the unconstrained BFGS nor the constrained Nelder-Mead algorithm—(top), and its associated standard error could not be computed due to singularity of $\widehat{\boldsymbol{J}}_N\left(\widehat{\boldsymbol{\theta}}_N\right)$ in the covariance matrix estimator (bottom). The point estimators always converged, resulting in overplotted lines. For $\varepsilon > 0$, no standard errors could ever be computed for ML, reflecting the instability of ML estimation in the presence of gross errors.

so that the ordinal variable has five response options. For the true polyserial correlation coefficient, we consider $\rho_* \in \{0.5, 0.7, 0.9\}$. Figure D.3 visualizes an example data set generated by this process. For the weakest considered correlation, $\rho_* = 0.5$, contamination (orange dots) and polyserial model points (gray points) substantially overlap and are therefore hard to distinguish. Conversely, the stronger the correlation $\rho_*$, the more distinct the contamination becomes from polyserial model points. We therefore expect that the robustness gain of the robust estimator should increase with stronger true correlations.

We generate $N = 500$ observations from the described process and repeat this procedure 5,000 times. The same performance measures and estimators as in Section 7 are used. We stress that this simulation design has been used before by Welz et al. (2026) in the context of the polychoric correlation model.

Figure D.4 illustrates the simulation results. In the weakest considered correlation size ($\rho_* = 0.5$), the magnitude of bias is similar for all considered estimators and the robust methods only provides a minor improvement compared to the MLE. For the stronger true correlations, the robust estimators yields a notable gain in robustness, particularly for the strongest considered correlation of $\rho_* = 0.9$. It seems plausible that this gain in robustness is driven by contamination being better distinguishable from regular data points when the true correlation that is being shifted has larger magnitude (Figure D.3). Furthermore, at contamination fraction $\varepsilon = 0.49$, all considered estimators converge to similar biases across all considered correlations. Specifically, they converge to the value $-\rho_*$, which indicates that a correlation of nearly 0 was estimated. It is not surprising that for two approximately equally-sized groups of data points with the same mean but whose respective population correlations are mutually sign-flipped, the best fit can be achieved at a correlation estimate of about 0.

Figure D.5 summarizes numerical nonconvergence. Occasional non-computability of standard errors only occurs in the high correlation setting of $\rho_* = 0.9$. Most nonconvergent cases occur with $\alpha = 1$ at $\varepsilon = 0.1$, where about 25% of the standard errors cannot be computed.

Overall, this simulation study demonstrates that the magnitude of our proposed estimator's robustness gain depends on the overlap between the contamination distribution $H_{X\eta}$ and true normal distribution $P_{X\eta}(\cdot, \cdot; \boldsymbol{\theta}_*)$. If these distributions substantially overlap—like in correlation-shifted contamination with weak to moderate true correlation—then the robust estimator may not be able to clearly distinguish

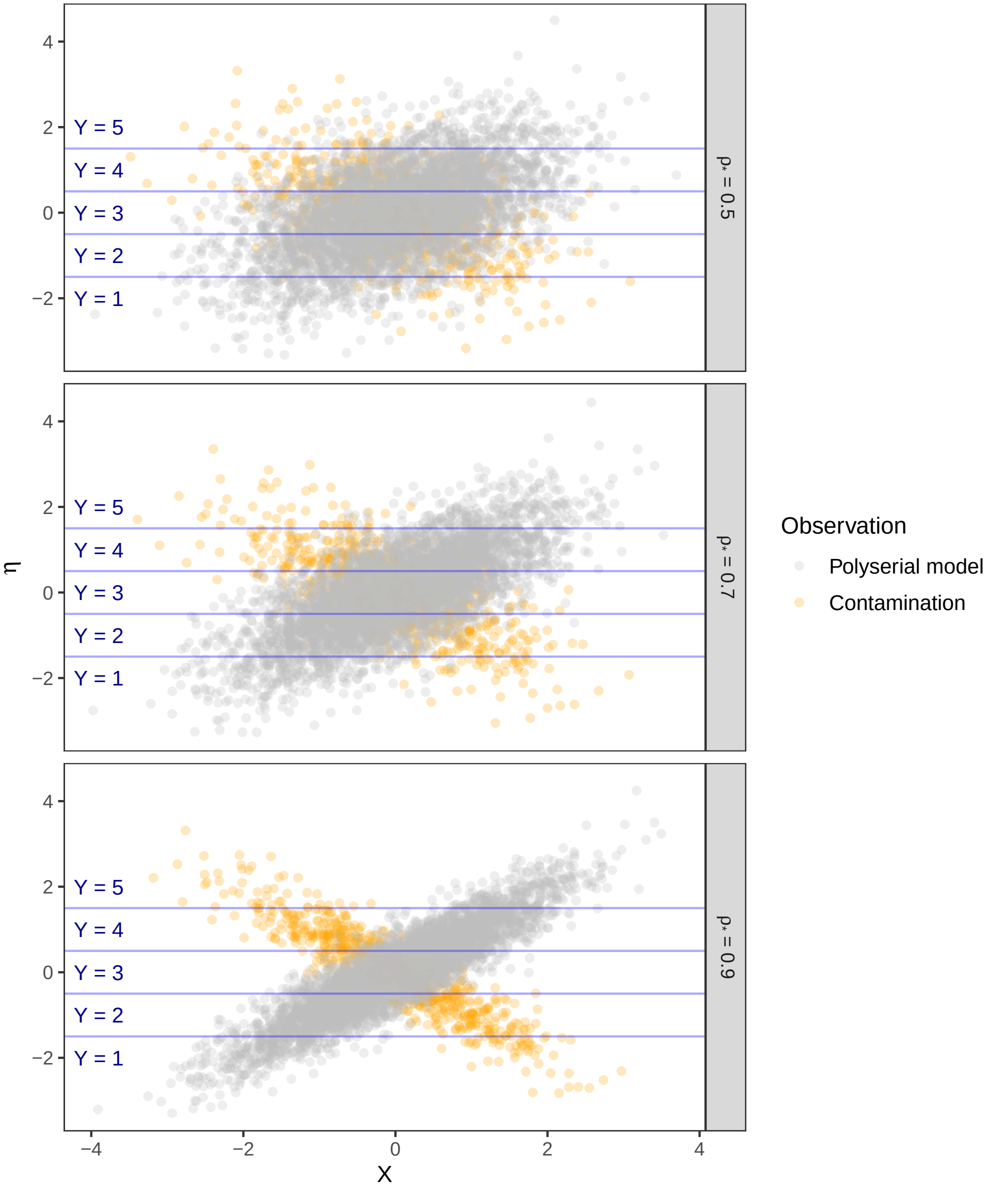


Figure D.3: Example data sets generated by the process with correlation-shifted combination described in Section D.2, for three true correlations $\rho_* \in \{0.5, 0.7, 0.9\}$ (rows) with contamination fraction $\varepsilon = 0.15$. The remaining true parameters are set to $\mu_* = 0, \sigma_*^2 = 1, \tau_{*,1} = -1.5, \tau_{*,2} = -0.5, \tau_{*,3} = 0.5, \tau_{*,4} = 1.5$. Gray dots indicate random draws from the true normal distribution $P_{X\eta}(\cdot, \cdot; \boldsymbol{\theta}_*)$, while orange dots indicate draws from the correlation-shifted contamination distribution $H_{X\eta}$. Horizontal lines denote the discretization thresholds. For a clearer visualization, the sample size is set to $N = 5,000$ in this example data set.

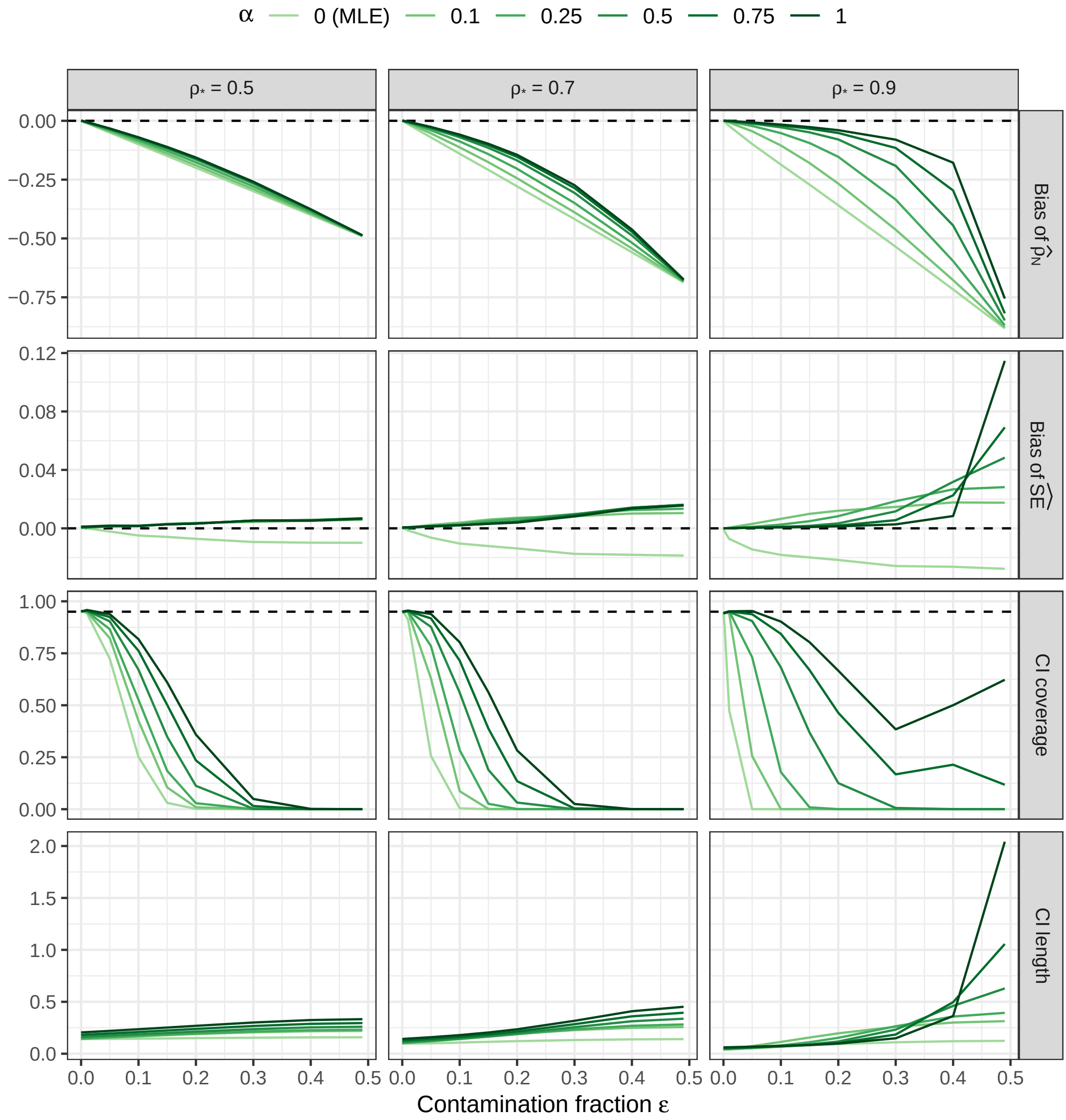


Figure D.4: Performance measures for the simulation in Section D.2 with correlation-shifted contamination, averaged across the 5,000 repetitions, for true correlation coefficients $\rho_* \in \{0.5, 0.7, 0.9\}$ (rows) and different estimators (solid colored lines). The performance measures are the bias of the correlational estimates ($\widehat{\rho}_N^{\text{ave}} - \rho_*$), the bias of their estimated standard errors $\left(\widehat{\text{SE}}^{\text{ave}}(\widehat{\rho}_N) - \text{SE}^{\text{approx}}(\widehat{\rho}_N)\right)$, coverage of the 95% confidence intervals (with the nominal level as dashed horizontal line), and the average length of the confidence intervals. Cases for which no standard errors could be computed due to singularity were excluded from the inferential performance measures; see Figure D.5 for a summary.

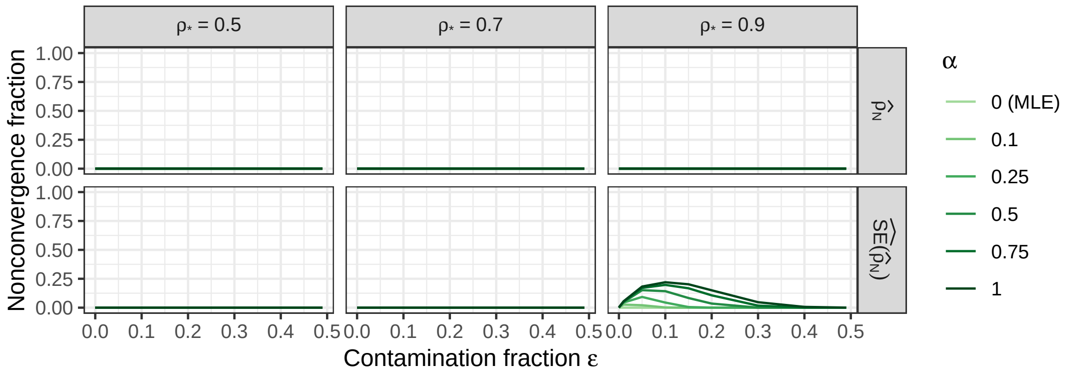


Figure D.5: For the "correlation shift" design: Fraction of the 5,000 simulation repetitions in which a point estimator did not numerically converge—neither with the unconstrained BFGS nor the constrained Nelder-Mead algorithm—(top), and its associated standard error could not be computed due to singularity of $\widehat{\boldsymbol{J}}_N\left(\widehat{\boldsymbol{\theta}}_N\right)$ in the covariance matrix estimator (bottom). The point estimators always converged, resulting in overplotted lines.

between contamination and regular observations since it makes no assumptions on the former, and is subsequently unable to provide a tangible robustness gain. On the other hand, when $H_{X\eta}$ and $P_{X\eta}(\cdot,\cdot;\boldsymbol{\theta}_*)$ are sufficiently distinct—like in correlation-shifted contamination with strong true correlation or mean-shifted correlation (cf. Sections 7 and D.1)—then the robust estimator can identify the contaminated observations and subsequently downweigh them to achieve considerable robustness gains. In either scenario, we observe that the robust estimator is always at least as accurate as the ML estimator, and therefore constitutes an overall improvement in terms of robustness. Similar findings for a different robust estimator in the context of estimating polychoric correlation have been reported by Welz et al. (2026).

### *D.3. Distributional misspecification*

#### D.3.1. Preliminaries

The estimator proposed in this paper is designed to be robust against *partial* misspecification, which is characterized by the polyserial model being misspecified for only a (possibly empty) subset of the observed sample (Section 4). In contrast, in the framework of *distributional* misspecification, the model is misspecified for *all* observations in a sample, not just a subset thereof. Distributional misspecification of the polyserial model manifests in the observed-latent variable pair $(X,\eta)$ being jointly distributed according to an unknown and unspecified nonnormal distribution $G = G_{X,\eta}$. In this framework, the object of interest is the population correlation between $X$ and $\eta$ under the distribution $G$, that is, $\rho_G = \mathbb{C}\mathrm{or}_G\,[X,\ \eta]$, instead of the polyserial correlation coefficient. Estimators for situations where $G$ is nonnormal have been proposed by Bedrick and Breslin (1996), Lord (1963), and Brogden (1949). All of these estimators are based on normality-based maximum likelihood, but are less prone to distributional misspecification when the sampling distribution is not normal.

Although conceptually different, robustness to partial and distributional misspecification frameworks are *"practically synonymous notions"* (Huber & Ronchetti, 2009, p. 4). Thus, despite our robust estimator being designed for a fundamentally different type of model misspecification—partial rather than

distributional misspecification—it is a relevant question whether or not our estimator can also offer enhanced robustness to distributional misspecification. Welz et al. (2026, p. 32) argue that the potential for robustness gains of partial-misspecification-robust estimators in distributional misspecification depends on the properties of the unknown sampling distribution $G$. If the nonnormal $G$ can be decently approximated by mixture between a normal distribution and some other distribution—thereby approximating the partial misspecification framework in (4.1)—then our robust estimator should perform reasonably well. If $G$ cannot be approximated by such a mixture, neither ML nor our estimator may be expected to perform well.

To investigate the performance of our robust estimator when the polyserial model is distributionally misspecified, we perform a simulation study in the following subsection.

### D.3.2. Simulation study

To simulate data for which the polyserial model is distributionally misspecified, we aim to sample from a nonnormal bivariate distribution $G$ with a prespecified value of the population correlation $\rho_G = \mathbb{C}\mathrm{or}_G[X, \eta]$. This is exactly what the VITA simulation method of Grønneberg and Foldnes (2017) does. For a prespecified value of $\rho_G$, the VITA method models the random vector $(X, \eta)$ so that the individual variables $X$ and $\eta$ have correlation $\rho_G$, are both marginally normally distributed, but are *not* jointly normally distributed. Instead, the joint distribution $G$ is set to a prespecified nonnormal copula distribution. Grønneberg and Foldnes (2017) show that the VITA method is particularly useful when VITA-modeled variables are discretized because the ensuing ordinal variables could not have been generated by a latent bivariate normal distribution, as assumed by e.g., polychoric correlation.

In our simulation, we use the VITA method to generate data for the variables $(X, \eta)$ so that their joint distribution is a Clayton or Gumbel copula with population correlation $\rho_G = 0.7$, and $X$ and $\eta$ are both marginally standard normal. Figure D.6 visualizes these copulas. We then discretize the latent variable $\eta$ according to (3.1) with discretization thresholds $\tau_1 = 0, \tau_2 = 1, \tau_3 = 1.5, \tau_4 = 2$, so that the ensuing observed ordinal variable $Y$ has five response options. We generate $N = 500$ observations for $(X, Y)$ according to this process and repeat this procedure 5,000 times. As implementation of the VITA method, we use the package `covsim` (Grønneberg et al., 2022). The same performance measures as in Section 7 are used.

To summarize, this simulation emulates a situation where misspecification of the polyserial model would not be detectable by testing for marginal nonnormality of the observed $X$ by, for instance, a Kolmogorov-Smirnov test, because $X$ is marginally normal. Hence, the simulated situation might be seen as a challenging one for any normality-based estimator because the distributional misspecification only manifests in the unobserved joint distribution.

Figure D.7 and Table D.1 summarize the simulation results. When the joint distribution $G$ is a Clayton copula, the MLE exhibits a bias of about $-0.066$, whereas all robust estimators are less biased (in absolute magnitude). The MLE's coverage is about 60%, but it also has the widest confidence intervals (wider then the robust estimators). In contrast, despite having narrower confidence intervals, the estimators with $\alpha > 0.1$ achieve a coverage of about 60% or higher. Especially the choices $\alpha = 0.5, 0.75$ are remarkably accurate with coverages of about 95% and 87%, respectively. Conversely, when distribution $G$ is a Gumbel copula, all estimators exhibit a notable bias of similar magnitude. The slightly higher coverage of the robust estimators is primarily due to wider confidence intervals. Thus, in this setting, the robust estimators do not improve upon the MLE.

We noticed that in a small number of repetitions, the robust estimator did not numerically converge. This situation occurs when multiple response options of $Y$ are almost never chosen (e.g., only once or twice in the sample). In such a situation, the robust estimator may attempt to eliminate a threshold

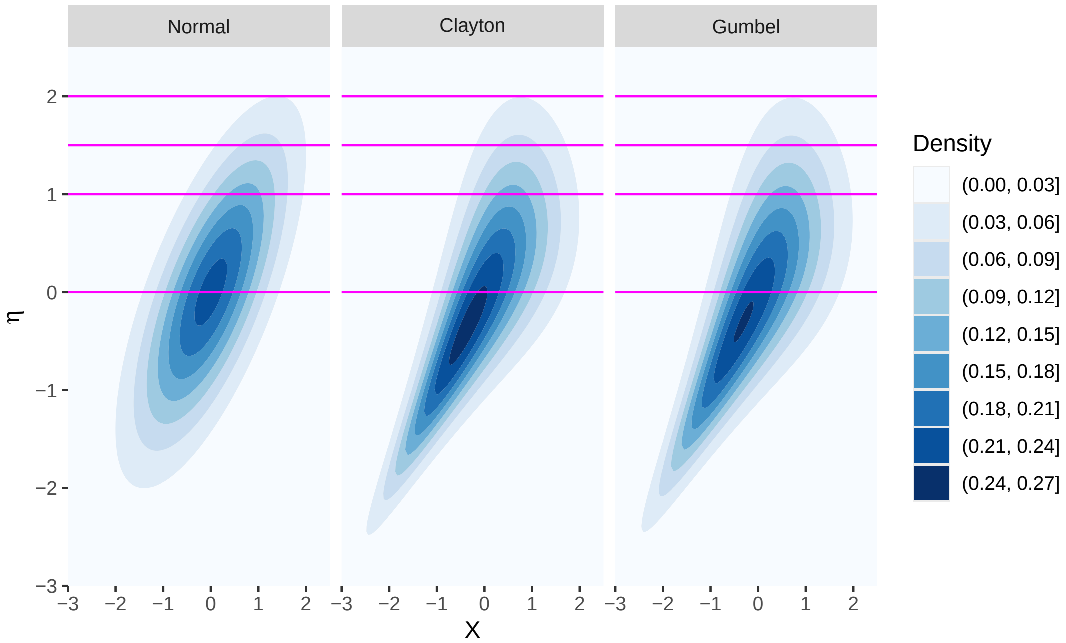


Figure D.6: Bivariate densities of the standard bivariate normal distribution, Gumbel copula, and Clayton copula with correlation $\rho_G = 0.7$ and standard normal marginal distributions. The horizontal lines indicate the thresholds with which the latent $\eta$ is discretized to the ordinal $Y$.

by either pushing the first or last threshold to $\pm\infty$ or pulling adjacent thresholds together as closely as numerically possible. Similar behavior has been observed for a robust estimator of the polychoric correlation model (Welz et al., 2026). We follow Welz et al. (2026) by identifying numerical instability through adjacent thresholds being unreasonably far from each other, namely by a minimum distance of 3.92. Under the polyserial model, this distance covers as much as 95% of all probability mass of the standard normal marginal distribution of $\eta$. We subsequently omitted the estimates where such nonconvergence occurs from the analyses in this section. Figure D.8 summarizes the numerical nonconvergence statistics. The highest fraction of nonconvergent cases occurs for the choice $\alpha = 0.75$ with about 4%, which is a reasonably small value considering that our proposed estimator is not designed for distributional misspecification.

Overall, the results suggest that for population correlation 0.7, the Clayton copula might be reasonably well-approximable by a mixture between a normal distribution and some other distribution, but not the Gumbel copula. Figure D.6 indicates that while the two copulas are of comparable shape, the Clayton copula has substantially more probability mass in its center than the Gumbel distribution, which may enable it to be somewhat approximable by a mixture involving a normal distribution. A similar result for the Clayton copula has been found in Welz et al. (2026) for distributional misspecification of the polychoric correlation model.

To conclude, this simulation study demonstrates that in certain situations with distributional misspecification of the polyserial model, our estimator can offer enhanced robustness compared to the MLE. On the other hand, it also demonstrates that there are situations where no robustness can be gained because

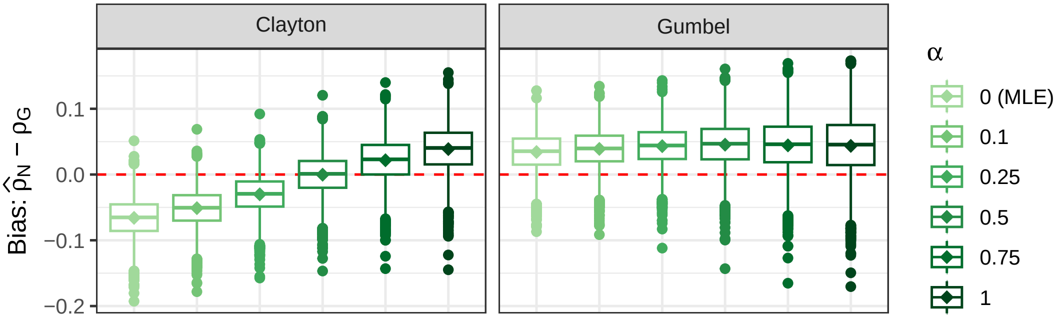


Figure D.7: Boxplot visualization of the bias of the considered bias, $\widehat{\rho}_N - \rho_G$, for the polyserial correlation coefficient under distributional misspecification through Clayton and Gumbel copulas, for 5,000 repetitions and true correlation $\rho_G = 0.7$. Diamonds represent average biases.

| | | Point estimate | | | Standard error | | Confidence interval | |
|---|---|---|---|---|---|---|---|---|
| Distribution $G$ | $\alpha$ | $\widehat{\rho}_N$ | Bias | SE | $\widehat{\text{SE}}$ | Bias | Coverage | Length |
| Clayton | 0 (MLE) | 0.634 | −0.066 | 0.003 | 0.037 | 0.007 | 0.613 | 0.145 |
| | 0.1 | 0.649 | −0.051 | 0.029 | 0.029 | 0.000 | 0.597 | 0.113 |
| | 0.25 | 0.670 | −0.030 | 0.029 | 0.029 | 0.000 | 0.851 | 0.113 |
| | 0.5 | 0.700 | 0.000 | 0.031 | 0.031 | 0.000 | 0.947 | 0.120 |
| | 0.75 | 0.722 | 0.022 | 0.033 | 0.033 | 0.000 | 0.871 | 0.130 |
| | 1 | 0.739 | 0.039 | 0.036 | 0.036 | 0.000 | 0.774 | 0.142 |
| Gumbel | 0 (MLE) | 0.734 | 0.034 | 0.029 | 0.022 | −0.007 | 0.600 | 0.087 |
| | 0.1 | 0.739 | 0.039 | 0.029 | 0.029 | 0.000 | 0.690 | 0.113 |
| | 0.25 | 0.743 | 0.043 | 0.030 | 0.030 | 0.000 | 0.665 | 0.118 |
| | 0.5 | 0.745 | 0.045 | 0.035 | 0.034 | 0.000 | 0.690 | 0.135 |
| | 0.75 | 0.744 | 0.044 | 0.040 | 0.004 | 0.000 | 0.743 | 0.157 |
| | 1 | 0.744 | 0.044 | 0.046 | 0.046 | 0.001 | 0.788 | 0.182 |

Table D.1: Performance measures for estimating polyserial correlation coefficients under distributional misspecification, at significance level $\gamma = 0.05$ (averaged across 5,000 repetitions).

MLE and robust estimators perform very similarly. Thus, while distributional misspecification is not covered by the partial misspecification framework for which our robust estimator is intended, in certain situations our estimator can yield a robustness gain under distributional misspecification, thereby constituting an overall gain in robustness compared to ML estimation.

As a practical consequence, we recommend applied researchers to stay wary of potential partially-latent nonnormality even when ML and our robust estimator produce very similar point estimates. While such similarity may be due to normality holding true (in which case both estimators yield asymptotically equivalent estimates), it may also be due to distributional misspecification from a specific nonnormal distribution for which the robust estimator cannot improve over ML (such as the Gumbel copula considered in this simulation).[1] To investigate the possibility of distributional misspecification

[1]A third possibility is that partially-latent normality is violated in the $\eta$-dimension, but the misspecification is inconsequential because the ensuing density of the observed $(X, Y)$ is equal to the polyserial model distribution in (3.4) (see Section 4). In this case, both ML and robust

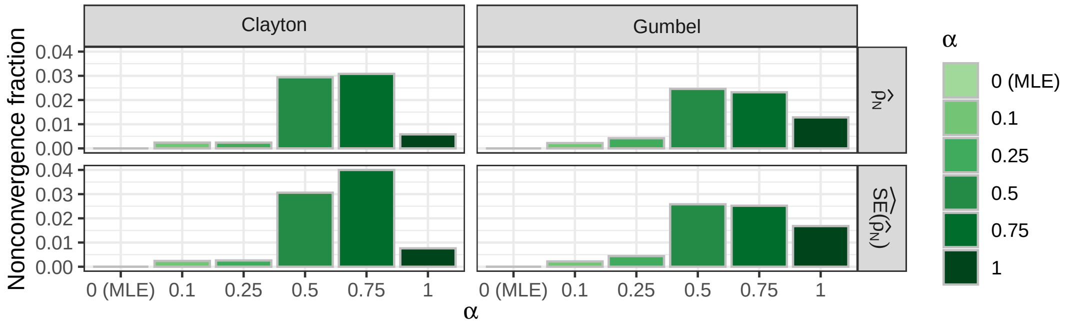


Figure D.8: For the "distributional misspecification" design: Fraction of the 5,000 simulation repetitions in which a point estimator did not numerically converge—neither with the unconstrained BFGS nor the constrained Nelder-Mead algorithm—(top), and its associated standard error could not be computed due to singularity of $\widehat{\boldsymbol{J}}_N\left(\widehat{\boldsymbol{\theta}}_N\right)$ in the covariance matrix estimator (bottom).

whenever ML and robust estimator yield similar estimates, a $\chi^2$-test for partially-latent normality may be considered, which is, for example, implemented in the R package `polycor` (Fox, 2022).[2]

estimators will yield accurate point estimates despite the model being technically misspecified. Hence, in this theoretical case, there are no adverse effects for point estimation with either estimator. We refer to Grønneberg et al. (2020) and Foldnes and Grønneberg (2020) for detailed discussions on testing for underlying normality from discretized variables.

[2]Recently, Foldnes and Grønneberg (2020) have proposed a bootstrap test for testing underlying multivariate normality when *all* observed variables are ordinal. Therefore, the bootstrap test as described in Foldnes and Grønneberg (2020) is not directly applicable to mixed data. However, we believe that it should be possible to extend their methodology to mixed data. We leave this to future research.

## References

Alfons, A., Croux, C., & Filzmoser, P. (2017). Robust maximum association estimators. *Journal of the American Statistical Association*, *112*(517), 436–445. https://doi.org/10.1080/01621459.2016.1148609

Basu, A., Harris, I. R., Hjort, N. L., & Jones, M. C. (1998). Robust and efficient estimation by minimising a density power divergence. *Biometrika*, *85*(3), 549–559. https://doi.org/10.1093/biomet/85.3.549

Bedrick, E. J., & Breslin, F. C. (1996). Estimating the polyserial correlation coefficient. *Psychometrika*, *61*(3), 427–443. https://doi.org/10.1007/BF02294548

Brogden, H. E. (1949). A new coefficient: Application to biserial correlation and to estimation of selective efficiency. *Psychometrika*, *14*(3), 169–182. https://doi.org/10.1007/BF02289151

Fernández, D., Liu, I., Costilla, R., & Gu, P. Y. (2020). Assigning scores for ordered categorical responses. *Journal of Applied Statistics*, *47*(7), 1261–1281. https://doi.org/10.1080/02664763.2019.1674790

Foldnes, N., & Grønneberg, S. (2020). Pernicious polychorics: The impact and detection of underlying non-normality. *Structural Equation Modeling: A Multidisciplinary Journal*, *27*(4), 525–543. https://doi.org/10.1080/10705511.2019.1673168

Fox, J. (2022). *polycor: Polychoric and polyserial correlations* [R package version 0.8-1]. https://CRAN.R-project.org/package=polycor

Grønneberg, S., & Foldnes, N. (2017). Covariance model simulation using regular vines. *Psychometrika*, *82*, 1035–1051. https://doi.org/10.1007/s11336-017-9569-6

Grønneberg, S., Foldnes, N., & Marcoulides, K. M. (2022). covsim: An R package for simulating non-normal data for structural equation models using copulas. *Journal of Statistical Software*, *102*(3), 1–45. https://doi.org/10.18637/jss.v102.i03

Grønneberg, S., Moss, J., & Foldnes, N. (2020). Partial identification of latent correlations with binary data. *Psychometrika*, *85*(4), 1028–1051. https://doi.org/10.1007/s11336-020-09737-y

Huber, P. J. (1967). The behavior of maximum likelihood estimates under nonstandard conditions. In L. M. Le Cam & J. Neyman (Eds.), *Proceedings of the Fifth Berkeley Symposium on Mathematical Statistics and Probability* (pp. 221–234, Vol. 5.1). University of California Press.

Huber, P. J., & Ronchetti, E. M. (2009). *Robust statistics* (2nd). Wiley. https://doi.org/10.1002/9780470434697

Ivanova, A., & Berger, V. W. (2001). Drawbacks to integer scoring for ordered categorical data. *Biometrics*, *57*(2), 567–570. https://doi.org/10.1111/j.0006-341X.2001.00567.x

Lehmann, E. L., & Casella, G. (1998). *Theory of point estimation* (2nd). Springer. https://doi.org/10.1007/b98854

Lord, F. M. (1963). Biserial estimates of correlation. *Psychometrika*, *28*(1), 81–85. https://doi.org/10.1007/BF02289550

Muthén, B. (1984). A general structural equation model with dichotomous, ordered categorical, and continuous latent variable indicators. *Psychometrika*, *49*(1), 115–132. https://doi.org/10.1007/BF02294210

Nocedal, J., & Wright, S. J. (2006). *Numerical optimization* (2nd). Springer. https://doi.org/10.1007/978-0-387-40065-5

Olsson, U., Drasgow, F., & Dorans, N. J. (1982). The polyserial correlation coefficient. *Psychometrika*, *47*(3), 337–347. https://doi.org/10.1007/BF02294164

Rosseel, Y. (2012). lavaan: An R package for structural equation modeling. *Journal of Statistical Software*, *48*(2), 1–36. https://doi.org/10.18637/jss.v048.i02

Welz, M. (2026). Robust estimation of polyserial correlation coefficients: A density power divergence approach [arXiv:2510.15632]. https://doi.org/10.48550/arXiv.2510.15632

Welz, M., Mair, P., & Alfons, A. (2026). Robust estimation of polychoric correlation [Forthcoming.]. *Psychometrika*. https://doi.org/10.1017/psy.2025.10066

White, H. (1982). Maximum likelihood estimation of misspecified models. *Econometrica*, *50*(1), 1–26. https://doi.org/10.2307/1912526
